# Physical, psychological, and behavioral problems among children and adolescents in countries with different economic statuses during the COVID-19 pandemic: a systematic review and meta-analysis

**DOI:** 10.3389/fped.2023.1181186

**Published:** 2023-06-05

**Authors:** Bo Peng, Kara K. L. Reeves, Shara W. Y. Lee, Tina H. Y. Chung, Heidi W. L. Hui, Alfred H. L. Leung, Johnson C. Y. Pang

**Affiliations:** ^1^ School of Clinical Medicine, Chengdu University of Traditional Chinese Medicine, Chengdu, China; ^2^ Department of Rehabilitation Medicine, Sichuan Provincial People's Hospital, University of Electronic Science and Technology of China, Chinese Academy of Sciences Sichuan Translational Medicine Research Hospital, Chengdu, China; ^3^ School of Health Sciences, Caritas Institute of Higher Education, Hong Kong SAR, China; ^4^ Research Rehab Centre Limited, Hong Kong SAR, China; ^5^ Department of Health Technology and Informatics, The Hong Kong Polytechnic University, Hong Kong SAR, China

**Keywords:** COVID-19, psychological problems, behavioral problems, physical activity, sleep problems, children and adolescents

## Abstract

**Introduction:**

The COVID-19 pandemic has impacted children and adolescents’ physical activity (PA), sleeping patterns, and psychological and behavioral health. Yet, little is known about the differences between those in countries with various economic statuses.

**Methods:**

Articles published from database inception through 16 March 2022 were retrieved using CINAHL Complete, Cochrane Library, EMBASE, Medline, PubMed, and PsycINFO. High-quality studies that reported the number of participants with parameters associated with PA, sleeping patterns, and psychological and behavioral problems in young people aged under 18 years during the pandemic were included. We referenced the Canadian 24-Hour Movement Guidelines for PA and sleep duration to provide the event rate for young people who were not compliant with the guidelines. The event rate of young people who had decreased sleep quality and experienced psychological and behavioral problems were also investigated. A subgroup analysis was conducted to identify the differences in those in countries with diverse economic statuses. Funnel plot analysis and Egger's test were also conducted to identify any risk of publication bias.

**Result:**

A total of 66 studies with 1,371,168 participants aged between 0 and 18 years, involving 27 countries, were included. During the pandemic, we identified that 41% (95% CI: 39%, 43%; *I*^2 ^= 96.62) and 43% (95% CI: 34%, 52%; *I*^2 ^= 99.42) of young people did not meet the PA and sleep duration recommendation guidelines. In addition, 31% (95% CI: 28%, 35%; *I*^2 ^= 99.66) of young people had decreased their sleep quality. Yet, no significant difference was found across countries with different economic statuses. However, the event rates of participants with psychological and behavioral problems were 32% (95% CI: 28%, 36%; *I*^2 ^= 99.85) and 19% (95% CI: 14%, 25%; *I*^2 ^= 99.72), respectively. In addition, the rate of psychological problems was more severe in those who live in lower middle-income countries (*p* < 0.001), while the rate of behavioral problems was more severe in those who live in high-income countries (*p* = 0.001).

**Discussion:**

During the pandemic, the discouragement of PA, poor sleep quality, and high risk of psychological and behavioral problems are concerning. A large number of young people did not comply with the recommendation guidelines. Timely implementation of recovery plans is critical to address the adverse effects on young people.

**Systematic review registration:**

https://www.crd.york.ac.uk/prospero/display_record.php?RecordID=309209, identifier CRD42022309209.

## 1. Introduction

The COVID-19 pandemic is considered the most severe health crisis in the century (1) due to its high transmission rate, constant viral evolution and mutation, and high rate of reinfection (2). The emergence of SARS-CoV-2 variants was responsible for sequential waves of infections across the globe (3). As of 8 May 2022, there were 517,072,612 confirmed cases and 6,275,907 deaths reported around the world, affecting over 226 countries and regions (4). In response to the pandemic, many countries have implemented numerous strategies such as social distancing, closure of schools and public places, home isolation, or even lockdown to prevent community transmission. Consequently, the ongoing pandemic has brought severe adverse impacts to the health systems and economies worldwide.

There are a growing number of studies examining the impact of the pandemic on physical activity (PA) and mental health among children and adolescents. A scoping review reported that a reduction in PA was reported in 57 (out of 74) studies in children and adolescents during the pandemic (5). Among the 57 studies, the decrease in time spent on PA ranged between 45 min and 91 min per day (5). Another meta-analysis reported a significant drop in the frequency of moderate-to-vigorous activities and step counts for those aged from 4 to 18 years (6). A decrease in PA has been significantly associated with psychological problems, including anxiety and depression symptoms, in children and adolescents during the pandemic (7, 8). A large meta-analysis reported that the prevalence of anxiety and depression among children and adolescents could reach 19% [95% confidence interval (CI): 14%, 25%, data from 22 studies] and 15% (95% CI: 10%, 21%, data from 16 studies), respectively (9). In addition, social isolation and quarantine induce negative behavioral impacts, such as irritability and inattention (9), sleep disturbance (9), screen time (10), change in dietary habits, and weight gain (11).

Several studies investigated the changes in PA and psychological health during the pandemic (6, 8, 12–15). PA has been shown to positively influence psychological and emotional health, cognitive function, and sleep quality (16). A systematic review indicated that PA improved psychological health in children and adolescents during the pandemic (17). Moreover, a lower level of anxiety, higher level of wellbeing, and better sleep quality were found in active adult participants, compared to those of the less active ones (18). The pandemic has affected the participation of PA in children and adolescents. Yet, PA remained beneficial to psychological health and sleep quality and should be encouraged. To promote participation in PA, Canada has issued the 24-Hour Movement Guidelines for children and youth (19), while the WHO released guidelines on physical activity, sedentary behavior, and sleep for preschool-aged children in 2019 (20) and updated the guidelines for children and adolescents in 2020 (21). Both guidelines recommend ≥180 min spent in a variety of physical activities including ≥60 min spent in moderate-to-vigorous PA for children under 5 years of age and at least 60 min per day of moderate-to-vigorous PA for children and youths over 5 years of age. However, some studies referenced the guidelines as outcome measures for PA during the pandemic (12–24). To the best of our knowledge, there is currently no systematic review and meta-analysis to investigate the rate of children and adolescents who were not meeting the guidelines. As the guidelines are important tools developed by the consensus of multiple experts and popularized in various countries, it is essential to gain insight into the rate of children and adolescents who were not compliant with the guidelines during the pandemic.

We also noticed that health inequities between different countries have undermined the efforts to bring the pandemic under control, including therapeutics, diagnostics, and distribution of vaccines. Historically, the infection and mortality rates were high among the most disadvantaged communities during the health crisis (25). A regression analysis involving 83 countries reported that a greater healthcare capacity was associated with fewer COVID-19 case fatalities [incidence rate ratio (IRR) = 0.5811] (26). Meanwhile, studies revealed that PA has decreased especially with a lower socioeconomic background (5). However, no study to date has investigated the differences between countries’ economic status and PA, sleeping patterns, and psychological and behavioral problems in children and adolescents during the pandemic.

This meta-analysis aimed to identify the event rate of children and adolescents who were not meeting the Canadian 24-Hour Movement Guidelines in PA and sleep duration, as well as the changes in PA levels, sleeping patterns, and psychological and behavioral problems in countries with different economic statuses during the pandemic. Alike to distinctions in infection rate and socioeconomic status, we hypothesized that there would be differences in all the outcomes across countries with different economic statuses. Particularly, the event rate of children and adolescents who were unable to meet the Canadian 24-Hour Movement Guidelines in PA and sleep duration were high during the pandemic. We also hypothesized that the event rates of decreased PA, sleep duration, and poor sleep quality were different among countries with different economic statuses and the event rates of psychological and behavioral problems during the pandemic were different among countries with different economic statuses.

## 2. Method

### 2.1. Search strategy and selection criteria

This meta-analysis adhered to the Preferred Reporting Items for Systematic Review and Meta-Analyses (PRISMA) and Meta-Analysis of Observational Studies in Epidemiology (MOOSE) guidelines. The protocol of this meta-analysis was published in the PROSPERO database (registration number: CRD42022309209). We systematically searched on CINAHL Complete, Cochrane Library, EMBASE, Medline, PubMed, and PsycINFO from database inception to 16 March 2022 without language restrictions for studies with quantitative data involving level of PA, sleeping patterns, and psychological and behavioral problems in children and adolescents (age ≤18 years) during the pandemic. For non-English articles, “Google Translate” was used, followed by a consultation of professional translations by native speakers. To ensure minimal missing data, we manually searched the reference list of all relevant studies. The search history is available in Supplementary Table S1, and the search terms are documented in Supplementary Table S2.

Studies that reported the number of participants with parameters associated with PA, sleeping patterns, and psychological and behavioral problems in young people aged under 18 years during the pandemic were included in this meta-analysis. Studies that involved participants who were not representable for the general young people population, such as studies that exclusively involved participants with pre-existing physiological or psychological health problems or student-athletes, were excluded. Abstracts, editorial comments, and unpublished studies were also excluded.

### 2.2. Risk of bias and certainty assessment

The quality of the included articles was assessed by two independent reviewers with reference to the following six items from the Downs and Black assessment tool adopted by the literature (27–29): (1) clearly stated aim; (2) clearly defined study population; (3) study sample representative of the source population; (4) attempt made to adjust for confounding; (5) attempt made to validate survey responses to intuitional records where possible; and (6) discussion of study limitations. One point was given for an item rated as yes and zero for an item rated as no or unable to determine. Studies that scored 5–6 points, 3–4 points, and 2 or lower points are considered high, moderate, and low quality, respectively. The certainty assessment followed the Grading of Recommendations Assessment, Development and Evaluation (GRADE) approach (30). The GRADE consists of four levels of certainty of evidence: very low, low, moderate, and high. The levels were assessed with five criteria: risk of bias, inconsistency, indirectness, imprecision, and publication bias. Each outcome was assessed by all criteria, and the overall score was taken by the lowest score of each criterion. A third reviewer resolved any disagreements regarding the scoring of the included studies.

### 2.3. Data extraction and statistical analysis

With reference to the inclusion and exclusion criteria, the titles and abstracts for potential studies were screened by two independent reviewers, and the full texts of the remaining articles were evaluated. Disagreements were resolved by a third reviewer. The relevant data were extracted in the included studies by the same reviewers using a standardized data retrieval sheet. The number of participants was extracted and separated into two groups based on their responses to the level of PA, sleeping patterns, and psychological and behavioral problems during the pandemic.

PA level: We followed the Canadian 24-Hour Movement Guidelines to separate participants into either a “meeting recommendation” group or “not meeting recommendation” group if appropriate (19). The guidelines recommend participants aged 1–2 years to have at least 180 min spent in a variety of physical activities at any intensity, including energetic play and spread throughout the day; aged 3–4 years to have at least 180 min spent in a variety of physical activities including at least 60 min spent in moderate-to-vigorous physical activities; and aged 5–17 years to have at least 60 min per day of moderate-to-vigorous physical activity (19). For dichotomous and polychotomous variables that did not report data reflecting the Canadian 24-Hour Movement Guidelines, we separated participants into two groups: decreased and/or no participation in PA and increased and/or no change in participation in PA. The number of participants who had decreased PA, no participation in PA, or not meeting the recommendation guidelines was compared to the number of those who had increased or no change in PA or meeting the recommendation guidelines.

Sleeping pattern: For sleep duration, we followed the Canadian 24-Hour Movement Guidelines (19). The guidelines recommend participants aged 1–2 years to have 11–14 h; aged 3–4 years to have 10–13 h; aged 5–13 years to have 9–11 h; and aged 14–17 years to have 8–10 h of sleep per day (19). We separated the participants into either the “meeting recommendation” group or the “not meeting recommendation” group. For those studies that did not provide data indicating the guidelines, we separated participants into two groups: having changes in sleep duration and no change in sleep duration. For sleep quality, we separated participants into two groups: worsened sleep quality and improved and/or no change in sleep quality. The number of participants who had changes in sleep duration, were not meeting recommendation guidelines, and had decreased sleep quality was compared to the number of those who had no change in sleep duration, were meeting the recommendation guidelines, and had no change or improved sleep quality.

Psychological and behavioral problems: We separated participants who were at risk or had an increase of psychological or behavioral problems into one group and who were at no risk or had a decrease of or no change in psychological or behavioral problems into another group. The number of participants with psychological or behavioral problems was compared to the number of those without psychological or behavioral problems.

In addition, confounding variables including countries’ economic status were assessed by a subgroup analysis. With reference to World Bank (2020), countries’ economic statuses were separated into four income groups: low-, lower middle-, upper middle-, and high-income countries (31). The classifications were based on gross national income per capita in current US dollars with less than 1,036, 1,036–4,045, 4,046–12,535, and more than 12,535 dollars for low-, lower middle-, upper middle-, and high-income countries, respectively (31). The authors of selected articles would be contacted, and any missing data were reported if applicable.

Comprehensive Meta-Analysis version 3.0 (BioStat, Englewood, NJ, United States) was used for statistical analysis. The targeted outcomes were presented as event rates with 95% confidence intervals (CIs) and presented in forest plots. Heterogeneity was reported as *I*^2^, and a random effect model was selected if heterogeneity was 50% or above. The subgroup analysis was performed and compared by countries’ income levels. Funnel plot analysis was implemented to assess the risk of publication bias. Egger's test was conducted to identify any risk of publication bias, with *p* ≤ 0.05 considered a potential publication bias. If publication bias was indicated, the trim-and-fill method was used to recompute the combined effect. A sensitivity analysis was implemented to assess the robustness of all outcomes.

## 3. Results

### 3.1. Search outcomes

**Figure 1:**
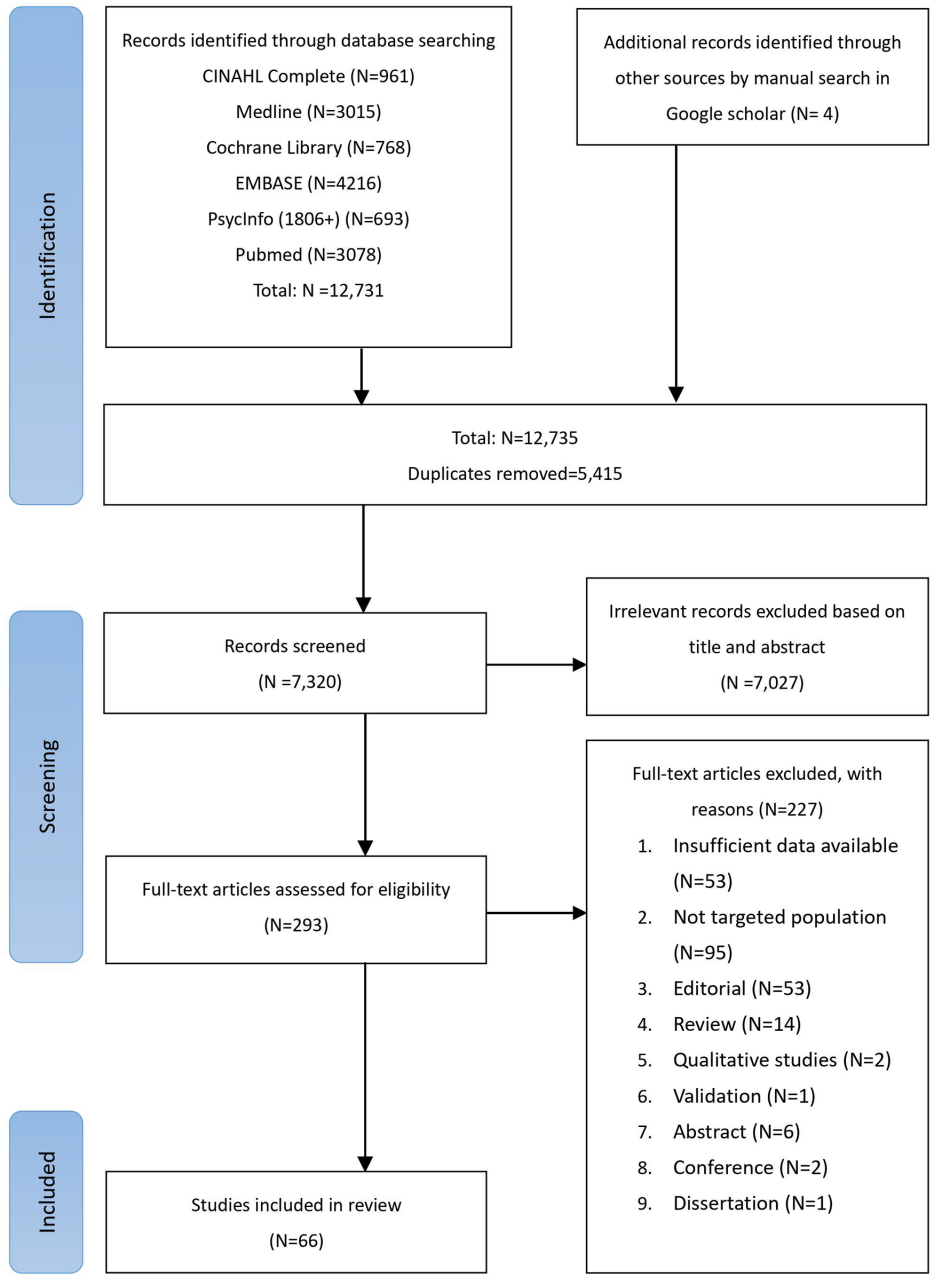
PRISMA flowchart of study selection. PRISMA, Preferred Reporting Items for Systematic Review and Meta-Analyses.

A total of 12,731 studies were retrieved from the databases. After excluding 5,415 duplicated studies, another 7,027 studies were excluded by screening the titles and abstracts. Furthermore, 227 studies were removed after assessing the full tests based on the inclusion and exclusion criteria. Finally, a total of 66 studies were included in our meta-analysis (22–24, 32–94). The PRISMA flowchart of the study selection is presented in Figure 1, and the reasons for exclusion are documented in Supplementary Table S3. No authors were contacted as it was unnecessary in this study.

### 3.2. Characteristics of included studies

A total of 1,371,168 participants aged between 0 and 18 years, involving 27 countries, were included, most of which had the data collection period conducted in 2020. The characteristics of the included studies are described in Table 1. Most studies utilized a validated or self-designed questionnaire as a measuring tool for outcome measurement. Three studies used a wrist-worn accelerometer to measure PA levels or sleep duration (22, 52, 55), and one study employed a direct observational approach to measure PA levels (62). The number of participants, details of the measurement parameters, and tools in each group are tabulated in Supplementary Tables S4–S8.

**Table 1:** Characteristics of included studies.

Authors	Countries	Economic status	Simple size	Mean age (SD)	Age range	Period of data collection
Acosta et al. (2021)	United States	High income	145	Not reported	Primary to high schoolers^a^	October 2020
Alonso-Martínez et al. (2021)	Spain	High income	145	4.29 (0.76)	4–6	March–April 2020
Al-Rahamneh et al. (2021)	Jordan	Upper middle income	1,309	8.1 (2.02)	5–11	10 April–17 April 2021
Alves et al. (2020)	United States	High income	64	11.84 (1.28)	9–15	22 April–29 July 2020
Androutsos et al. (2021)	Greece	High income	397	7.8 (4.1)	2–18	30 April–24 May 2020
Awais et al. (2021)	Pakistan	Lower middle income	225	17.9 (1.22)	15–19	March–May 2020
Azoulay et al. (2021)	Israel	High income	220	11.8 (3.3)	5–18	15 May–15 December 2020
Berasategi et al. (2021)	Basque Autonomous Country (an autonomous community in Northern Spain)	High income	500	Not reported	12–18	11–25 May 2020
Berki and Pikó (2021)	Hungary	High income	705	15.9 (1.19)	14–19	6–20 December 2020
Bingham et al. (2021)	United Kingdom	High income	949	10.5 (1.1)	9–13	Spring 2020
Breidokienė et al. (2021)	Lithuania	High income	306	9.65 (1.94)	6–14	May–June 2020
Brzęk et al. (2021)	Poland	High income	1,316	Not reported	3–5	April–November 2020
Campbell et al. (2021)	United States	High income	761	Not reported	Grades 9–12^a^	30 March–8 May 2020
Chaffee et al. (2021)	United States	High income	485	Not reported	Grades 9–10^a^	August 2019–February 2020
Chi et al. (2021)	China	Upper middle income	1,794	15.26 (0.47)	15–18	13–20 May 2020
Docimo et al. (2021)	Italy	High income	220	9.1 (2.86)	4–14	15 July 2020–15 January 2021
Dragun et al. (2020)	Croatia	High income	531	18.0 (6.0)	Not reported	May 2020
Dubuc et al. (2020)	Canada	High income	2,661	Not reported	Not reported	December 2020
Dunton et al. (2020)	United States	High income	211	8.73 (2.58)	5–13	25 April–16 May 2022
Erades et al. (2020)	Spain	High income	112	6.65 (3.21)	3–13	March 2022
Ezpeleta et al. (2020)	Spain	High income	226	13.9 (0.28)	13–14	13 March and April 2020
Francisco et al. (2020)	Italy, Spain, Portugal	High income	1,480	9.15 (4.27)	3–18	March and April 2022
Ghanamah and Eghbaria-Ghanamah (2021)	Israel	High income	382	Not reported	5–11	4–10 December 2022
Ghorbani et al. (2021)	Iran	Lower middle income	154	16.28 (0.97)	15–17	October 2022–February 2021
Gilbert et al. (2021)	United States	High income	144	8.01 (1.75)	5–12	Late May to early July 2022
Guo et al. (2021)	China	Upper middle income	10,416	Not reported	10–16	8–15 March 2020
Hyunshik et al. (2021)	Japan	High income	290	3.6 (0.3)	3–5	October 2020
James et al. (2021)	United Kingdom	High income	1,068	9.99	11-Sep	20 March–29 June 2020
Jester and Kong (2021)	United Kingdom	High income	55	Median 17^a^	15–18	13 Apr–8 June 2020
Jolliff et al. (2021)	United States	High income	134	14.7^a^	9–11	31 March–3 April 2020
Jovanović et al. (2021)	Croatia	High income	1,370	12.72 (1.17)	10–15	September 2020 and May 2021
Kim S. J. et al. (2021)	South Korea	High income	217	9.16 (1.43)	12–17	1–30 June 2020
Kim S. Y. et al. (2021)	South Korea	High income	52,139	Not reported	12–18	3 August–13 November 2020
Lanza et al. (2021)	United States	High income	361	Not reported	1–12	September–November 2020
Laurier et al. (2021)	Canada	High income	133	15.26 (1.46)	11–17	June–August 2020
Lee D. J. et al. (2021)	South Korea	High income	844	Male, 16.01 (1.58); female, 15.91 (1.38)	14–18	7–15 January 2021
Li et al. (2021)	Australia	High income	760	14.80 (1.26)	12–18	22 June–5 August 2020
Liu et al. (2021)	China	Upper middle income	1264	9.81^a^	7–12	25 February and 8 March 2020
López-Gil et al. (2021)	Brazil	Upper middle income	485	Not reported	3–17	20 April 2020
Lu et al. (2020)	China	Upper middle income	965	15.26^a^	15–17	13 and 20 May 2020
MacKenzie et al. (2021)	Canada	High income	85	Not reported	4–14	June 2020
Medrano et al. (2021)	Spain	High income	113	12.0 (2.6)	8–16	Late March 2020
Mingazova et al. (2021)	Russian Federation	Upper middle	10,237	Grades 1–8^a^	Not reported	May 2020
Mitra et al. (2020)	Canada	High income	1,472	N/A	5–17	April 2020
Mitra et al. (2021)	Canada	High income	800	N/A	9–15	May 2020
Morgül et al. (2020)	United Kingdom	High income	927	7.45^a^	5–11	14 July–14 August 2020
Ng et al. (2020)	Ireland	High income	1,214	N/A	12–18	April 2020
Ng et al. (2021)	Czechia	High income	3,440	13.5^a^	11–15	June 2020
Pombo et al. (2021)	Portugal	High income	2,159	N/A	0–12	23 March–1 April 2020
Qin J. et al. (2021)	China	Upper middle income	248	N/A	13–16	2 April 2020
Qin Z. et al. (2021)	China	Upper middle income	1,199,320	12.04^a^	Not reported	8–30 March 2020
Reséndiz-Aparicio (2021)	Spain	High income	4,000	N/A	4–15	20–26 May 2020
Sá et al. (2020)	Brazil	Upper middle income	816	N/A	<13	25 March–24 April 2020
Salzano et al. (2021)	Italy	High income	1,860	16.0 (1.9)	12–18	23 April–3 May 2020
Schnaiderman et al. (2021)	Argentina	Upper middle income	267	11.1^a^	6.2–18.1	September–October 2020
Siachpazidou et al. (2021)	Greece	High income	482	8.1 (2.2)	4–12	27 November–3 December 2020
Szwarcwald et al. (2021)	Brazil	Upper middle income	9,470	N/A	12–17	27 June–17 September 2020
Tandon et al. (2021)	United States	High income	1,000	10.8 (3.5)	6–17	22 October–2 November 2020
Tornaghi et al. (2021)	Italy	High income	395	N/A	15–18	4–10 April 2020
Ventura et al. (2021)	Catalonia (an autonomous community in the east of Spain)	High income	3,464	N/A	<17	7–18 April 2020
Vuković et al. (2021)	Serbia	Upper middle income	450	N/A	7–15	29 May–6 June 2020
Wang, Hao et al. (2021)	China	Upper middle income	6,906	N/A	6–16	20 May–20 July 2020
Wang, Zhang et al. (2021)	China	Upper middle income	11,072	N/A	6–16	20 May–13 July 2020
Wang, Chen et al. (2021)	China	Upper middle income	12,186	9.1 (1.36), 13.9 (1.39)	6–11, 12–16	20 May–20 July 2020
Zhang et al. (2020)	China	Upper middle income	9,979	11.63 (1.23)	9–14	8–15 March 2020
Zhu et al. (2021)	Hong Kong, China	High income	2,863	12.6 (1.3)	9–17	May 2020

^a^ No exact mean age with SD or age range was reported.

### 3.3. Quality assessment of included studies

The overall quality grading of included studies was moderate to high based on the six items in the Downs and Black assessment tool. Most studies failed to score on item 4, which was related to failure to attempt to adjust for confounding. Some studies did not use validated questionnaires for data collection, therefore failed to score on item 5. As for the certainty of outcomes, all outcomes were graded as moderate, which was mostly due to high inconsistency with *I*^2 ^> 90% and a moderate risk of bias. The Downs and Black assessment of all included studies and the GRADE score of all outcomes are presented in Supplementary Tables S9 and S10, respectively.

### 3.4. Meeting the recommendation guidelines

**Figure 2:**
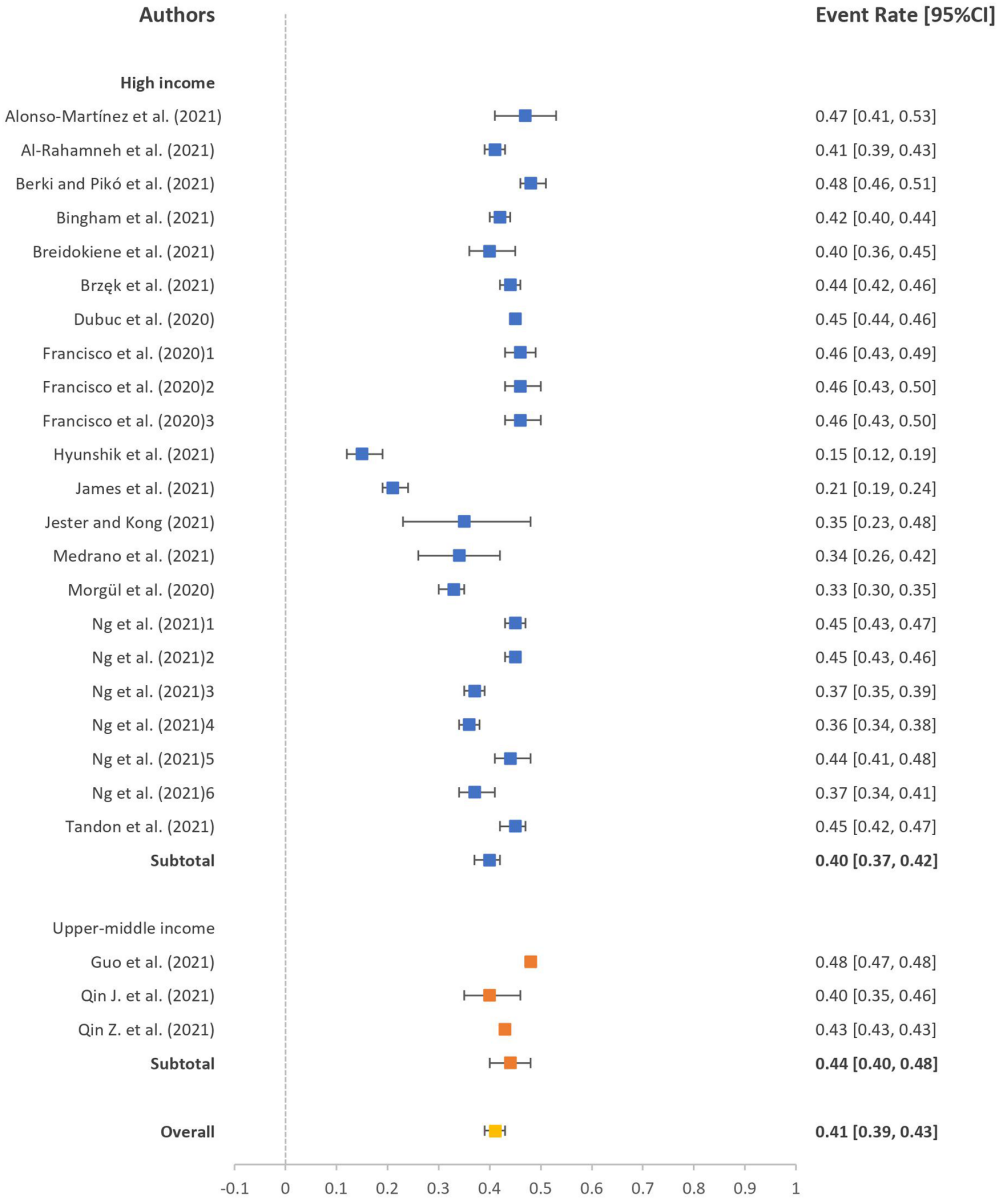
Event rate of participants not meeting PA recommendation guidelines. PA, physical activity.

**Figure 3:**
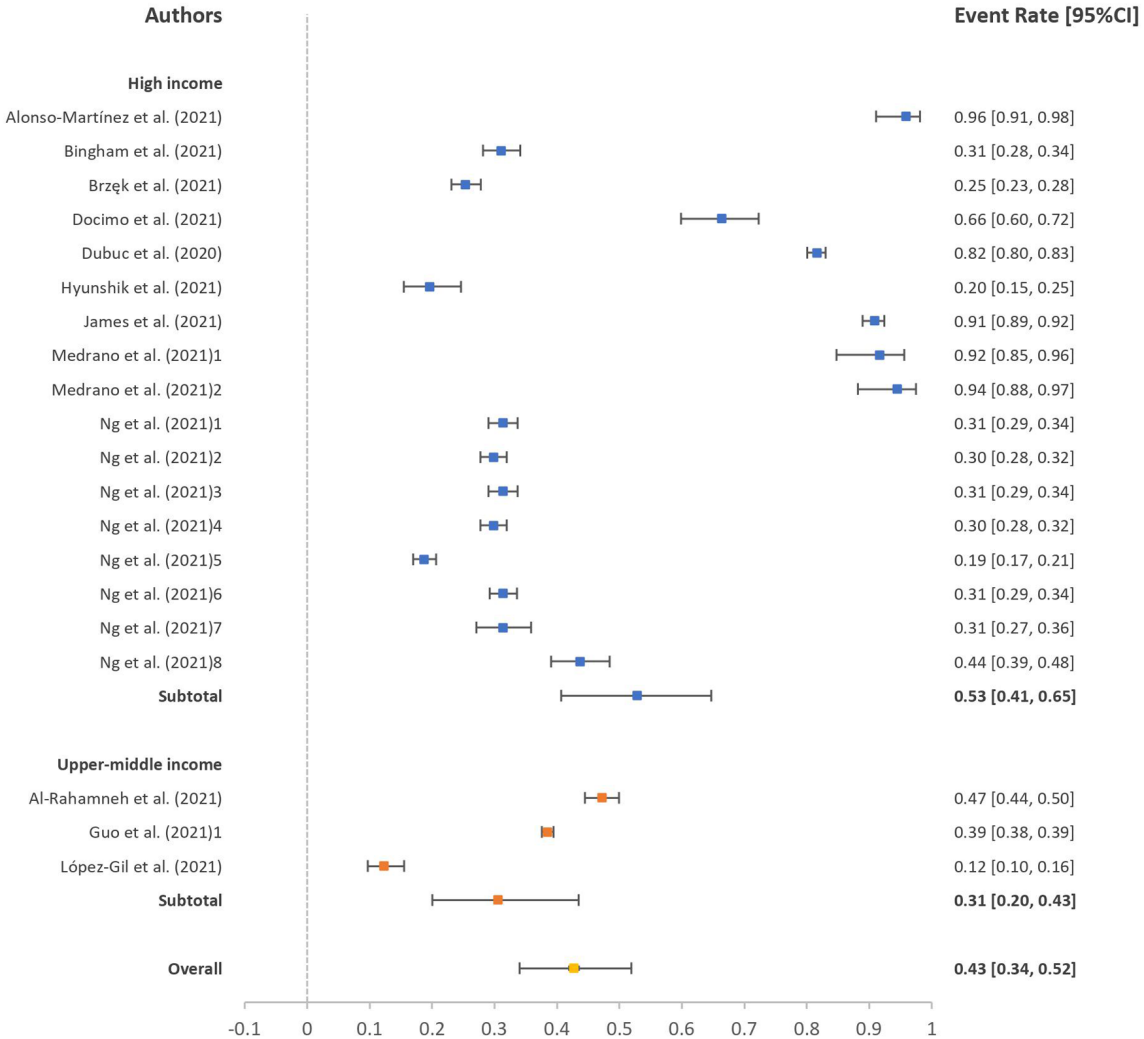
Event rate of participants not meeting sleep duration recommendation guidelines.

The event rates of participants not meeting the recommendation guidelines of PA level and sleep duration during the pandemic are presented in forest plots in Figures 2, 3. The event rates of participants not meeting the PA and sleep duration recommendation guidelines were 41% (95% CI: 39%, 43%; *I*^2 ^= 96.62) and 43% (95% CI: 34%, 52%; *I*^2 ^= 99.42), respectively. In the subgroup analysis, we could only retrieve data for high- and upper middle-income countries. The event rates of participants not meeting PA recommendation guidelines were 40% (95% CI: 37%, 42%; *I*^2 ^= 95.94) for high-income countries and 44% (95% CI: 40%, 48%; *I*^2 ^= 98.78) for upper middle-income countries. Likewise, the event rates of participants not meeting the sleep duration recommendation guidelines were 53% (95% CI: 41%, 65%; *I*^2 ^= 99.48) for high-income countries and 31% (95% CI: 20%, 43%; *I*^2 ^= 98.78) for upper middle-income countries. No significant difference was observed between the income level countries in meeting the recommendation guidelines of PA level (*p* = 0.063) and sleep duration (*p* = 0.172).

### 3.5. Pooled PA levels and pooled sleeping patterns

The pooled event rates of participants with decreased PA, with no participation in PA, or who are not meeting the PA recommendation guidelines and with changes in sleep duration and sleep quality during the pandemic are presented in forest plots in Supplementary Figures S1–S3. Among them, the event rate was 61% (95% CI: 58%, 65%; *I*^2 ^= 99.64) for participants with decreased PA, 34% (95% CI: 26%, 42%; *I*^2 ^= 99.88) for participants with changes in sleep duration, and 31% (95% CI: 28%, 35%; *I*^2 ^= 99.66) for participants with poorer sleep quality. We were able to conduct the subgroup analysis for high-, upper middle-, and lower middle-income countries for the pooled PA level. The event rates of participants with decreased PA levels were 60% (95% CI: 56%, 64%; *I*^2 ^= 99.33) for high-income countries, 63% (95% CI: 55%, 70%; *I*^2 ^= 99.88) for upper middle-income countries, and 79% (95% CI: 10%, 99%; *I*^2 ^= 98.57) for lower middle-income countries. No significant difference was seen among the three income levels (*p* = 0.769). As for the pooled sleep duration and sleep quality, the event rate of participants with changes in sleep duration was 40% (95% CI: 30%, 52%; *I*^2 ^= 99.81) for high-income countries and 25% (95% CI: 16%, 37%; *I*^2 ^= 99.62) for upper middle-income countries, and that of participants with poorer sleep quality was 37% (95% CI: 24%, 51%; *I*^2 ^= 99.73) for high-income countries and 31% (95% CI: 28%, 35%; *I*^2 ^= 97.50) for upper middle-income countries. No significant difference was seen among income level countries in sleep duration (*p* = 0.059) and sleep quality (*p* = 0.397).

### 3.6. Psychological and behavioral problems

The event rates of participants with psychological and behavioral problems during the pandemic are presented in a forest plot (Supplementary Figures S4, S5). The event rates of participants with psychological and behavioral problems were 32% (95% CI: 28%, 36%; *I*^2 ^= 99.85) and 19% (95% CI: 14%, 25%; *I*^2 ^= 99.72), respectively. In the subgroup analysis, the event rates of participants with psychological problems were 45% (95% CI: 38%, 52%; *I*^2 ^= 99.16) for high-income countries, 24% (95% CI: 20%, 29%; *I*^2 ^= 99.91) for upper middle-income countries, and 70% (95% CI: 42%, 88%; *I*^2 ^= 97.57) for lower middle-income countries. We observed a significant difference among all three income levels (*p* < 0.001), between high-income and upper middle-income countries (*p* < 0.001), and between upper middle- and lower middle-income countries (*p* = 0.001). However, there was no significant difference between high-income and lower middle-income countries (*p* = 0.087). For behavioral problems, the event rates of participants with behavioral problems were 27% (95% CI: 19%, 36%; *I*^2 ^= 99.75) for high-income countries and 9% (95% CI: 5%, 16%; *I*^2 ^= 99.66) for upper middle-income countries. Similarly, we identified a significant difference between both income level countries (*p* = 0.001).

### 3.7. Publication bias analysis

Publication bias was evaluated by funnel plot and Egger's test. The funnel plots of all outcomes are presented in Supplementary Figure S6. The funnel plots and Egger's test indicated that there was publication bias among the studies on participants with decreased PA (*p* = 0.008), changes in sleep duration (*p* = 0.001), and psychological problems during the pandemic (*p* < 0.001). However, other outcomes resided in symmetric funnel plots shape and results of Egger's test showed no significant publication bias (PA guidelines, *p* = 0.220; sleep duration guidelines, *p* = 0.270; sleep quality, *p* = 0.432; behavioral problems, *p* = 0.673). After adjustment using the trim-and-fill method, the event rates were 61% (95% CI: 57%, 64%) instead of 61% (95% CI: 58%, 65%) for participants with decreased PA level and 27% (95% CI: 24%, 30%) instead of 32% (95% CI: 28%, 36%) for participants with psychological problems. However, the event rate for changes in sleep duration remained unchanged after the trim-and-fill method.

### 3.8. Sensitivity analysis

The robustness of our results was examined by the leave-out-one sensitivity analysis. Our results were not modified with the stepwise exclusion of each study. More details are displayed in Supplementary Table S1^1^.

## 4. Discussion

The current systematic review and meta-analysis explored the effects of the pandemic on PA, sleep duration, and sleep quality, as well as psychological and behavioral problems among children and adolescents in countries with different economic statuses. We have identified the event rate of children and adolescents who were not compliant with the Canadian 24-Hour Movement Guidelines in PA and sleep duration in countries during the pandemic. We have also provided the event rate of children and adolescents with discouraging PA performance including decreased PA, no participation in PA, or not meeting the recommendation guidelines, changes in sleeping patterns, and psychological and behavioral problems in countries with different economic statuses during the pandemic. To the best of our knowledge, this is the first meta-analysis that objectively examines the effect of the pandemic on these parameters for young people living in different countries with different economic statuses.

Our results supported the hypothesis that the event rates of children and adolescents who were unable to meet the Canadian 24-Hour Movement Guidelines in PA and sleep duration were high during the pandemic. However, although we hypothesized there would be differences in those across countries with different economic statuses, our results had shown otherwise. Decreased PA during the pandemic and failure to comply with the PA recommendation guidelines were reported during the pandemic (5, 95). This was also reflected in our results with 61% (95% CI: 58%, 65%) of children and adolescents with discouraging PA performance and 41% (95% CI: 39%, 43%; *I*^2 ^= 96.62) of children and adolescents who did not meet the PA recommendation guidelines during the pandemic. However, no significant difference was found among countries with high-, upper middle-, and lower middle-income groups for the pooled PA (*p* = 0.769) and compliance with PA recommendation guidelines (*p* = 0.063).

In addition, 43% (95% CI: 34%, 52%; *I*^2 ^= 99.42) of children and adolescents did not meet the guidelines for sleep duration, and 34% (95% CI: 26%, 42%; *I*^2 ^= 99.88) of them had changed, either increased or decreased, their sleep duration during the pandemic. However, there was no significant difference in sleep duration among countries with different economic statuses (*p* = 0.432). These findings were in line with a previous systematic review that reported sleep duration did not change in 43% of participants on weekdays and in 46.2% on weekends (96). During the pandemic, home confinement negatively affected families’ incomes that had relevant impacts on the socioeconomic context, especially among the people who live in low-income countries and suffer from aggravating social and health inequalities (97). It was plausible that preschoolers with lower-income parents were less likely to have adequate PA and poorer sleep quality during the pandemic (98). However, our systematic review and meta-analysis found that people living with different incomes did not show a significant difference in PA. In addition, the pooled event rate of the low sleep quality was 31% (95% CI: 28%, 35%; *I*^2 ^= 99.66). Similarly, there was no significant difference among the countries with different incomes (*p* = 0.307). The results supported that the decrease in PA, sleep duration, and sleep quality in young people is not associated with the incomes of the countries that they are living in. Contrariwise, our results may be biased because there were scanty studies included from lower middle-income countries, and no studies could be found from low-income countries.

A systematic review of recent studies showed that psychological problems were often found during lockdown or social isolation periods (99). Similar findings were observed in our systematic review and meta-analysis in which the pooled event rate of psychological problems was 32% (95% CI: 28%, 36%; *I*^2^ = 99.85). Our results also partially supported the hypothesis that the event rates of psychological and behavioral problems were different among countries with different economic statuses (100–102). In the subgroup analysis, there were significant differences in the event rates between high-income and upper middle-income countries and between upper middle-income and lower middle-income countries for psychological problems (both *p* < 0.001). However, there was no significant difference in the event rate between high-income and lower middle-income countries for psychological problems. Although the inconsistency might be due to the limited number of studies for lower middle-income countries, the event rate for children and adolescents in middle lower-income countries remains the highest at 70% (95% CI: 42%, 88%; *I*^2 ^= 97.57) among the event rates of those in high- and upper middle-income countries. As a result, this meta-analysis provides evidence that the socioeconomic context can influence psychological problems among young people and the mental health needs of children and adolescents were high, especially in middle lower-income countries (103, 104).

Throughout the pandemic, authorities have implemented measures for protection such as social restrictions and self-isolation, which could explain the increased behavioral problems among young people (105). Our findings supported that 19% (95% CI: 14%, 25%; *I*^2 ^= 99.72) of children and adolescents had behavioral problems during the pandemic. Although most countries applied restrictive measures to control the spread of the virus, we found that the influence on behavioral problems among young people in high- and upper middle-income countries differ significantly (*p* = 0.001). Household income is an important part of the family socioeconomic status that is associated with behavior problems of young people (106, 107). Young people living in lower-income countries may have more stress responses induced by fears of infection, lack of personal space, and family financial situation (108, 109), resulting in prolonged effects on mental wellbeing such as an increase in social isolation and mood disturbances (110). As a result, a higher rate of behavioral problems can be found during the pandemic in lower-income countries. However, our findings showed that behavioral problems among young people in high-income countries were unexpectedly higher with 27% (95% CI: 19%, 36%; *I*^2 ^= 99.75) than those among young people in upper middle-income countries with 9% (95% CI: 5%, 16%; *I*^2 ^= 99.66). This may result from the influence of this pandemic and its measures on parents and young people. The diversity of infection rates and different protective measures may contribute more impacts on behavioral problems among young people in high-income countries than in lower-income countries. However, a recent study reported that an increase in income is unrelated to behavioral problems in children and adolescents during the pandemic (111). Furthermore, a meta-analysis revealed that to positively influence the psychological and behavioral health of children and adolescents, a combined intervention including economic support and social care is critical (112). An economic intervention, such as cash allowance, alone was ineffective and inadequate, despite encouraging an increase in spending among children and adolescents, which showed no significant impact on mental health (112). Moreover, it is stated that one in eight children has mental health disorders in high-income countries that require interventions (113). Yet, even in high-income countries, most are not receiving adequate services (113). However, this meta-analysis only identified statistics in high-income countries (113). We novelty compared the event rate in countries with different income levels, and the results were aligned. The rate of young people with behavioral problems was higher in high-income countries. We urge policymakers to assess risk and strategically allocate finite resources for interventions. Particularly, monetary support alone was inadequate, and a holistic approach including public investment and promotion in mental health services and community support is needed. Further studies are recommended to identify the influences of the pandemic on young people's behaviors and propose all-rounded solutions to the impending behavioral problems in the long run.

Furthermore, the reduction in exercise duration (114) and longer periods of sedentary behavior in young people may induce serious health burdens to society (115, 116). Sleep duration and sleep quality closely affect psychological wellbeings and behavioral conduct, such as the ability to control inner states or responses toward thoughts, attention, emotions, or even performance (117, 118). Our findings observed that 32% and 19% of the young population experienced psychological and behavioral problems, respectively, during the pandemic and were associated with the economic status of the country. The crisis of the pandemic induced a sharp reduction of fiscal revenues for different countries, especially in countries with lower middle- and low-income groups, which result in an added burden to the most vulnerable individuals, including children and adolescents (119). The increase in poverty and inequality induced by the pandemic may change the quantity and nature of PA and sleeping patterns among young people. In addition, the rate of psychological and behavioral problems among young people during the pandemic was alarming and urged policymakers, scientists, and practitioners to identify solutions for attenuating the adverse changes (120).

## 5. Limitations

The studies included in this meta-analysis only represented high-, upper middle-, and lower middle-income countries. No study reported our targeted outcomes in low-income countries. The impacts of poverty and inequality induced by the pandemic on the PA, sleeping patterns, psychological wellbeings, and behavioral changes in children and adolescents in low-income countries are unknown. This could be attributed to the limited data retrieved in the included studies that met our inclusion criteria. In addition, although we searched six scientific databases with no language restriction, we expected that the findings may cover nearly all relevant published articles. There was an inevitable possibility that additional relevant articles might have been missed due to the restriction of our search to these six databases. However, we minimized the possibility of missing data by manually searching the reference lists of all relevant studies. Therefore, we believed that the number of studies missing from our meta-analysis was likely small and would have a limited impact on our results. Lastly, our outcomes showed high heterogenicity (*I*^2 ^> 90%). This is expected as the outcome measures used to measure PA, sleeping patterns, and psychological and behavioral problems were diversified. We attempted to control the influences of high heterogenicity by using a random effect model and assessed the publication bias with funnel plots and Egger's test. Moreover, potential publication bias is a factor that should be taken seriously, as it could easily exaggerate the effects when none exists. We have adjusted the results with the trim-and-fill method to lessen the publication bias, but the potential influence of publication bias should, nevertheless, be cautious when interpreting the results, especially for the event rate for changes in sleep duration.

## 6. Conclusion

The discouragement of PA during the pandemic has affected young people negatively. Changes in PA levels, sleep duration, poorer sleep quality, and psychological and behavioral problems were seen among young people in countries with different economic statuses. Moreover, 41% of the young population did not meet the recommendations for PA, and 43% of the young population did not meet the recommendations for sleep duration during the pandemic. In addition, the rate of psychological problems was more severe in those who live in lower middle-income countries, while the rate of behavioral problems was more severe in those who live in high-income countries. Also, studies on the changes in PA, sleep duration, sleep quality, and psychological and behavioral problems in lower middle- and low-income countries were scanty. Further studies are necessary to investigate the impacts of the pandemic on children and adolescents, especially in lower middle- and low-income countries.

## Data Availability

The datasets presented in this study can be found in online repositories. The names of the repository/repositories and accession number(s) can be found in the article/**Supplementary Material**.
