# Supplementary material for: Physical, psychological, and behavioral problems among children and adolescents in countries with different economic statuses during the COVID-19 pandemic: a systematic review and meta-analysis
Source: Front Pediatr. 2023 Jun 5;11:1181186. doi: 10.3389/fped.2023.1181186 (PMC10277820; doi:10.3389/fped.2023.1181186)
Supplement: Supplementary file 1 [file Table1.docx]

Supplementary Material

**Physical, psychological, and behavioural problems among children and adolescents in countries with different economic status during the COVID-19 pandemic: A systematic review and meta-analysis**

Bo Peng^1^, Kara K. L. Reeves^2*^, Shara W. Y. Lee^3^, Tina H. Y. Chung^2^, Heidi Y. L. Hui^2^, Alfred H. L. Leung^2^, Johnson C.Y. Pang^2^

*** Correspondence:** Corresponding Author: kreeves@cihe.edu.hk

# Supplementary Tables

Supplementary Table 1. Search history on 16^th^ March 2022.

| CLNAHL  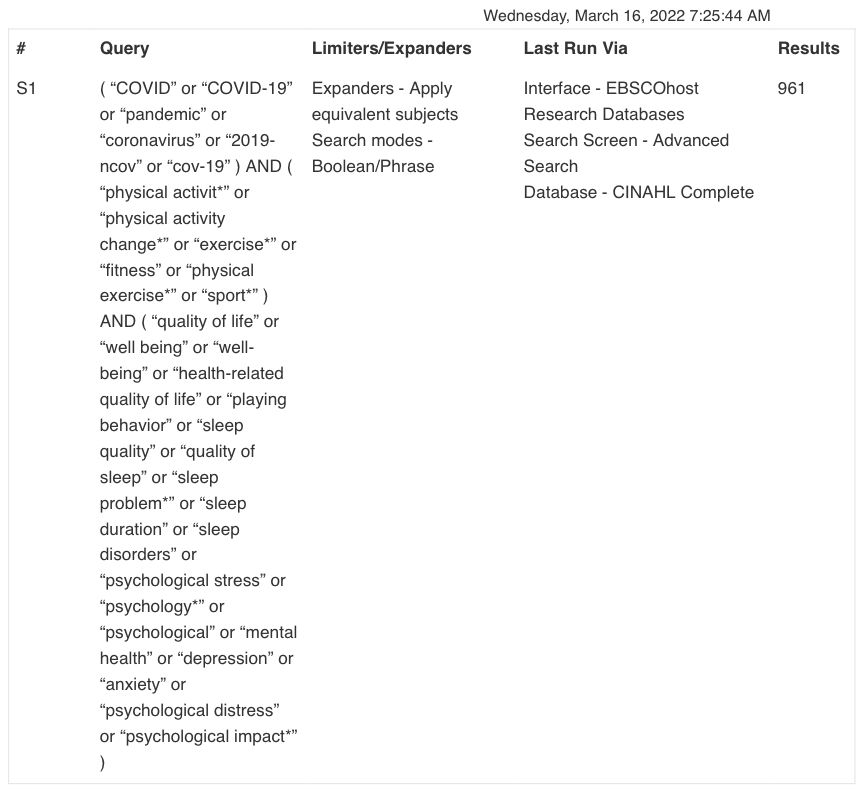 |
| --- |
| Cochrane Library  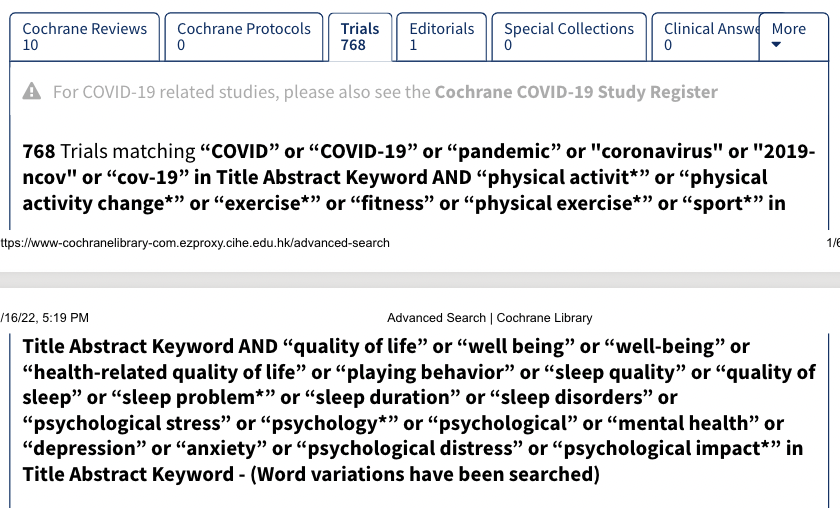 |
| Embase  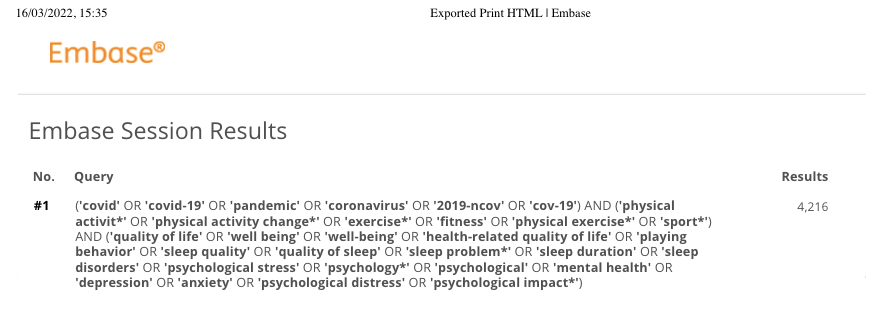 |
| Medline  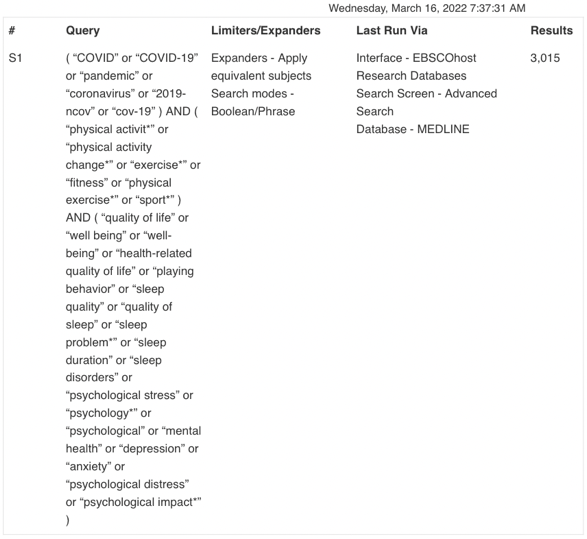 |
| PsycINFO  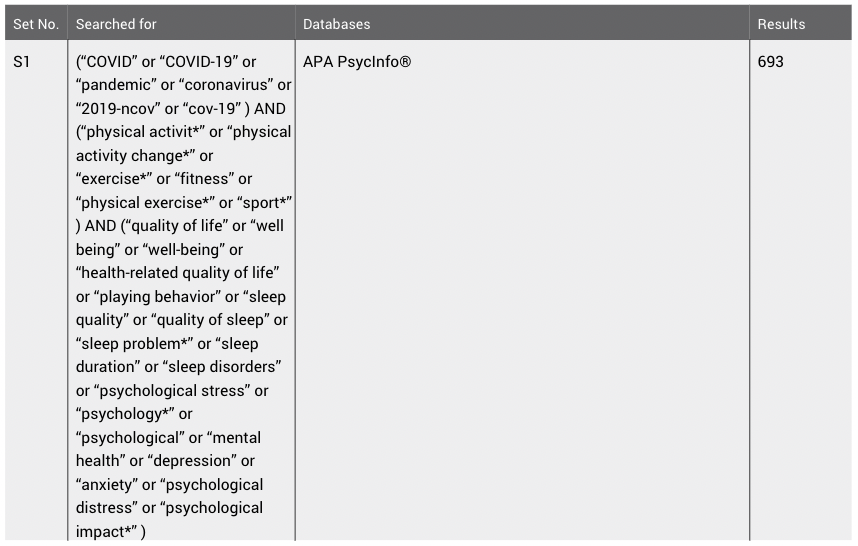 |
| PubMed  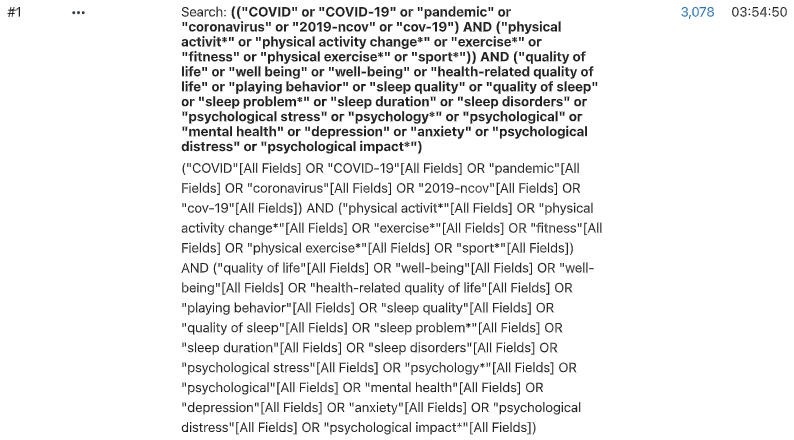 |

Supplementary Table 2. The search terms used in lectures search.

| **Concepts** | **Search terms** |
| --- | --- |
| COVID-19 | “COVID” or “COVID-19” or “pandemic” or “coronavirus” or “2019-ncov” or “cov-19” |
| Physical activity changes | “physical activit*” or “physical activity change*” or “exercise*” or “fitness” or “physical exercise*” or “sport*” |
| Impacts | “quality of life” or “well being” or “well-being” or “health-related quality of life” or “playing behavior” or “sleep quality” or “quality of sleep” or “sleep problem*” or “sleep duration” or “sleep disorders” or “psychological stress” or “psychology*” or “psychological” or “mental health” or “depression” or “anxiety” or “psychological distress” or “psychological impact*” |

# Supplementary Table 3. Reasons for exclusion.

| Reasons | Authors |
| --- | --- |
| Insufficient data available (N=53) | [1-53] |
| Not targeted population (N=95) | [54-148] |
| Editorial (N=53) | [149-201] |
| Review (N=14) | [202-215] |
| Qualitative studies (N=2) | [216, 217] |
| Validation (N=1) | [218] |
| Abstract (N=6) | [219-224] |
| Conference (N=2) | [225, 226] |
| Dissertation (N=1) | [227] |

1. Abid R, Ammar A, Maaloul R et al. Effect of COVID-19-related home confinement on sleep quality, screen time and physical activity in tunisian boys and girls: A survey. *International Journal of Environmental Research and Public Health*. 2021;18(6):1-12.

2. Aguilar-Farias N, Toledo-Vargas M, Miranda-Marquez S et al. Sociodemographic Predictors of Changes in Physical Activity, Screen Time, and Sleep among Toddlers and Preschoolers in Chile during the COVID-19 Pandemic. *International journal of environmental research and public health*. 2020;18(1).

3. Bahatheg RO. Young Children's Nutrition During the COVID-19 Pandemic Lockdown: A Comparative Study. *Early childhood education journal*. 2021:1-9.

4. Begum A, Sangeetha S, Sridevi G. Awareness of stress among children during lockdown-a survey. *International Journal of Pharmaceutical Research*. 2020;12:2082-92.

5. Bösselmann V, Amatriain-Fernández S, Gronwald T et al. Physical Activity, Boredom and Fear of COVID-19 Among Adolescents in Germany. *Frontiers in psychology*. 2021;12:624206.

6. Brent Jackson S, Stevenson KT, Larson LR et al. Outdoor activity participation improves adolescents’ mental health and well-being during the covid-19 pandemic. *International Journal of Environmental Research and Public Health*. 2021;18(5):1-19.

7. Bringolf-Isler B, Hänggi J, Kayser B et al. COVID-19 pandemic and health related quality of life in primary school children in Switzerland: a repeated cross-sectional study. *Swiss medical weekly*. 2021;151:w30071.

8. Burdzovic Andreas J, Brunborg GS. Self-reported Mental and Physical Health among Norwegian Adolescents before and during the COVID-19 Pandemic. *JAMA Network Open*. 2021.

9. Chaturvedi K, Vishwakarma DK, Singh N. COVID-19 and its impact on education, social life and mental health of students: A survey. *Children & Youth Services Review*. 2021;121:N.PAG-N.PAG.

10. Chen Y, Osika W, Henriksson G et al. Impact of COVID-19 pandemic on mental health and health behaviors in Swedish adolescents. *Scandinavian journal of public health*. 2022;50(1):26-32.

11. Hamoda HM, Chiumento A, Alonge O et al. Addressing the consequences of the covid-19 lockdown for children's mental health: Investing in school mental health programs. *Psychiatric Services*. 2021;72(6):729-31.

12. Ijaz S, Ismail S, Fayyaz L et al. Physical Activity, Screen Time and Emotional Wellbeing of Population during COVID-19 Pandemic in Pakistan. *Biomedica*. 2020;36(4).

13. Ijaz SF, Ijaz I. Impact of excessive screen use on sleep habits in children during covid-19 pandemic. *Pakistan Paediatric Journal*. 2021;45(4):395-400.

14. Ishimoto Y, Yamane T, Matsumoto Y et al. The impact of gender differences, school adjustment, social interactions, and social activities on emotional and behavioral reactions to the COVID-19 pandemic among Japanese school children. *SSM Ment Health*. 2022;2:100077.

15. Jáuregui A, Argumedo G, Medina C et al. Factors associated with changes in movement behaviors in toddlers and preschoolers during the COVID-19 pandemic: A national cross-sectional study in Mexico. *Preventive Medicine Reports*. 2021;24.

16. Kang S, Sun Y, Zhang X et al. Is physical activity associated with mental health among chinese adolescents during isolation in COVID-19 pandemic? *Journal of Epidemiology and Global Health*. 2021;11(1):26-33.

17. Kerekes N, Bador K, Sfendla A et al. Changes in adolescents’ psychosocial functioning and well-being as a consequence of long-term covid-19 restrictions. *International Journal of Environmental Research and Public Health*. 2021;18(16).

18. Kiss O, Alzueta E, Yuksel D et al. The Pandemic's Toll on Young Adolescents: Prevention and Intervention Targets to Preserve Their Mental Health. *Journal of Adolescent Health*. 2022;70(3):387-95.

19. Kuhn AP, Kowalski AJ, Wang Y et al. On the move or barely moving? Age-related changes in physical activity, sedentary, and sleep behaviors by weekday/weekend following pandemic control policies. *International Journal of Environmental Research and Public Health*. 2022;19(1).

20. Lange SJ, Kompaniyets L, Freedman DS et al. Longitudinal Trends in Body Mass Index Before and During the COVID-19 Pandemic Among Persons Aged 2-19 Years - United States, 2018-2020. *MMWR Morbidity and mortality weekly report*. 2021;70(37):1278-83.

21. Lee SJ, Ward KP, Chang OD et al. Parenting activities and the transition to home-based education during the COVID-19 pandemic. *Children & Youth Services Review*. 2021;122:N.PAG-N.PAG.

22. Lessard LM, Puhl RM. Adolescents' Exposure to and Experiences of Weight Stigma During the COVID-19 Pandemic. *Journal of pediatric psychology*. 2021;46(8):950-9.

23. Liao S, Luo B, Liu H et al. Bilateral associations between sleep duration and depressive symptoms among Chinese adolescents before and during the COVID-19 pandemic. *Sleep Medicine*. 2021;84:289-93.

24. Lim MTC, Ramamurthy MB, Aishworiya R et al. School closure during the coronavirus disease 2019 (COVID-19) pandemic – Impact on children's sleep. *Sleep Medicine*. 2021;78:108-14.

25. López-Bueno R, López-Sánchez GF, Casajús JA et al. Health-Related Behaviors Among School-Aged Children and Adolescents During the Spanish Covid-19 Confinement. *Frontiers in Pediatrics*. 2020;8.

26. López-Gil JF, Tremblay MS, Brazo-Sayavera J. Changes in Healthy Behaviors and Meeting 24-h Movement Guidelines in Spanish and Brazilian Preschoolers, Children and Adolescents during the COVID-19 Lockdown. *Children*. 2021;8(2):1-10.

27. Lourenço A, Martins F, Pereira B et al. Children are back to school, but is play still in lockdown? Play experiences, social interactions, and children’s quality of life in primary education in the covid-19 pandemic in 2020. *International Journal of Environmental Research and Public Health*. 2021;18(23).

28. McCluskey G, Fry D, Hamilton S et al. School closures, exam cancellations and isolation: the impact of Covid-19 on young people’s mental health. *Emotional and Behavioural Difficulties*. 2021;26(1):46-59.

29. Miller RL, Moran M, Shomaker LB et al. Health effects of COVID-19 for vulnerable adolescents in a randomized controlled trial. *School Psychology*. 2021;36(5):293-302.

30. Min S-K, Son W-H, Choi B-H et al. Psychophysical condition of adolescents in coronavirus disease 2019. *Journal of exercise rehabilitation*. 2021;17(2):112-9.

31. Morres ID, Galanis E, Hatzigeorgiadis A et al. Physical activity, sedentariness, eating behaviour and well-being during a covid-19 lockdown period in greek adolescents. *Nutrients*. 2021;13(5).

32. Mzadi AE, Zouini B, Kerekes N et al. Mental Health Profiles in a Sample of Moroccan High School Students: Comparison Before and During the COVID-19 Pandemic. *Front Psychiatry*. 2021;12:752539.

33. Nakajima R, Kamada H, Kasai T et al. Effect of temporary school closure due to COVID-19 on musculoskeletal function in elementary school children. *Journal of rural medicine : JRM*. 2021;16(3):154-9.

34. Peralta GP, Camerini AL, Haile SR et al. Lifestyle behaviours of children and adolescents during the first two waves of the COVID-19 pandemic in Switzerland and their relation to well-being: a population-based study. 2021.

35. Pombo A, Luz C, Rodrigues LP et al. Correlates of children's physical activity during the COVID-19 confinement in Portugal. *Public health*. 2020;189:14-9.

36. Ramos-Álvarez O, Arufe-Giráldez V, Cantarero-Prieto D et al. Impact of SARS-CoV-2 lockdown on anthropometric parameters in children 11/12 years old. *Nutrients*. 2021;13(11).

37. Ren H, He X, Bian X et al. The Protective Roles of Exercise and Maintenance of Daily Living Routines for Chinese Adolescents During the COVID-19 Quarantine Period. *Journal of Adolescent Health*. 2021;68(1):35-42.

38. Saito M, Kikuchi Y, Lefor AK et al. Mental health in Japanese children during school closures due to the COVID-19. *Pediatrics International*. 2021.

39. Štveráková T, Jačisko J, Busch A et al. The impact of COVID-19 on physical activity of Czech children. *PLoS ONE*. 2021;16(7):14.

40. Suhua X, Zi Y, Li Z. Physical Activity, Screen Time, and Mood Disturbance Among Chinese Adolescents During COVID-19. *Journal of Psychosocial Nursing & Mental Health Services*. 2021;59(4):14-20.

41. Surekha BC, Karanati K, Venkatesan K et al. E-Learning During COVID-19 Pandemic: A Surge in Childhood Obesity. *Indian journal of otolaryngology and head and neck surgery : official publication of the Association of Otolaryngologists of India*. 2021:1-7.

42. Szpunar G, Cannoni E, Di Norcia A. La didattica a distanza durante il lockdown in Italia: il punto di vista delle famiglie. *Journal of Educational, Cultural and Psychological Studies*. 2021;23:137-55.

43. Tardif-Grenier K, Archambault I, Dupéré V et al. Canadian Adolescents’ Internalized Symptoms in Pandemic Times: Association with Sociodemographic Characteristics, Confinement Habits, and Support. *Psychiatric Quarterly*. 2021;92(3):1309-25.

44. Toppe T, Stengelin R, Schmidt LS et al. Explaining Variation in Parents' and Their Children's Stress During COVID-19 Lockdowns. *Frontiers in psychology*. 2021;12:645266.

45. Tso WWY, Wong RS, Tung KTS et al. Vulnerability and resilience in children during the covid-19 pandemic. *European Child & Adolescent Psychiatry*. 2020.

46. Tulchin-Francis K, Stevens W, Gu X et al. The impact of the coronavirus disease 2019 pandemic on physical activity in U.S. children. *Journal of Sport and Health Science*. 2021;10(3):323-32.

47. Wahl-Alexander Z, Camic CL. Impact of COVID-19 on School-Aged Male and Female Health-Related Fitness Markers. *Pediatric exercise science*. 2021;33(2):61-4.

48. Wang MT, Scanlon CL, Hua M et al. Social Distancing and Adolescent Psychological Well-Being: The Role of Practical Knowledge and Exercise. *Academic Pediatrics*. 2021.

49. Wang P, Sun X, Li W et al. Mental Health of Parents and Preschool-Aged Children During the COVID-19 Pandemic: The Mediating Role of Harsh Parenting and Child Sleep Disturbances. *Frontiers in Psychiatry*. 2021;12.

50. Wright LJ, Williams SE, Veldhuijzen van Zanten J. Physical Activity Protects Against the Negative Impact of Coronavirus Fear on Adolescent Mental Health and Well-Being During the COVID-19 Pandemic. *Front Psychol*. 2021;12:580511.

51. Wright LJ, Williams SE, Veldhuijzen van Zanten JJCS. Physical Activity Protects Against the Negative Impact of Coronavirus Fear on Adolescent Mental Health and Well-Being During the COVID-19 Pandemic. *Frontiers in psychology*. 2021;12:580511.

52. Wunsch K, Nigg C, Niessner C et al. The Impact of COVID-19 on the Interrelation of Physical Activity, Screen Time and Health-Related Quality of Life in Children and Adolescents in Germany: Results of the Motorik-Modul Study. *Children*. 2021;8(2):1-14.

53. Xiao S, Yan Z, Zhao L. Physical Activity, Screen Time, and Mood Disturbance Among Chinese Adolescents During COVID-19. *Journal of psychosocial nursing and mental health services*. 2021;59(4):14-20.

54. The Impact of COVID-19 on the Well-Being of Division III Student-Athletes. *Sport Journal*. 2020:N.PAG-N.PAG.

55. The Impact of COVID-19 Pandemic on High Performance Secondary School Student-Athletes. *Sport Journal*. 2020:N.PAG-N.PAG.

56. COVID-19 and its impact on student-athlete depression and anxiety: the return to campus. *Sport Journal*. 2021:N.PAG-N.PAG.

57. Al Agha AE, Alharbi RS, Almohammadi OA et al. Impact of COVID-19 lockdown on glycemic control in children and adolescents. *Saudi medical journal*. 2021;42(1):44-8.

58. Alves JM, Yunker AG, DeFendis A et al. Prenatal exposure to gestational diabetes is associated with anxiety and physical inactivity in children during COVID-19. *Clinical Obesity*. 2021;11(1).

59. Anbarasu A, Bhuvaneswari M. COVID-19 pandemic and psychosocial problems in children and adolescents in vellore-district. *European Journal of Molecular and Clinical Medicine*. 2020;7(7):334-9.

60. Batalla-Gavalda A, Cecilia-Gallego P, Revillas-Ortega F et al. Variations in the mood states during the different phases of COVID-19’s lockdown in young athletes. *International Journal of Environmental Research and Public Health*. 2021;18(17).

61. Berasategi Sancho N, Idoiaga Mondragon N, Dosil Santamaria M et al. The Well-being of children in lock-down: Physical, emotional, social and academic impact. *Children & Youth Services Review*. 2021;127:N.PAG-N.PAG.

62. Biyik KS, Özal C, Tunçdemir M et al. The functional health status of children with cerebral palsy during the COVID-19 pandemic stay-at-home period: A parental perspective. *Turkish Journal of Pediatrics*. 2021;63(2):223-36.

63. Boukrim M, Obtel M, Kasouati J et al. COVID-19 and confinement: Effect on weight load, physical activity and eating behavior of higher education students in southern Morocco. *Annals of Global Health*. 2021;87(1):1-11.

64. Bucak IH, Almis H, Tasar SO et al. Have the sleep habits in children of health workers been more affected during the COVID-19 pandemic? *Sleep medicine*. 2021;83:235-40.

65. Bulut NS, Yorguner N, Akvardar Y. Impact of covid-19 on the life of higher-education students in İstanbul: Relationship between social support, health-risk behaviors, and mental/academic well-being. *Anadolu Psikiyatri Dergisi*. 2021;22(6):291-300.

66. Carroll N, Sadowski A, Laila A et al. The Impact of COVID-19 on Health Behavior, Stress, Financial and Food Security among Middle to High Income Canadian Families with Young Children. *Nutrients*. 2020;12(8).

67. Chen J, Sang G, Zhang Y et al. Intervention effect of the integration model on negative emotions of adolescents during the outbreak of Corona Virus Disease 2019. *Psychiatria Danubina*. 2021;33(1):86-94.

68. Chen X, Qi H, Liu R et al. Depression, anxiety and associated factors among Chinese adolescents during the COVID-19 outbreak: a comparison of two cross-sectional studies. *Translational Psychiatry*. 2021;11(1).

69. Cipolla C, Curatola A, Ferretti S et al. Eating habits and lifestyle in children with obesity during the covid19 lockdown: A survey in an italian center. *Acta Biomedica*. 2021;92(2).

70. Commodari E, La Rosa VL. Adolescents in Quarantine During COVID-19 Pandemic in Italy: Perceived Health Risk, Beliefs, Psychological Experiences and Expectations for the Future. *Frontiers in psychology*. 2020;11:559951.

71. Crescentini C, Feruglio S, Matiz A et al. Stuck Outside and Inside: An Exploratory Study on the Effects of the COVID-19 Outbreak on Italian Parents and Children's Internalizing Symptoms. *Frontiers in psychology*. 2020;11:586074.

72. Denerel N, Köyağasıoglu O, Şenışık S et al. Effects of Long-Duration Home Isolation Linked to the COVID-19 Pandemic on Mental Health of Adolescent Athletes. *Pediatric Exercise Science*. 2021;33(4):170-6.

73. Di Cagno A, Buonsenso A, Baralla F et al. Psychological impact of the quarantine-induced stress during the coronavirus (COVID-19) outbreak among Italian athletes. *International Journal of Environmental Research and Public Health*. 2020;17(23):1-13.

74. Ding X, Yao J. Peer Education Intervention on Adolescents' Anxiety, Depression, and Sleep Disorder during the COVID-19 Pandemic. *Psychiatria Danubina*. 2020;32(3-4):527-35.

75. DiSabella M, Pierce EL, Ratnaseelan A et al. Pediatric headache experience during the COVID-19 pandemic. *Headache*. 2021;61(SUPPL 1):166.

76. Dun Y, Ripley-Gonzalez JW, Zhou N et al. The association between prior physical fitness and depression in young adults during the COVID-19 pandemic-a crosssectional, retrospective study. *PeerJ*. 2021;9.

77. Elbarbary NS, dos Santos TJ, de Beaufort C et al. The Challenges of Managing Pediatric Diabetes and Other Endocrine Disorders During the COVID-19 Pandemic: Results From an International Cross-Sectional Electronic Survey. *Frontiers in Endocrinology*. 2021;12.

78. Elliott S, Drummond MJ, Prichard I et al. Understanding the impact of COVID-19 on youth sport in Australia and consequences for future participation and retention. *BMC public health*. 2021;21(1):448.

79. Engels ES, Mutz M, Demetriou Y et al. Levels of physical activity in four domains and affective wellbeing before and during the Covid-19 pandemic. *Archives of Public Health*. 2021;79(1):1-9.

80. Esteves CS, de Oliveira CR, Argimon IIdL. Social Distancing: Prevalence of Depressive, Anxiety, and Stress Symptoms Among Brazilian Students During the COVID-19 Pandemic. *Frontiers in public health*. 2021;8:589966.

81. Faraji M, Sepahvand E, Rahmati R. The effects of covid-19 related quarantine on physical and social pain of children with autism. *Journal of Pain Management*. 2021;14(1):81-4.

82. Farì G, Di Paolo S, Ungaro D et al. The Impact of COVID-19 on Sport and Daily Activities in an Italian Cohort of Football School Children. *International Journal of Athletic Therapy & Training*. 2021;26(5):274-8.

83. Feng Z, Xu L, Cheng P et al. The psychological impact of COVID-19 on the families of first-line rescuers. *Indian journal of psychiatry*. 2020;62(Suppl 3):S438-S44.

84. Fernández Cruz M, Álvarez Rodríguez J, Ávalos Ruiz I et al. Evaluation of the Emotional and Cognitive Regulation of Young People in a Lockdown Situation Due to the Covid-19 Pandemic. *Frontiers in psychology*. 2020;11:565503.

85. Fernández RS, Crivelli L, Guimet NM et al. Psychological distress associated with COVID-19 quarantine: Latent profile analysis, outcome prediction and mediation analysis. *Journal of Affective Disorders*. 2020;277:75-84.

86. Ferrante MJ, Goldsmith J, Tauriello S et al. Food acquisition and daily life for U.S. families with 4-to 8-year-old children during COVID-19: Findings from a nationally representative survey. *International Journal of Environmental Research and Public Health*. 2021;18(4):1-15.

87. Foley S, Badinlou F, Brocki KC et al. Family function and child adjustment difficulties in the covid-19 pandemic: An international study. *International Journal of Environmental Research and Public Health*. 2021;18(21).

88. Gjaka M, Feka K, Bianco A et al. The effect of covid-19 lockdown measures on physical activity levels and sedentary behaviour in a relatively young population living in kosovo. *Journal of Clinical Medicine*. 2021;10(4):1-15.

89. Gruba G, Kasiak PS, Gębarowska J et al. Pals study of sleep deprivation and mental health consequences of the covid-19 pandemic among university students: A cross-sectional survey. *International Journal of Environmental Research and Public Health*. 2021;18(18).

90. Hagen K, Solem S, Stavrum AK et al. Mental health symptoms during the first months of the COVID-19 outbreak in Norway: A cross-sectional survey study. *Scandinavian journal of public health*. 2021:14034948211059525.

91. Hosen I, Al Mamun F, Sikder MT et al. Prevalence and associated factors of problematic smartphone use during the covid-19 pandemic: A bangladeshi study. *Risk Management and Healthcare Policy*. 2021;14:3797-805.

92. José Puccinelli P, Santos da Costa T, Seffrin A et al. Reduced level of physical activity during COVID-19 pandemic is associated with depression and anxiety levels: an internet-based survey. *BMC Public Health*. 2021;21(1):1-11.

93. Kalvin CB, Jordan RP, Rowley SN et al. Conducting CBT for Anxiety in Children with Autism Spectrum Disorder During COVID-19 Pandemic. *Journal of Autism and Developmental Disorders*. 2021;51(11):4239-47.

94. Kuygun Karci C, Arici Gurbuz A. Challenges of children and adolescents with attention-deficit/hyperactivity disorder during the covid-19 pandemic. *Nordic Journal of Psychiatry*. 2021.

95. Langer A, Gassner L, Flotz A et al. How COVID-19 will boost remote exercise-based treatment in Parkinson’s disease: a narrative review. *npj Parkinson's Disease*. 2021;7(1).

96. Lee S-M, So W-Y, Youn H-S. Importance-Performance Analysis of Health Perception among Korean Adolescents during the COVID-19 Pandemic. *International journal of environmental research and public health*. 2021;18(3).

97. Li Y, Zhao J, Ma Z et al. Mental Health Among College Students During the COVID-19 Pandemic in China: A 2-Wave Longitudinal Survey. *Journal of Affective Disorders*. 2021;281:597-604.

98. Li Z-L, Liu R, He F et al. Prevalence of Internet Addiction Disorder and Its Correlates Among Clinically Stable Adolescents With Psychiatric Disorders in China During the COVID-19 Outbreak. *Frontiers in psychiatry*. 2021;12:686177.

99. Lin J, Guo T, Becker B et al. Depression is Associated with Moderate-Intensity Physical Activity Among College Students During the COVID-19 Pandemic: Differs by Activity Level, Gender and Gender Role. *Psychology research and behavior management*. 2020;13:1123-34.

100. Lindoso L, Astley C, Queiroz LB et al. Physical and mental health impacts during COVID-19 quarantine in adolescents with preexisting chronic immunocompromised conditions. *J Pediatr (Rio J)*. 2021.

101. Logan LM, Ciftci B, Longoni G et al. Effects of COVID-19 on Mental Health, Physical Activity and Sleep in Pediatric Neuroinflammatory Disorders. *Multiple Sclerosis Journal*. 2021;27(1 SUPPL):117-8.

102. Logan LM, Kavaklioglu BC, Longoni G et al. Impact of the COVID-19 pandemic on mental health, physical activity and sleep in children with neuroinflammatory disorders. *Neurology*. 2021;96(15 SUPPL 1).

103. López-Aymes G, Valadez MdLD, Rodríguez-Naveiras E et al. A Mixed Methods Research Study of Parental Perception of Physical Activity and Quality of Life of Children Under Home Lock Down in the COVID-19 Pandemic. *Frontiers in psychology*. 2021;12:649481.

104. Luo L, Song N, Yang H et al. Intervention Effect of Long-Term Aerobic Training on Anxiety, Depression, and Sleep Quality of Middle School Students With Depression After COVID-19. *Frontiers in Psychiatry*. 2021;12.

105. Mc Guine T, Biese K, Hetzel S et al. The impact of covid-19 related school closures and sport cancellations on the health of adolescent athletes. *Orthopaedic Journal of Sports Medicine*. 2021;9(7 SUPPL 3).

106. McCormack GR, Doyle-Baker PK, Petersen JA et al. Parent anxiety and perceptions of their child's physical activity and sedentary behaviour during the COVID-19 pandemic in Canada. *Preventive Medicine Reports*. 2020;20.

107. McDonnell T, Barrett M, McNicholas F et al. Increased mental health presentations by children aged 5-15 at emergency departments during the first 12 months of covid-19. *Irish Medical Journal*. 2021;114(5).

108. McGuine T, Biese K, Hetzel S et al. THE IMPACT OF COVID-19 RELATED SCHOOL CLOSURES AND SPORT CANCELLATIONS ON THE HEALTH OF ADOLESCENT ATHLETES...Pediatric Research in Sports Medicine (PRiSM), 8th Annual Meeting, 28-30 January, 2021. *Orthopaedic Journal of Sports Medicine*. 2021;19:306-.

109. McGuine TA, Biese KM, Petrovska L et al. Changes in the Health of Adolescent Athletes: A Comparison of Health Measures Collected Before and During the COVID-19 Pandemic. *Journal of athletic training*. 2021;56(8):836-44.

110. McGuine TA, K MB, Hetzel SJ et al. High School Sports During the COVID-19 Pandemic: The Effect of Sport Participation on the Health of Adolescents. *J Athl Train*. 2022;57(1):51-8.

111. Mohammadzadeh F, Delshad Noghabi A, Khosravan S et al. Anxiety Severity Levels and Coping Strategies during the COVID-19 Pandemic among People Aged 15 Years and Above in Gonabad, Iran. *Archives of Iranian medicine*. 2020;23(9):633-8.

112. Moore SA, Sharma R, Martin Ginis KA et al. Parental Support Is Associated With Healthy Movement Behaviours In Children With Disabilities During Covid-19. *Medicine & Science in Sports & Exercise*. 2021;53:222-.

113. Morales J, Fukuda DH, Garcia V et al. Behavioural improvements in children with autism spectrum disorder after participation in an adapted judo programme followed by deleterious effects during the COVID-19 lockdown. *International Journal of Environmental Research and Public Health*. 2021;18(16).

114. Munasinghe S, Sperandei S, Freebairn L et al. The Impact of Physical Distancing Policies During the COVID-19 Pandemic on Health and Well-Being Among Australian Adolescents. *Journal of Adolescent Health*. 2020;67(5):653-61.

115. Muñoz-Fernández N, Rodríguez-Meirinhos A. Adolescents’ concerns, routines, peer activities, frustration, and optimism in the time of covid-19 confinement in spain. *Journal of Clinical Medicine*. 2021;10(4):1-13.

116. Nct. Physical Training and Diet for Childhood Obesity. [*https://clinicaltrialsgov/show/NCT04789525*](https://clinicaltrialsgov/show/NCT04789525). 2021.

117. Neshteruk CD, Zizzi A, Suarez L et al. Weight-Related Behaviors of Children with Obesity during the COVID-19 Pandemic. *Childhood obesity (Print)*. 2021;17(6):371‐8.

118. Nie Y, Ma Y, Li X et al. PA during the COVID-19 outbreak in China: a cross-sectional study. *Neural computing & applications*. 2021:1-16.

119. O'Donoghue B, Castagnini E, Langstone A et al. Sedentary behaviour in young people presenting with a first episode of psychosis before and during the covid-19 pandemic restrictions. *Schizophrenia Research*. 2021;233:31-3.

120. O’rourke T, Dale R, Humer E et al. Health Behaviors in Austrian Apprentices and School Students during the COVID-19 Pandemic. *International Journal of Environmental Research and Public Health*. 2022;19(3).

121. Pietrabissa G, Volpi C, Bottacchi M et al. The impact of social isolation during the covid-19 pandemic on physical and mental health: The lived experience of adolescents with obesity and their caregivers. *International Journal of Environmental Research and Public Health*. 2021;18(6):1-20.

122. Pietrobelli A, Pecoraro L, Ferruzzi A et al. Effects of COVID-19 Lockdown on Lifestyle Behaviors in Children with Obesity Living in Verona, Italy: A Longitudinal Study. *Obesity*. 2020;28(8):1382-5.

123. Pigaiani Y, Zoccante L, Zocca A et al. Adolescent Lifestyle Behaviors, Coping Strategies and Subjective Wellbeing during the COVID-19 Pandemic: An Online Student Survey. *Healthcare (Basel, Switzerland)*. 2020;8(4).

124. Pigeaud L, de Veld L, van Hoof J et al. Acute Alcohol Intoxication in Dutch Adolescents Before, During, and After the First COVID-19 Lockdown. *J Adolesc Health*. 2021;69(6):905-9.

125. Piña A, Mirhajianmoghadam H, Ostrin LA. Objective and subjective behavioral measures in myopic and non-myopic children during the COVID-19 pandemic. *Investigative Ophthalmology and Visual Science*. 2021;62(8).

126. Przybylski R, Craig M, Lippmann M et al. Activity During the COVID-19 Pandemic in Children with Cardiac Rhythm Management Devices. *Pediatric Cardiology*. 2021.

127. Qi H, Liu R, Chen X et al. Prevalence of anxiety and associated factors for Chinese adolescents during the COVID-19 outbreak. *Psychiatry and clinical neurosciences*. 2020;74(10):555-7.

128. Sadeghipour HR, Zar A, Pakizeh A et al. Evaluation of health-related quality of life in physically active and physically inactive students during the COVID-19 pandemic in Iran. *Cities (London, England)*. 2021;118:103367.

129. Sarker T, Sarkar A, Rabbany MG et al. Evaluation of preventive, supportive and awareness building measures among international students in China in response to COVID-19: a structural equation modeling approach. *Global health research and policy*. 2021;6(1):10.

130. Schlichtiger J, Brunner S, Steffen J et al. Mental health impairment triggered by the COVID-19 pandemic in a sample population of German students. *Journal of Investigative Medicine*. 2020;68(8):1394-6.

131. Schmits E, Dekeyser S, Klein O et al. Psychological distress among students in higher education: One year after the beginning of the COVID-19 pandemic. *International Journal of Environmental Research and Public Health*. 2021;18(14).

132. Sciberras E, Patel P, Stokes MA et al. Physical Health, Media Use, and Mental Health in Children and Adolescents With ADHD During the COVID-19 Pandemic in Australia. *Journal of attention disorders*. 2022;26(4):549-62.

133. Shah N, Karguppikar M, Bhor S et al. Impact of lockdown for COVID-19 pandemic in Indian children and youth with type 1 diabetes from different socio-economic classes. *Journal of pediatric endocrinology & metabolism : JPEM*. 2020;34(2):217-23.

134. Sharpe D, Rajabi M, Chileshe C et al. Mental health and wellbeing implications of the COVID-19 quarantine for disabled and disadvantaged children and young people: evidence from a cross-cultural study in Zambia and Sierra Leone. *BMC psychology*. 2021;9(1):79.

135. Shaun MMA, Nizum MWR, Munny S et al. Eating habits and lifestyle changes among higher studies students post-lockdown in Bangladesh: A web-based cross-sectional study. *Heliyon*. 2021;7(8):e07843.

136. Shepherd HA, Evans T, Gupta S et al. High school student-athletes' experiences with the COVID-19 pandemic: The impact on their physical activity engagement and mental health. *Clinical Journal of Sport Medicine*. 2021;31(3):e126.

137. Shepherd HA, Evans T, Gupta S et al. The impact of COVID-19 on high school student-athlete experiences with physical activity, mental health, and social connection. *International Journal of Environmental Research and Public Health*. 2021;18(7).

138. Sutter EN, Francis LS, Francis SM et al. Disrupted Access to Therapies and Impact on Well-Being During the COVID-19 Pandemic for Children With Motor Impairment and Their Caregivers. *American journal of physical medicine & rehabilitation*. 2021;100(9):821-30.

139. Thomas J, Bowes N, Meyers R et al. Mental well-being and physical activity of young people experiencing homelessness before and during COVID-19 lockdown: A longitudinal study. *Mental Health and Physical Activity*. 2021;21.

140. Valadez MdLD, Rodríguez-Naveiras E, Castellanos-Simons D et al. Physical Activity and Well-Being of High Ability Students and Community Samples During the COVID-19 Health Alert. *Frontiers in psychology*. 2020;11:606167.

141. Valenzise M, D'Amico F, Cucinotta U et al. The lockdown effects on a pediatric obese population in the COVID-19 era. *Italian journal of pediatrics*. 2021;47(1):209.

142. Vall-Roqué H, Andrés A, Saldaña C. The impact of COVID-19 lockdown on social network sites use, body image disturbances and self-esteem among adolescent and young women. *Progress in Neuro-Psychopharmacology and Biological Psychiatry*. 2021;110.

143. Welling MS, Abawi O, Van Den Eynde E et al. Impact of COVID-19 lockdown measures on lifestyle behavior in children and adolescents with severe obesity. *Obesity Facts*. 2021;14(SUPPL 1):46.

144. Welling MS, Abawi O, Van Den Eynde E et al. Impact of the COVID-19 pandemic and related lockdown measures on lifestyle behaviors and wellbeing in children and adolescents with severe obesity. *Obesity Facts*. 2021.

145. Yuan YQ, Ding JN, Bi N et al. Physical activity and sedentary behaviour among children and adolescents with intellectual disabilities during the covid‐19 lockdown in china. *Journal of Intellectual Disability Research*. 2021.

146. Zhai X, Zeng J, Eshak ES et al. The influencing factors of sleep quality among Chinese junior and senior high school adolescents during the COVID-19 pandemic. *Journal of Tropical Pediatrics*. 2021;67(4).

147. Zhang J, Zhou Z, Zhang W. Intervention effect of research-based psychological counseling on adolescents mental health during the covid-19 epidemic. *Psychiatria Danubina*. 2021;33(2):209-16.

148. Zhou J, Yuan X, Qi H et al. Prevalence of depression and its correlative factors among female adolescents in China during the coronavirus disease 2019 outbreak. *Globalization and Health*. 2020;16(1).

149. Amjadi K. Exploring Factors That Influence Children’s Growth and Development During a Pandemic. *Global Pediatric Health*. 2021;8.

150. Bates LC, Zieff G, Stanford K et al. COVID-19 Impact on Behaviors across the 24-Hour Day in Children and Adolescents: Physical Activity, Sedentary Behavior, and Sleep. *Children (Basel, Switzerland)*. 2020;7(9).

151. Browne NT, Snethen JA, Greenberg CS et al. When Pandemics Collide: The Impact of COVID-19 on Childhood Obesity. *Journal of Pediatric Nursing*. 2021;56:90-8.

152. Chen F, Zheng D, Liu J et al. Depression and anxiety among adolescents during COVID-19: A cross-sectional study. *Brain, Behavior, and Immunity*. 2020;88:36-8.

153. Christakis DA. Pediatrics and COVID-19. *JAMA - Journal of the American Medical Association*. 2020;324(12):1147-8.

154. Courtney D, Watson P, Battaglia M et al. COVID-19 Impacts on Child and Youth Anxiety and Depression: Challenges and Opportunities. *Canadian Journal of Psychiatry*. 2020;65(10):688-91.

155. Cuschieri S, Grech S. COVID-19: a one-way ticket to a global childhood obesity crisis? *Journal of Diabetes and Metabolic Disorders*. 2020;19(2):2027-30.

156. de Lannoy L, Rhodes RE, Moore SA et al. Regional differences in access to the outdoors and outdoor play of Canadian children and youth during the COVID-19 outbreak. *Canadian journal of public health = Revue canadienne de sante publique*. 2020;111(6):988-94.

157. Efuribe C, Barre-Hemingway M, Vaghefi E et al. Coping with the COVID-19 crisis: A call for youth engagement and the inclusion of young people in matters that affect their lives. *Journal of Adolescent Health*. 2020;67(1):16-7.

158. Faigenbaum AD, MacDonald JP, Carvalho C et al. THE PEDIATRIC INACTIVITY TRIAD: A TRIPLE JEOPARDY FOR MODERN DAY YOUTH. *ACSM's Health & Fitness Journal*. 2020;24(4):10-7.

159. Gabriel MG, Brown A, León M et al. Power and social control of youth during the covid-19 pandemic. *Leisure Sciences*. 2020.

160. Ghosh R, Dubey MJ, Chatterjee S et al. Impact of COVID-19 on children: Special focus on the psychosocial aspect. *Minerva Pediatrica*. 2020;72(3):226-35.

161. Golberstein E, Wen H, Miller BF. Coronavirus Disease 2019 and Effects of School Closure for Children and Their Families-Reply. *JAMA Pediatrics*. 2021;175(2):211-2.

162. Guan H, Okely AD, Aguilar-Farias N et al. Promoting healthy movement behaviours among children during the COVID-19 pandemic. *The Lancet Child and Adolescent Health*. 2020;4(6):416-8.

163. Gupta S, Schreiber M, McGuire T et al. Addressing Pediatric Mental Health during COVID-19 and other Disasters: A National Tabletop Exercise. *Disaster medicine and public health preparedness*. 2021:1-13.

164. Gupta T, Nebhinani N. Impact of COVID-19 pandemic on child and adolescent mental health. *Journal of Indian Association for Child and Adolescent Mental Health*. 2020;16(3):1-16.

165. Hageman JR. Children’s and families’ behavioral and mental health during COVID-19. *Pediatric Annals*. 2020;49(10):e405-e6.

166. Hageman JR. Covid-19 and the mental health effects on adolescents and young adults. *Pediatric Annals*. 2021;50(8):e308-e9.

167. Hester M. AAP issues interim guidance for nutrition in the pandemic. *Contemporary Pediatrics*. 2021;38(2):30-.

168. Hoffman JA, Miller EA. Addressing the Consequences of School Closure Due to COVID-19 on Children's Physical and Mental Well-Being. *World Medical and Health Policy*. 2020;12(3):300-10.

169. Idoiaga N, Berasategi N, Eiguren A et al. Exploring Children's Social and Emotional Representations of the COVID-19 Pandemic. *Frontiers in psychology*. 2020;11:1952.

170. Iqbal SA, Tayyab N. COVID-19 and children: The mental and physical reverberations of the pandemic. *Child: care, health and development*. 2021;47(1):136-9.

171. Jarnig G, Jaunig J, van Poppel MNM. Association of COVID-19 Mitigation Measures With Changes in Cardiorespiratory Fitness and Body Mass Index Among Children Aged 7 to 10 Years in Austria. *JAMA network open*. 2021;4(8):e2121675.

172. Jurak G, Morrison SA, Kovač M et al. A COVID-19 Crisis in Child Physical Fitness: Creating a Barometric Tool of Public Health Engagement for the Republic of Slovenia. *Frontiers in public health*. 2021;9:644235.

173. Leung KKY, Chu SPW, Hon KL et al. Indirect consequences of covid-19 on children’s health. *Hong Kong Medical Journal*. 2021;27(2):160.

174. Li W, Wang Z, Wang G et al. Socioeconomic inequality in child mental health during the COVID-19 pandemic: First evidence from China. *Journal of Affective Disorders*. 2021;287:8-14.

175. Lorch M, Fuchs D. COVID-19: Effects of the shutdown on children and families in child and youth care services in Germany. *Child & Youth Services*. 2020;41(3):290-2.

176. Margaritis I, Houdart S, El Ouadrhiri Y et al. How to deal with COVID-19 epidemic-related lockdown physical inactivity and sedentary increase in youth? Adaptation of Anses' benchmarks. *Archives of Public Health*. 2020;78(1):1-6.

177. Milajerdi HR, Amirshaghaghi F, Milajerdi SR et al. The effect of sedentary behavior on physical and social pain of children during COVID-19 quarantine: Motor and mental recommendation. *Journal of Pain Management*. 2021;14(2):155-8.

178. Mittal VA, Firth J, Kimhy D. Combating the Dangers of Sedentary Activity on Child and Adolescent Mental Health During the Time of COVID-19. *Journal of the American Academy of Child and Adolescent Psychiatry*. 2020;59(11):1197-8.

179. Muratori P, Ciacchini R. Children and the COVID-19 transition: Psychological reflections and suggestions on adapting to the emergency. *Clinical Neuropsychiatry*. 2020;17(2):131-4.

180. Nyberg G. It is time to get a move on and tackle worrying health behaviour patterns in children and adolescents. *Acta Paediatrica*. 2021;110(9):2499-500.

181. O'Kane SM, Lahart IM, Gallagher AM et al. Changes in Physical Activity, Sleep, Mental Health, and Social Media Use During COVID-19 Lockdown Among Adolescent Girls: A Mixed-Methods Study. *Journal of physical activity & health*. 2021;18(6):677-85.

182. Owen A, Bould K. Reduced physical activity and increased sedentary behaviour: the damage on young people during the COVID-19 pandemic. *British Journal of Child Health*. 2021;2(2):64-8.

183. Pagoto SL, Conroy DE. Revitalizing Adolescent Health Behavior after the COVID-19 Pandemic. *JAMA Pediatrics*. 2021;175(7):677-9.

184. Paterson DC, Ramage K, Moore SA et al. Exploring the impact of COVID-19 on the movement behaviors of children and youth: A scoping review of evidence after the first year. *Journal of Sport and Health Science*. 2021;10(6):675-89.

185. Patra S, Patro BK. COVID-19, lockdowns and internet access: Is it pushing adolescents towards suicide? *Journal of Indian Association for Child and Adolescent Mental Health*. 2021;17(2):216-8.

186. Patterson RR, Sornalingam S, Cooper M. Consequences of covid-19 on the childhood obesity epidemic. *The BMJ*. 2021;373.

187. Ramadhan MHA, Putri AK, Melinda D et al. Children’s mental health in the time of COVID-19: How things stand and the aftermath. *Malaysian Journal of Medical Sciences*. 2020;27(5):196-201.

188. Rezaeipour M. COVID-19-related weight gain in school-aged children. *International Journal of Endocrinology and Metabolism*. 2021;19(1).

189. Rice T, Sher L. The men's mental health perspective on adolescent suicide in the COVID-19 era. *Acta neuropsychiatrica*. 2021;33(4):178-81.

190. Sharma V, Dhaliwal S, Singh RB. Psychological and Physical Implications of COVID-19 on School Children in India. Sage Publications, Ltd.; 2021. p. 672-3.

191. Shek DTL. Chinese Adolescent Research Under COVID-19. *Journal of Adolescent Health*. 2020;67(6):733-4.

192. Sinaei R, Pezeshki S, Yazdani M et al. The psychological consequences of covid-19 on children’s world. *Iranian Journal of Child Neurology*. 2021;15(2):87-9.

193. Singh S, Balhara Y. 'Screen-time' for children and adolescents in COVID-19 times: Need to have the contextually informed perspective. *Indian Journal of Psychiatry*. 2021;63(2):192-5.

194. Slomski A. Pediatric Depression and Anxiety Doubled during the Pandemic. *JAMA - Journal of the American Medical Association*. 2021;326(13):1246.

195. Thakur K, Kumar N, Sharma NR. Effect of the Pandemic and Lockdown on Mental Health of Children. *Indian Journal of Pediatrics*. 2020;87(7):552.

196. Tse WWY, Kwan MYW. Impacts of the covid-19 pandemic on the physical and mental health of children. *Hong Kong Medical Journal*. 2021;27(3):175-6.

197. Vyjayanthi NV, Banerjee D, Sathyanarayana Rao TS. The silent victims of the pandemic: Children and adolescents during the COVID-19 crisis. *Journal of Indian Association for Child and Adolescent Mental Health*. 2020;16(3):17-31.

198. Wang G, Zhang Y, Zhao J et al. Mitigate the effects of home confinement on children during the COVID-19 outbreak. *The Lancet*. 2020;395(10228):945-7.

199. Xiang M, Yamamoto S, Mizoue T. Depressive symptoms in students during school closure due to COVID-19 in Shanghai. *Psychiatry and Clinical Neurosciences*. 2020;74(12):664-6.

200. Xiang M, Zhang Z, Kuwahara K. Impact of COVID-19 pandemic on children and adolescents' lifestyle behavior larger than expected. *Progress in cardiovascular diseases*. 2020;63(4):531-2.

201. Zhang X. Association of COVID-19 Mitigation Measures with Cardiorespiratory Fitness and Body Mass Index among Children in Austria. *JAMA Network Open*. 2021;4(8).

202. Agarwal V, Ganesh L, Sunitha BK. Impact of COVID-19 on the mental health among children in China with specific reference to emotional and behavioral disorders. *International Journal of Human Rights in Healthcare*. 2021;14(2):182-8.

203. Ali E. COVID-19, the Child, & Mental health: A Systematic Review. *Annals of Neurology*. 2021;90(SUPPL 26):S116-S7.

204. Cachón-Zagalaz J, Sánchez-Zafra M, Sanabrias-Moreno D et al. Systematic Review of the Literature About the Effects of the COVID-19 Pandemic on the Lives of School Children. *Frontiers in psychology*. 2020;11:569348.

205. Canli M, ÖZÜDoĞRu A, Kara E. COVID-19 Döneminde Çocuklarda Fiziksel Aktivite: Sistematik Derleme. *Turkiye Klinikleri Journal of Sports Sciences*. 2021;13(2):312-7.

206. Nobari H, Fashi M, Eskandari A et al. Effect of covid-19 on health-related quality of life in adolescents and children: A systematic review. *International Journal of Environmental Research and Public Health*. 2021;18(9).

207. O'Loughlin S, Sharaf S, Van Der Spek N. Changing patterns in paediatric attendances during the covid-19 pandemic. *Archives of Disease in Childhood*. 2021;106(SUPPL 1):A345.

208. Okuyama J, Seto S, Fukuda Y et al. Mental health and physical activity among children and adolescents during the COVID-19 pandemic. *Tohoku Journal of Experimental Medicine*. 2021;253(3):203-15.

209. Oliva S, Russo G, Gili R et al. Risks and Protective Factors Associated With Mental Health Symptoms During COVID-19 Home Confinement in Italian Children and Adolescents: The #Understandingkids Study. *Frontiers in Pediatrics*. 2021;9.

210. Racine N, Cooke JE, Eirich R et al. Child and adolescent mental illness during COVID-19: A rapid review. *Psychiatry Research*. 2020;292.

211. Samji H, Wu J, Ladak A et al. Review: Mental health impacts of the COVID-19 pandemic on children and youth – a systematic review. *Child and Adolescent Mental Health*. 2021.

212. Singh A, Shah N, Mbeledogu C et al. Child wellbeing in the United Kingdom following the COVID-19 lockdowns. *Paediatrics and Child Health (United Kingdom)*. 2021;31(12):445-8.

213. Spitzer M. Open schools! Weighing the effects of viruses and lockdowns on children. *Trends in Neuroscience and Education*. 2021;22.

214. Storz MA. The COVID-19 pandemic: An unprecedented tragedy in the battle against childhood obesity. *Korean Journal of Pediatrics*. 2020;63(12):477-82.

215. Ye J. Pediatric Mental and Behavioral Health in the Period of Quarantine and Social Distancing With COVID-19. *JMIR pediatrics and parenting*. 2020;3(2):e19867.

216. El-Osta A, Alaa A, Webber I et al. How is the COVID-19 lockdown impacting the mental health of parents of school-age children in the UK? A cross-sectional online survey. *BMJ open*. 2021;11(5):e043397.

217. Lindsay R, Trott M, Allen P et al. Covid-19 and children’s mental health. *Jornal Brasileiro de Psiquiatria*. 2021;70(2):87-8.

218. Berasategi N, Idoiaga N, Dosil M et al. Design and Validation of a Scale for Measuring Well-Being of Children in Lockdown (WCL). *Frontiers in psychology*. 2020;11:2225.

219. Dzielska AM, Nałȩcz H, Kleszczewska D et al. Consequences of the COVID-19 pandemic on adolescents' health and health behaviour. *Cogent Medicine*. 2021;8.

220. Khan AS. Impact of COVID-19 pandemic and subsequent lockdown on quality of life of high-school students. *Pakistan Journal of Medical and Health Sciences*. 2020;14(4):997-9.

221. Okely AD, Kariippanon KE, Guan H et al. Global effect of COVID-19 pandemic on physical activity, sedentary behaviour and sleep among 3- to 5-year-old children: a longitudinal study of 14 countries. *BMC Public Health*. 2021;21(1):1-15.

222. Phd AL. 78. The Effects of the COVID-19 Pandemic on Physical Activity and Mental Well-Being in Older Adolescents in College. *Journal of Adolescent Health*. 2021;68(2):S42.

223. Salussolia A, Montalti M, Marini S et al. Preliminary data on physical well-being of children and adolescents during the SARS-CoV-2 pandemic...14th European Public Health Conference (Virtual), Public health futures in a changing world, November 10-12, 2021. *European Journal of Public Health*. 2021;31:iii81-iii.

224. Wickramasinghe K. COVID-19: Impacts on child and adolescent obesity and health. *Obesity Facts*. 2021;14(SUPPL 1):1.

225. Luijten M, Muilekom MV, Teela L et al. How the COVID-19 pandemic impacts the psychosocial well-being of children and adolescents in the Netherlands. *Quality of Life Research*. 2019;29(SUPPL 1):S64.

226. Pierce E, Ratnaseelan A, McCracken E et al. Pediatric Headache Experience during the COVID-19 Pandemic. *Annals of Neurology*. 2021;90(SUPPL 26):S46.

227. Weatherspoon BL. Promoting physical activity breaks in Clairton city school district sixth grade classrooms [Ed.D.]: University of Pittsburgh; 2021.

Supplementary Table 4. Distribution of studies by the performance of participants in PA measured by different outcome measures.

| **Authors** | **Measurement** | **Measuring tools** | **Group 1** | **Group 2** | **N for group 1** | **N for group 2** | **N total** |
| --- | --- | --- | --- | --- | --- | --- | --- |
| Acosta et al. (2021) | Participation in sport | Self-designed questionnaire | No | Yes | 84 | 61 | 145 |
| Alonso-Martínez et al. (2021) | Meet recommendation | Wrist-worn geneactiv tri-axial accelerometer | No | Yes | 127 | 12 | 145 |
| Al-Rahamneh et al. (2021) | Meet recommendation | The children’s emotional and behavioural symptoms questionnaire | No | Yes | 905 | 404 | 1309 |
| Alves et al. (2020) | Engaged in VPA | 24-h physical activity recall (PAR) in semi-structured interview | No | Yes | 47 | 17 | 64 |
| Androustsos et al. (2021) | PA change during lockdown | Self-designed questionnaire | Decrease | Increase, no change | 261 | 129 | 390 |
| Awais et al. (2021) | PA levels | Godin Leisure-Time Exercise Questionnaire | Sedentary | Active, moderately active | 87 | 138 | 225 |
| Azoulay et al. (2021) | Participation in PA | Searched in participants' medical files | None | Only at school, at and after school | 14 | 168 | 182 |
| Berasategi et al. (2021) | You get enough physical exercises during the day | “Well-Being of Children in Lockdown (WCL)” scale | Nothing | Few, some, much | 45 | 455 | 500 |
| Berki and Pikó et al. (2021) | Meet recommendation | Based on the questionnaire of the International Health Behaviour in School-aged Children (HBSC) survey | No | Yes | 659 | 46 | 705 |
| Bingham et al. (2021) | Meet recommendation | A modified version of the validated Seven day recall questionnaire, the Youth Activity Profile- English Youth Version (YAP) | No | Yes | 687 | 259 | 946 |
| Breidokiene et al. (2021) | Meet recommendation | Self-designed questionnaire | No | Yes | 208 | 98 | 306 |
| Brzęk et al. (2021) | Meet recommendation | Self-designed questionnaire | No | Yes | 1034 | 277 | 1311 |
| Campbell et al. (2021) | Change in PA | 5 "Pandemic Behaviours" survey | Less PA since school closed | More PA since school closed | 280 | 260 | 540 |
| Chaffee et al. (2021) | Physically active in past week | Self-designed questionnaire | 0-1 day, 2-4 days | 5-7 days | 222 | 261 | 483 |
| Chi et al. (2021) | PA levels | International Physical Activity Questionnaire Short Form | Low | Moderate, High | 734 | 1060 | 1794 |
| Docimo et al. (2021) | Lifestyle habits | Self-designed questionnaire | Sedentary | Moderately active, vigorous physical activity | 118 | 102 | 220 |
| Dragun et al. (2020) | PA levels past 7 days | Self-designed questionnaire | Very low level of activity and rarely active, not physically active at all | Active every day, a few day of the week, sometimes moderately active | 107 | 424 | 531 |
| Dubuc et al. (2020) | Meet recommendation | Self-designed questionnaire | No | Yes | 2174 | 487 | 2661 |
| Dunton et al. (2020) | Less PA in the past 7 days as compared to February 2020 | Self-designed questionnaire | Yes | no | 133 | 78 | 211 |
| Erades et al. (2020) | Change in PA | Self-designed questionnaire | Decrease | Same, increase | 69 | 43 | 112 |
| Ezpeleta et al. (2020) | Practiced PA | Self-designed questionnaire | No | Yes | 92 | 134 | 226 |
| Francisco et al. (2020)1 | Meet recommendation (Italy) | Self-designed questionnaire | No | Yes | 602 | 110 | 712 |
| Francisco et al. (2020)2 | Meet recommendation (Spain) | Self-designed questionnaire | No | Yes | 369 | 61 | 431 |
| Francisco et al. (2020)3 | Meet recommendation (Portugal) | Self-designed questionnaire | No | Yes | 291 | 44 | 335 |
| Ghorbani et al. (2021) | MVPA for more than 30 min per day | The accelerometer ActiGraph wGT3X-BT | No | Yes | 148 | 6 | 154 |
| Gilbert et al. (2021) | Change in PA | The HomeSTEAD’s physical activity and screen media practices and beliefs survey | Decrease | Same, increase | 88 | 56 | 144 |
| Guo et al. (2021) | Meet recommendation | Self-designed questionnaire | No | Yes | 9510 | 906 | 10416 |
| Hyunshik et al. (2021) | Meet recommendation | Triaxial accelerometer (Active Style Pro HJA-750C, Omron Health Care Co., Ltd., Kyoto, Japan) | No | Yes | 51 | 239 | 290 |
| James et al. (2021) | Meet recommendation | The HAPPEN survey | No | Yes | 292 | 776 | 1068 |
| Jester and Kong (2021) | Meet recommendation | Self-designed questionnaire | No | Yes | 19 | 36 | 55 |
| Jolliff et al. (2021) | Participation in exercise | Self-designed questionnaire | No | Yes | 28 | 76 | 134 |
| Jovanović et al. (2021) | Average total weekly PA level | Self-designed questionnaire | Low | Moderate, High | 1035 | 335 | 1370 |
| Kim S. J. et al. (2021) | Spent less time being physically active | Self-designed questionnaire | Yes | No | 174 | 43 | 217 |
| Kim S. Y. et al. (2021)1 | Completed moderate PA more than three times per week | The Korea Youth Risk Behaviour Web-based Survey (KYRBWS) | No | Yes | 35325 | 16814 | 52139 |
| Kim S. Y. et al. (2021)2 | Completed vigorous PA more than three times per week | The Korea Youth Risk Behaviour Web-based Survey (KYRBWS) | No | Yes | 37270 | 14869 | 52139 |
| Lanza et al. (2021) | PA levels | Direct observation using the System for Observing Play and Recreation in Communities (SOPARC) | Sedentary | Light to moderate, vigorous | 95 | 266 | 361 |
| Laurier et al. (2021) | Minutes of PA last week | Self-designed questionnaire | <60mins | >61mins | 50 | 83 | 133 |
| Lee et al. (2021)1 | Frequency of PA | A questionnaire developed by Wallston | Never, once a week, 2-3 times a week | 4 or more a week | 687 | 157 | 844 |
| Lee et al. (2021)2 | Change in PA | A questionnaire developed by Wallston | Less than usual | Same, more than usual | 308 | 447 | 755 |
| Li et al. (2021)1 | Number of exercise session with >30mins/day | Self-designed questionnaire | None, 1-2 days, 3-4 days, 5-6 days | >7 days | 661 | 76 | 737 |
| Li et al. (2021)2 | Exercise change due to the pandemic | Self-designed questionnaire | Less than usual | Same as usual, more than usual | 308 | 447 | 755 |
| Liu et al. (2021) | Number of days with >60 mins of exercise/week | Self-designed questionnaire | 0, 1-2 days | >2 days | 736 | 528 | 1264 |
| Lu et al. (2020) | PA time spent above 1.5 hr/day | International Physical Activity Questionnaire Short Form (IPAQ-SF) | No | Yes | 482 | 483 | 965 |
| Medrano et al. (2021) | Meet recommendation | The Youth Activity Profile” questionnaire (YAP) | No | Yes | 45 | 44 | 89 |
| Mingazova et al. (2021) | Time spent in PA/day | Self-designed questionnaire | 0, <1hr | ≥1 hr | 6142 | 2559 | 8701 |
| Mitra et al. (2020)1 | PA or sport outside | Self-designed questionnaire | Decrease | Increase, same | 939 | 533 | 1472 |
| Mitra et al. (2020)2 | PA or sport inside | Self-designed questionnaire | Decrease | Increase, same | 500 | 972 | 1472 |
| Mitra et al. (2021) | Change in PA | Self-designed questionnaire | Less than before | More than before, no change | 452 | 348 | 800 |
| Morgül et al. (2020) | Meet recommendation | Six-point scale (1=less than 30 minutes - 6=more than 180 minutes) | No | Yes | 453 | 474 | 927 |
| Ng et al. (2020) | Change in PA | PA PACE+ instrument. | Less in lockdown | No change, more in lockdown | 603 | 611 | 1214 |
| Ng et al. (2021)1 | Meet recommendation (boys) | Standardized measures from the HBSC Study | No | Yes | 1220 | 289 | 1509 |
| Ng et al. (2021)2 | Meet recommendation (girls) | Standardized measures from the HBSC Study | No | Yes | 1458 | 344 | 1802 |
| Ng et al. (2021)3 | Meet recommendation (age 10-14) (1st wave) | Standardized measures from the HBSC Study | No | Yes | 1030 | 704 | 1734 |
| Ng et al. (2021)4 | Meet recommendation (age 10-14) (2nd wave) | Standardized measures from the HBSC Study | No | Yes | 975 | 759 | 1734 |
| Ng et al. (2021)5 | Meet recommendation (age 15-18) (1st wave) | Standardized measures from the HBSC Study | No | Yes | 342 | 86 | 428 |
| Ng et al. (2021)6 | Meet recommendation (age 15-18) (2nd wave) | Standardized measures from the HBSC Study | No | Yes | 255 | 173 | 428 |
| Pombo et al. (2021) | Change in PA | Self-designed questionnaire | Much less, less | No change, more, much more | 1560 | 599 | 2159 |
| Qin J. et al. (2021) | Meet recommendation | Self-designed questionnaire | No | Yes | 126 | 62 | 188 |
| Qin Z. et al. (2021) | Meet recommendation | Self-designed questionnaire | No | Yes | 95485 | 30870 | 126355 |
| Sá et al. (2020) | Change in PA | Self-designed questionnaire | Much less, less | Same, more, much more | 678 | 138 | 816 |
| Salzano et al. (2021) | Time spent on PA at home | Self-designed questionnaire | Not practice, <1h/week, 1-3h/week, 4-6h/week | >6h/week | 1527 | 333 | 1860 |
| Siachpazidou et al. (2021) | Impacts of the school closure on PA | Self-designed questionnaire | Moderate, enough, very much | Little, not at all | 223 | 259 | 482 |
| Szwarcwald et al. (2021) | At least 1h of PA twice a week | Self-designed questionnaire | No | Yes | 5512 | 3958 | 9470 |
| Tandon et al. (2021) | Meet recommendation | Self-designed questionnaire | No | Yes | 799 | 195 | 994 |
| Tornaghi et al. (2021) | MET min/week of PA | International Physical Activity Questionnaire (IPAQ) | <700 | 700-2519, >2519 | 102 | 293 | 395 |
| Ventura et al. (2021) | Completed >60min/day | Self-designed questionnaire | Never or <1day/week, 1-2days/week, 3-4days/week | 5-7days/week | 3107 | 357 | 3464 |
| Vuković et al. (2021) | Average in sports recreational activity per day | Self-designed questionnaire | <1/2h, <1h | <2h, <3h, >3h | 187 | 245 | 432 |
| Wang, Hao et al. (2021) | PA time (hr/day) | Self-designed questionnaire | ≤1h | >1h | 3944 | 2962 | 6906 |
| Wang, Zheng et al. (2021) | PA time (hr/day) | Self-designed questionnaire | ≤1h | >1h | 5832 | 5240 | 11072 |
| Wang, Chen et al. (2021) | PA time (hr/day) | Self-designed questionnaire | ≤1h | >1h | 5634 | 6552 | 12186 |
| Zhang et al. (2020) | PA levels past 7 days | International Physical Activity Questionnaire Short Form (IPAQ-SF) for PA level | Low | Moderate, high | 5458 | 4521 | 9979 |
| Zhu et al. (2021) | Change in time spent to exercise | Self-designed questionnaire | Decrease | Increase, same | 953 | 1910 | 2863 |

N=Number of participants

Supplementary Table 5. Distribution of studies by the performance of participants in sleep duration measured by different outcome measures.

| **Authors** | **Measurement** | **Measuring tools** | **Group 1** | **Group 2** | **N for group 1** | **N for group 2** | **N total** |
| --- | --- | --- | --- | --- | --- | --- | --- |
| Alonso-Martínez et al. (2021) | Meet recommendation | Wrist-worn GENEActiv tri-axial accelerometer | No | Yes | 139 | 6 | 145 |
| Al-Rahamneh et al. (2021) | Meet recommendation | The children’s emotional and behavioural symptoms questionnaire | No | Yes | 606 | 678 | 1284 |
| Androustsos et al. (2021) | Average sleep duration during lockdown | Self-designed questionnaire | <8h | 8-10h, >10h | 61 | 336 | 397 |
| Bingham et al. (2021) | Meet recommendation | A modified version of the validated seven day recall questionnaire, the Youth Activity Profile- English Youth Version (YAP) | No | Yes | 287 | 637 | 924 |
| Brzęk et al. (2021) | Meet recommendation | Self-designed questionnaire | No | Yes | 334 | 982 | 1316 |
| Docimo et al. (2021) | Meet recommendation | Self-designed questionnaire | No | Yes | 146 | 74 | 220 |
| Dubuc et al. (2020) | Meet recommendation | the Pittsburgh Sleep Quality Index | No | Yes | 2171 | 490 | 2661 |
| Francisco et al. (2020)1 | Sleep little (Italy) | Self-designed questionnaire | Yes | No | 52 | 660 | 712 |
| Francisco et al. (2020)2 | Sleep little (Spain) | Self-designed questionnaire | Yes | No | 31 | 400 | 431 |
| Francisco et al. (2020)3 | Sleep little (Portugal) | Self-designed questionnaire | Yes | No | 64 | 271 | 335 |
| Guo et al. (2021)1 | Meet recommendation | Self-designed questionnaire | No | Yes | 4011 | 6405 | 10416 |
| Guo et al. (2021)2 | Decreased sleep duration | Self-designed questionnaire | Yes | No | 1750 | 8666 | 10416 |
| Hyunshik et al. (2021) | Meet recommendation | Self-designed questionnaire | No | Yes | 57 | 233 | 290 |
| James et al. (2021) | Meet recommendation | The HAPPEN survey | No | Yes | 970 | 98 | 1068 |
| Jovanović et al. (2021) | Average sleep duration during lockdown | Self-designed questionnaire | <7h | 8-9h, >9 | 808 | 562 | 1370 |
| Kim S. Y. et al. (2021) | Average sleep duration during lockdown | The Korea Youth Risk Behaviour Web-based Survey (KYRBWS) | <6h, 6-7h, 7-8h | >8h | 33726 | 10490 | 44216 |
| López-Gil et al. (2021) | Meet recommendation | Self-designed questionnaire | No | Yes | 61 | 434 | 495 |
| Medrano et al. (2021)1 | Meet recommendation (weekdays) | Sleep time was calculated on the basis of wake-up time and bedtime from the daily log of each child | No | Yes | 99 | 9 | 108 |
| Medrano et al. (2021)2 | Meet recommendation (weekends) | Sleep time was calculated on the basis of wake-up time and bedtime from the daily log of each child | No | Yes | 102 | 6 | 108 |
| Mitra et al. (2020)1 | Change in sleep duration | Self-designed questionnaire | Decrease | Same | 101 | 762 | 863 |
| Mitra et al. (2020)2 | Change in sleep duration | Self-designed questionnaire | Increase | Same | 609 | 762 | 1371 |
| Mitra et al. (2021)1 | Change in sleep duration | Self-designed questionnaire | Less than before | Same | 77 | 283 | 360 |
| Mitra et al. (2021)2 | Change in sleep duration | Self-designed questionnaire | More than before | Same | 440 | 283 | 723 |
| Ng et al. (2021)1 | Meet recommendation (boys) (weekday) | Standardized measures from the HBSC Study through time to bed and wake up | No | Yes | 477 | 1046 | 1523 |
| Ng et al. (2021)2 | Meet recommendation (girls) (weekday) | Standardized measures from the HBSC Study through time to bed and wake up | No | Yes | 541 | 1274 | 1815 |
| Ng et al. (2021)3 | Meet recommendation (boys) (weekend) | Standardized measures from the HBSC Study through time to bed and wake up | No | Yes | 477 | 1046 | 1523 |
| Ng et al. (2021)4 | Meet recommendation (girls) (weekend) | Standardized measures from the HBSC Study through time to bed and wake up | No | Yes | 541 | 1274 | 1815 |
| Ng et al. (2021)5 | Meet recommendation (age 10-14) (1st wave) | Standardized measures from the HBSC Study through time to bed and wake up | No | Yes | 325 | 1409 | 1734 |
| Ng et al. (2021)6 | Meet recommendation (age 10-14) (2nd wave) | Standardized measures from the HBSC Study through time to bed and wake up | No | Yes | 544 | 1190 | 1734 |
| Ng et al. (2021)7 | Meet recommendation (age 15-18) (1st wave) | Standardized measures from the HBSC Study through time to bed and wake up | No | Yes | 134 | 294 | 428 |
| Ng et al. (2021)8 | Meet recommendation (age 15-18) (2nd wave) | Standardized measures from the HBSC Study through time to bed and wake up | No | Yes | 187 | 241 | 428 |
| Pombo et al. (2021)1 | Change in sleep duration | Self-designed questionnaire | Much less, less | No change | 125 | 1114 | 1239 |
| Pombo et al. (2021)2 | Change in sleep duration | Self-designed questionnaire | Much more, more | No change | 917 | 1114 | 2031 |
| Sá et al. (2020)1 | Change in sleep duration | Self-designed questionnaire | Much less, less | No change | 83 | 389 | 472 |
| Sá et al. (2020)2 | Change in sleep duration | Self-designed questionnaire | Much more, more | No change | 344 | 389 | 733 |
| Schnaiderman et al. (2021) | Sleep little | The questionnaire developed by Orgilés et al. | Yes | No | 36 | 231 | 267 |
| Ventura et al. (2021) | Adequate hours of sleep (≥9 h/night for children <11 years, and ≥8 h/night for children ≥11 years) | Self-designed questionnaire | No | Yes | 666 | 2798 | 3464 |

N=Number of participants

Supplementary Table 6. Distribution of studies by the performance of participants in sleep quality measured by different outcome measures.

| **Authors** | **Measurement** | **Measuring tools** | **Group 1** | **Group 2** | **N for group 1** | **N for group 2** | **N total** |
| --- | --- | --- | --- | --- | --- | --- | --- |
| Chi et al. (2021) | Insomnia symptoms | Chinese version of the Youth Self-Rating Insomnia Scales (YSIS) | With symptoms | Without symptoms | 678 | 1116 | 1794 |
| Dragun et al. (2020) | Feeling after waking up | Self-designed questionnaire | Extremely tired and sleepy | Refreshed | 364 | 167 | 531 |
| Dubuc et al. (2020) | Sleep quality (a 4-point scale from “very bad” to “very good”) | the Pittsburgh Sleep Quality Index | Bad | Good | 1250 | 1410 | 2661 |
| Ezpeleta et al. (2020) | Sleep problem | Self-designed questionnaire | Yes | No | 187 | 39 | 226 |
| Francisco et al. (2020)1 | Wakes up frequently (Italy) | Self-designed questionnaire | Yes | No | 70 | 642 | 712 |
| Francisco et al. (2020)2 | Wakes up frequently (Spain) | Self-designed questionnaire | Yes | No | 68 | 363 | 431 |
| Francisco et al. (2020)3 | Wakes up frequently (Portugal) | Self-designed questionnaire | Yes | No | 42 | 293 | 335 |
| Ghanamah and Eghbaria-Ghanamah (2021) | Sleep Problem (difficulty falling asleep, agitation during sleep, frequent awakenings) | Self-designed questionaire | Yes | No | 158 | 224 | 382 |
| Gilbert et al. (2021) | Difficulty sleeping | Child Mood and Feelings Questionnaire | Yes | No | 20 | 124 | 144 |
| López-Gil et al. (2021) | Sleep related problems | Self-designed questionnaire | Yes | No | 253 | 242 | 495 |
| MacKenzie et al. (2021) | Change in sleep quality | Self-designed questionnaire | Worse than before | Better, same as before | 34 | 51 | 85 |
| Mitra et al. (2020) | Change in sleep quality | Self-designed questionnaire | Decrease | Increase, same | 209 | 1263 | 1472 |
| Mitra et al. (2021) | Change in sleep quality | Self-designed questionnaire | Worse than before | Same as before, better than before | 115 | 658 | 800 |
| Reséndiz-Aparicio (2021)1 | Sleeping problem increased due to confinement | Self-designed questionnaire | Yes | No | 814 | 3186 | 4000 |
| Reséndiz-Aparicio (2021)2 | Sleeping troubles | Self-designed questionnaire | Difficulty starting to sleep, awakenings at night, nightmares, night terrors, bed-wetting, sleepwalking | None | 2356 | 1446 | 3802 |
| Salzano et al. (2021) | Modification of sleep-weak rhythm | Self-designed questionnaire | Yes | No | 1516 | 344 | 1860 |
| Schnaiderman et al. (2021)1 | Wakes up frequently | The questionnaire developed by Orgilés et al | Yes | No | 41 | 226 | 267 |
| Schnaiderman et al. (2021)2 | Has nightmares | The questionnaire developed by Orgilés et al | Yes | No | 40 | 227 | 267 |
| Schnaiderman et al. (2021)3 | Has sleeping difficulties | The questionnaire developed by Orgilés et al | Yes | No | 83 | 184 | 267 |
| Siachpazidou et al. (2021) | Impacts of the school closures on sleep quality | Self-designed questionnaire | Moderate, enough, very much | Little, not at all | 277 | 205 | 482 |
| Szwarcwald et al. (2021) | Sleep problem | Self-designed questionnaire | Started to have, got worse | Never, as usual | 3385 | 6012 | 9397 |
| Ventura et al. (2021)1 | Delay bedtime | Self-designed questionnaire | Yes | No | 2745 | 719 | 3464 |
| Ventura et al. (2021)2 | Suspected disorders of initiating and maintain sleep | Self-designed questionnaire | Yes | No | 563 | 2901 | 3464 |
| Wang, Zheng et al. (2021) | Sleep problem | Self-designed questionnaire | Yes | No | 3288 | 7784 | 11072 |
| Wang, Chen et al. (2021) | Sleep problem | Self-designed questionnaire | Yes | No | 3722 | 8464 | 12186 |

Supplementary Table 7. Distribution of studies by the performance of participants in psychological problems measured by different outcome measures.

| **Authors** | **Measurement** | **Measuring tools** | **Group 1** | **Group 2** | **N for group 1** | **N for group 2** | **N total** |
| --- | --- | --- | --- | --- | --- | --- | --- |
| Acosta et al. (2021)1 | Anxiety-related symptoms | Self-designed questionnaire | At risk | No risk | 61 | 84 | 145 |
| Acosta et al. (2021)2 | Depressive symptoms | Self-designed questionnaire | At risk | No risk | 65 | 80 | 145 |
| Acosta et al. (2021)3 | OCD symptoms | Self-designed questionnaire | At risk | No risk | 60 | 85 | 145 |
| Al-Rahamneh et al. (2021)1 | My child is worried | The children’s emotional and behavioural symptoms questionnaire | Somewhat more, much more | Much less, somewhat less, same | 507 | 802 | 1309 |
| Al-Rahamneh et al. (2021)2 | My child is anxious | The children’s emotional and behavioural symptoms questionnaire | Somewhat more, much more | Much less, somewhat less, same | 586 | 723 | 1309 |
| Al-Rahamneh et al. (2021)3 | My child is restless | The children’s emotional and behavioural symptoms questionnaire | Somewhat more, much more | Much less, somewhat less, same | 637 | 672 | 1309 |
| Al-Rahamneh et al. (2021)4 | My child is sad | The children’s emotional and behavioural symptoms questionnaire | Somewhat more, much more | Much less, somewhat less, same | 568 | 741 | 1309 |
| Al-Rahamneh et al. (2021)5 | My child is nervous | The children’s emotional and behavioural symptoms questionnaire | Somewhat more, much more | Much less, somewhat less, same | 718 | 591 | 1309 |
| Al-Rahamneh et al. (2021)6 | My child feels lonely | The children’s emotional and behavioural symptoms questionnaire | Somewhat more, much more | Much less, somewhat less, same | 687 | 622 | 1309 |
| Awais et al. (2021) | Psychological distress | Kessler-10 well | Mildly unwell, moderately unwell, severely unwell | Well | 80 | 145 | 225 |
| Berasategi et al. (2021)1 | Feel more nervous than usual | “Well-Being of Children in Lockdown (WCL)” scale | Few, some, much | Nothing | 425 | 75 | 500 |
| Berasategi et al. (2021)2 | Get angry more than usual | “Well-Being of Children in Lockdown (WCL)” scale | Few, some, much | Nothing | 406 | 94 | 500 |
| Berasategi et al. (2021)3 | Feel sader than usual | “Well-Being of Children in Lockdown (WCL)” scale | Few, some, much | Nothing | 445 | 55 | 500 |
| Berki and Pikó et al. (2021) | Risk of depression | Child Depression Inventory (CDI) | High | Low | 318 | 383 | 701 |
| Breidokiene et al. (2021) | Change in emotional well-being/ behaviour during lockdown | Self-designed questionnaire | Decrease | Increase, no change | 96 | 210 | 306 |
| Chaffee et al. (2021) | Depressive symptoms | Self-designed questionnaire | Positive screen | Negative screen | 153 | 328 | 481 |
| Chi et al. (2021)1 | Depressive symptoms | 9-item Patient Health Questionnaire | With symptoms | Without symptoms | 864 | 930 | 1794 |
| Chi et al. (2021)2 | Anxiety symptoms | Generalized Anxiety Disorder scale | With symptoms | Without symptoms | 659 | 1135 | 1794 |
| Dragun et al. (2020) | Perceived stress category | The Perceived Stress Scale (PSS-10) questionnaire | High | Low, Moderate | 83 | 448 | 531 |
| Erades et al. (2020) | Feel depressed | Self-designed questionnaire | Yes | No | 26 | 86 | 112 |
| Ezpeleta et al. (2020) | Negative feelings | The Strengths and Difficulties Questionnaire (SDQ) | Yes | No | 45 | 181 | 226 |
| Francisco et al. (2020)1 | My child is worried (Italy) | Self-designed questionnaire | Yes | No | 226 | 486 | 712 |
| Francisco et al. (2020)2 | My child is worried (Spain) | Self-designed questionnaire | Yes | No | 118 | 313 | 431 |
| Francisco et al. (2020)3 | My child is worried (Portugal) | Self-designed questionnaire | Yes | No | 151 | 184 | 335 |
| Francisco et al. (2020)4 | My child is anxious (Italy) | Self-designed questionnaire | Yes | No | 146 | 566 | 712 |
| Francisco et al. (2020)5 | My child is anxious (Spain) | Self-designed questionnaire | Yes | No | 179 | 252 | 431 |
| Francisco et al. (2020)6 | My child is anxious (Portugal) | Self-designed questionnaire | Yes | No | 121 | 214 | 335 |
| Francisco et al. (2020)7 | My child is restless (Italy) | Self-designed questionnaire | Yes | No | 247 | 465 | 712 |
| Francisco et al. (2020)8 | My child is restless (Spain) | Self-designed questionnaire | Yes | No | 196 | 235 | 431 |
| Francisco et al. (2020)9 | My child is restless (Portugal) | Self-designed questionnaire | Yes | No | 120 | 215 | 335 |
| Ghanamah and Eghbaria-Ghanamah (2021)1 | Experienced any mood swings | An ad-hoc questionnaire (self-designed) | Yes | No | 309 | 73 | 382 |
| Ghanamah and Eghbaria-Ghanamah (2021)2 | Been nervous | An ad-hoc questionnaire (self-designed) | Yes | No | 265 | 117 | 382 |
| Ghorbani et al. (2021)1 | Mild and moderate depression symptoms | The Depression, Anxiety, Stress Scale-21 (DASS-21) | Yes | No | 126 | 28 | 154 |
| Ghorbani et al. (2021)2 | Mild and moderate levels of anxiety | The Depression, Anxiety, Stress Scale-21 (DASS-21) | Yes | No | 138 | 16 | 154 |
| Ghorbani et al. (2021)3 | Mild and moderate symptoms of stress | The Depression, Anxiety, Stress Scale-21 (DASS-21) | Yes | No | 88 | 66 | 154 |
| Gilbert et al. (2021)1 | Worried | Child Mood and Feelings Questionnaire | Yes | No | 25 | 119 | 144 |
| Gilbert et al. (2021)2 | Angry | Child Mood and Feelings Questionnaire | Yes | No | 30 | 114 | 144 |
| Gilbert et al. (2021)3 | Restless | Child Mood and Feelings Questionnaire | Yes | No | 27 | 117 | 144 |
| Gilbert et al. (2021)4 | Unhappy | Child Mood and Feelings Questionnaire | Yes | No | 16 | 128 | 144 |
| Gilbert et al. (2021)5 | Lonely | Child Mood and Feelings Questionnaire | Yes | No | 48 | 96 | 144 |
| Gilbert et al. (2021)6 | Change in mental well-being | Child Mood and Feelings Questionnaire | Worse | Same, Better | 106 | 36 | 144 |
| James et al. (2021) | Emotional difficulties | Me and my feeling survey | At risk | No risk | 130 | 938 | 1068 |
| Lee et al. (2021) | Loneliness | Questionnaire modified and supplemented by Anspaugh et al. | Some of the time, often | Hardly ever | 624 | 130 | 754 |
| Li et al. (2021) | Mental health change | Self-designed questionnaire | A little worse, a lot worse | The same, a little better, a lot worse | 567 | 193 | 760 |
| Mitra et al. (2021)1 | More alone | Russell’s theorization of psychological construct of emotions | Yes | No | 65 | 735 | 800 |
| Mitra et al. (2021)2 | Sadder | Russell’s theorization of psychological construct of emotions | Yes | No | 145 | 655 | 800 |
| Mitra et al. (2021)3 | Angrier | Russell’s theorization of psychological construct of emotions | Yes | No | 108 | 692 | 800 |
| Mitra et al. (2021)4 | More worried | Russell’s theorization of psychological construct of emotions | Yes | No | 248 | 552 | 800 |
| Morgül et al. (2020)1 | Worried | Children’s emotional and behavioural symptoms questionnaire | Somewhat more, much more | Much less, somewhat less, same | 484 | 440 | 924 |
| Morgül et al. (2020)2 | Restless | Children’s emotional and behavioural symptoms questionnaire | Somewhat more, much more | Much less, somewhat less, same | 487 | 495 | 982 |
| Morgül et al. (2020)3 | Anxious | Children’s emotional and behavioural symptoms questionnaire | Somewhat more, much more | Much less, somewhat less, same | 418 | 507 | 925 |
| Morgül et al. (2020)4 | Sad | Children’s emotional and behavioural symptoms questionnaire | Somewhat more, much more | Much less, somewhat less, same | 398 | 520 | 918 |
| Morgül et al. (2020)5 | Lonely | Children’s emotional and behavioural symptoms questionnaire | Somewhat more, much more | Much less, somewhat less, same | 594 | 327 | 921 |
| Morgül et al. (2020)6 | Nervous | Children’s emotional and behavioural symptoms questionnaire | Somewhat more, much more | Much less, somewhat less, same | 339 | 580 | 919 |
| Morgül et al. (2020)7 | Angry | Children’s emotional and behavioural symptoms questionnaire | Somewhat more, much more | Much less, somewhat less, same | 448 | 473 | 921 |
| Ng et al. (2021) | Feeling lonely | R-UCLA Scale | Yes | No | 492 | 2926 | 3418 |
| Qin J. et al. (2021) | 100 items Mental Health Test (MHT) with 8 subcategories: learning anxiety, personal anxiety, loneliness anxiety, self-blaming tendency, sensitivity tendency, somatic anxiety, phobia anxiety, and impulsive tendency | 100 items Mental Health Test (MHT) | Score >55 | Score ≤55 | 60 | 188 | 248 |
| Qin Z. et al. (2021)1 | Psychological distress (age <8) | Chinese version of 12-item General Health Questionnaire (GHQ-12) | Score ≥3 | Score <3 | 6561 | 80 627 | 87 188 |
| Qin Z. et al. (2021)2 | Psychological distress (age 8-10) | Chinese version of 12-item General Health Questionnaire (GHQ-12) | Score ≥4 | Score <4 | 24625 | 278580 | 303205 |
| Qin Z. et al. (2021)3 | Psychological distress (age 11-13) | Chinese version of 12-item General Health Questionnaire (GHQ-12) | Score ≥5 | Score <5 | 39100 | 367630 | 406730 |
| Qin Z. et al. (2021)4 | Psychological distress (age 14-16) | Chinese version of 12-item General Health Questionnaire (GHQ-12) | Score ≥6 | Score <6 | 41630 | 270415 | 312045 |
| Qin Z. et al. (2021)5 | Psychological distress (age >16) | Chinese version of 12-item General Health Questionnaire (GHQ-12) | Score ≥7 | Score <7 | 14439 | 75713 | 90152 |
| Salzano et al. (2021) | Feelings of fear, discouragement | Self-designed questionnaire | Yes | No | 1123 | 737 | 1860 |
| Schnaiderman et al. (2021)1 | Is worried | Questionnaire developed by Orgilés et al. | Yes | No | 107 | 160 | 267 |
| Schnaiderman et al. (2021)2 | Is anxious | Questionnaire developed by Orgilés et al. | Yes | No | 145 | 122 | 267 |
| Schnaiderman et al. (2021)3 | Is sad | Questionnaire developed by Orgilés et al. | Yes | No | 114 | 123 | 267 |
| Schnaiderman et al. (2021)4 | Is reluctant | Questionnaire developed by Orgilés et al. | Yes | No | 152 | 115 | 267 |
| Schnaiderman et al. (2021)5 | Feels lonely | Questionnaire developed by Orgilés et al. | Yes | No | 119 | 148 | 267 |
| Schnaiderman et al. (2021)6 | Is nervous | Questionnaire developed by Orgilés et al. | Yes | No | 100 | 167 | 267 |
| Schnaiderman et al. (2021)7 | Is angry | Questionnaire developed by Orgilés et al. | Yes | No | 146 | 121 | 267 |
| Schnaiderman et al. (2021)8 | Feels frustrated | Questionnaire developed by Orgilés et al. | Yes | No | 134 | 133 | 267 |
| Siachpazidou et al. (2021)1 | Impacts of the school closures on psychological symptoms | Self-designed questionnaire | Moderate, enough, very much | Little, not at all | 249 | 233 | 482 |
| Siachpazidou et al. (2021)2 | Anxiety | Self-designed questionnaire | Yes | No | 388 | 94 | 482 |
| Siachpazidou et al. (2021)3 | Anguish | Self-designed questionnaire | Yes | No | 403 | 79 | 482 |
| Siachpazidou et al. (2021)4 | Fear | Self-designed questionnaire | Yes | No | 437 | 45 | 482 |
| Siachpazidou et al. (2021)5 | Nightmare | Self-designed questionnaire | Yes | No | 434 | 48 | 482 |
| Szwarcwald et al. (2021) | Sadness | Adapted from the World Health Survey | Sometimes, often, always | Never, seldom | 6268 | 3142 | 9410 |
| Vuković et al. (2021)1 | Sensitivity | Self-designed questionnaire | Yes | No | 119 | 331 | 450 |
| Vuković et al. (2021)2 | Worry | Self-designed questionnaire | Yes | No | 134 | 316 | 450 |
| Vuković et al. (2021)3 | Anxiety | Self-designed questionnaire | Yes | No | 172 | 278 | 450 |
| Vuković et al. (2021)4 | Nervousness | Self-designed questionnaire | Yes | No | 115 | 325 | 450 |
| Wang, Hao et al. (2021)1 | Schizoid (age 6-11 boys) | Achenbach Child Behaviour Checklist | Positive | Negative | 76 | 1870 | 1946 |
| Wang, Hao et al. (2021)2 | Depress (age 6-11 boys) | Achenbach Child Behaviour Checklist | Positive | Negative | 74 | 1872 | 1946 |
| Wang, Hao et al. (2021)3 | Schizoid (age 12-16 boys) | Achenbach Child Behaviour Checklist | Positive | Negative | 26 | 1620 | 1646 |
| Wang, Hao et al. (2021)4 | Depress (age 6-11 girls) | Achenbach Child Behaviour Checklist | Positive | Negative | 32 | 1648 | 1680 |
| Wang, Hao et al. (2021)5 | Schizoid (age 12-16 girls) | Achenbach Child Behaviour Checklist | Positive | Negative | 121 | 1513 | 1634 |
| Wang, Hao et al. (2021)6 | Anxiety and OCD (age 12-16 girls) | Achenbach Child Behaviour Checklist | Positive | Negative | 48 | 1586 | 1634 |
| Wang, Hao et al. (2021)7 | Depress and anti-social (age 12-16 girls) | Achenbach Child Behaviour Checklist | Positive | Negative | 48 | 1586 | 1634 |

N=Number of participants

Supplementary Table 8. Distribution of studies by the performance of participants in behavioural problems measured by different outcome measures.

| **Authors** | **Measurement** | **Measuring tools** | **Outcome 1** | **Outcome 2** | **N for outcome 1** | **N for outcome 2** | **N total** |
| --- | --- | --- | --- | --- | --- | --- | --- |
| Al-Rahamneh et al. (2021)1 | My child argues with the rest of the family | The children’s emotional and behavioural symptoms questionnaire | Somewhat more, much more | Much less, somewhat less, same | 795 | 514 | 1309 |
| Al-Rahamneh et al. (2021)2 | My child has behavioural problems | The children’s emotional and behavioural symptoms questionnaire | Somewhat more, much more | Much less, somewhat less, same | 332 | 977 | 1309 |
| Al-Rahamneh et al. (2021)3 | My child is irritable | The children’s emotional and behavioural symptoms questionnaire | Somewhat more, much more | Much less, somewhat less, same | 864 | 445 | 1309 |
| Al-Rahamneh et al. (2021)4 | My child cries easily | The children’s emotional and behavioural symptoms questionnaire | Somewhat more, much more | Much less, somewhat less, same | 619 | 690 | 1309 |
| Al-Rahamneh et al. (2021)5 | My child has difficulty concentrating | The children’s emotional and behavioural symptoms questionnaire | Somewhat more, much more | Much less, somewhat less, same | 603 | 706 | 1309 |
| Al-Rahamneh et al. (2021)6 | My child is very dependent on us | The children’s emotional and behavioural symptoms questionnaire | Somewhat more, much more | Much less, somewhat less, same | 579 | 730 | 1309 |
| Berasategi et al. (2021)1 | Cry more than usual | “Well-Being of Children in Lockdown (WCL)” scale | Few, some, much | Nothing | 457 | 43 | 500 |
| Berasategi et al. (2021)2 | Eating more than usual during lockdown | “Well-Being of Children in Lockdown (WCL)” scale | Few, some, much | Nothing | 49 | 451 | 500 |
| Berasategi et al. (2021)3 | Eating more treats | “Well-Being of Children in Lockdown (WCL)” scale | Few, some, much | Nothing | 46 | 454 | 500 |
| Berasategi et al. (2021)4 | Over-using new technology | “Well-Being of Children in Lockdown (WCL)” scale | Few, some, much | Nothing | 240 | 260 | 500 |
| Berasategi et al. (2021)5 | Watching too many TV programs, cartoons, or movies | “Well-Being of Children in Lockdown (WCL)” scale | Few, some, much | Nothing | 111 | 389 | 500 |
| Berki and Pikó et al. (2021)1 | Alcohol consumption | Separate single questions | Occasionally, daily | Never | 292 | 413 | 705 |
| Berki and Pikó et al. (2021)2 | Drug abuse | Separate single questions | Occasionally, daily | Never | 21 | 684 | 705 |
| Berki and Pikó et al. (2021)3 | Tobacco smoking | Separate single questions | Occasionally, daily | Never | 140 | 565 | 705 |
| Chaffee et al. (2021)1 | E-cigarette use in past 30d | Self-designed questionnaire | 1-5d, 6-30 days | Never, ever (0 day) | 97 | 388 | 485 |
| Chaffee et al. (2021)2 | Other tobacco use in past 30d | Self-designed questionnaire | 1-30 days | 0 day | 34 | 487 | 485 |
| Chaffee et al. (2021)3 | Cannabis use in past 30d | Self-designed questionnaire | 1-5d, 6-30 days | Never, ever (0 day) | 95 | 390 | 485 |
| Chaffee et al. (2021)4 | Alcohol use in past 30d | Self-designed questionnaire | 1-30 days | 0 day | 386 | 98 | 485 |
| Erades et al. (2020)1 | Irritability | Self-designed questionnaire | Yes | No | 32 | 80 | 112 |
| Erades et al. (2020)2 | Difficulty concentrating | Self-designed questionnaire | Yes | No | 27 | 85 | 112 |
| Ezpeleta et al. (2020) | Worsen family relationship | The Strengths and Difficulties Questionnaire (SDQ) | Yes | No | 28 | 198 | 226 |
| Francisco et al. (2020)1 | My child argues with the rest of the family (Italy) | Self-designed questionnaire | Yes | No | 165 | 547 | 712 |
| Francisco et al. (2020)2 | My child argues with the rest of the family (Spain) | Self-designed questionnaire | Yes | No | 174 | 257 | 431 |
| Francisco et al. (2020)3 | My child argues with the rest of the family (Portugal) | Self-designed questionnaire | Yes | No | 108 | 227 | 335 |
| Francisco et al. (2020)4 | My child is irritable (Italy) | Self-designed questionnaire | Yes | No | 260 | 452 | 712 |
| Francisco et al. (2020)5 | My child is irritable (Spain) | Self-designed questionnaire | Yes | No | 186 | 245 | 431 |
| Francisco et al. (2020)6 | My child is irritable (Portugal) | Self-designed questionnaire | Yes | No | 152 | 183 | 335 |
| Francisco et al. (2020)7 | My child has behavioural problems (Italy) | Self-designed questionnaire | Yes | No | 57 | 655 | 712 |
| Francisco et al. (2020)8 | My child My child has behavioural problems (Spain) | Self-designed questionnaire | Yes | No | 128 | 303 | 431 |
| Francisco et al. (2020)9 | My child My child has behavioural problems (Portugal) | Self-designed questionnaire | Yes | No | 61 | 274 | 335 |
| Francisco et al. (2020)10 | My child has has difficulty concentrating (Italy) | Self-designed questionnaire | Yes | No | 135 | 577 | 712 |
| Francisco et al. (2020)11 | My child My child has difficulty concentrating (Spain) | Self-designed questionnaire | Yes | No | 133 | 298 | 431 |
| Francisco et al. (2020)12 | My child My child has difficulty concentrating (Portugal) | Self-designed questionnaire | Yes | No | 85 | 250 | 335 |
| Ghanamah and Eghbaria-Ghanamah (2021)1 | Irritability (Intolerance to Rules, Caprices, Excessive Demands) | An ad-hoc questionnaire（self-designed） | Yes | No | 253 | 129 | 382 |
| Ghanamah and Eghbaria-Ghanamah (2021)2 | Did Your Child Seem Lazier Than He Was before the Pandemic | An ad-hoc questionnaire（self-designed） | Yes | No | 255 | 127 | 382 |
| Gilbert et al. (2021) | Difficulty concentrating | Child Mood and Feelings Questionnaire | Yes | No | 31 | 113 | 144 |
| James et al. (2021) | Behavioural difficulties | Me and my feeling survey | Yes | No | 95 | 973 | 1068 |
| Kim S. J. et al. (2021)1 | Children didn't do their homework without supervision | The Behaviour Problem Index (BPI) | Yes | No | 47 | 170 | 217 |
| Kim S. J. et al. (2021)2 | Children didn't do homework with supervision | The Behaviour Problem Index (BPI) | Yes | No | 9 | 208 | 217 |
| Kim S. Y. et al. (2021)1 | Drinking alcohol in the recent 30d | The Korea Youth Risk Behaviour Web-based Survey (KYRBWS) | Yes | No | 5460 | 46670 | 52130 |
| Kim S. Y. et al. (2021)2 | Smoking in the recent 30d | The Korea Youth Risk Behaviour Web-based Survey (KYRBWS) | Yes | No | 225 | 49886 | 50111 |
| Morgül et al. (2020)1 | Argues with the rest of the family | Children’s emotional and behavioural symptoms questionnaire | Somewhat more, much more | Much less, somewhat less, same | 522 | 399 | 921 |
| Morgül et al. (2020)2 | Cries easily | Children’s emotional and behavioural symptoms questionnaire | Somewhat more, much more | Much less, somewhat less, same | 352 | 565 | 917 |
| Morgül et al. (2020)3 | Is irritable | Children’s emotional and behavioural symptoms questionnaire | Somewhat more, much more | Much less, somewhat less, same | 524 | 394 | 918 |
| Morgül et al. (2020)4 | Has behavioural problems | Children’s emotional and behavioural symptoms questionnaire | Somewhat more, much more | Much less, somewhat less, same | 241 | 679 | 920 |
| Morgül et al. (2020)5 | Difficulty concentrating | Children’s emotional and behavioural symptoms questionnaire | Somewhat more, much more | Much less, somewhat less, same | 386 | 533 | 919 |
| Reséndiz-Aparicio (2021)1 | Behaviour change (Tantrum or low tolerance, frequent mood swings, difficulty in social relationships with siblings and parents, aggressive behaviour) | Self-designed questionnaire | Yes | No | 2960 | 880 | 3840 |
| Reséndiz-Aparicio (2021)2 | 1st alcohol last 30 days | Self-designed questionnaire | 1-30 days | None | 852 | 590 | 1442 |
| Reséndiz-Aparicio (2021)3 | 2nd alcohol last 30 days | Self-designed questionnaire | 1-30 days | None | 389 | 174 | 563 |
| Reséndiz-Aparicio (2021)4 | 1st cannabis last 30days | Self-designed questionnaire | 1-30 days | None | 200 | 1242 | 1442 |
| Reséndiz-Aparicio (2021)5 | 2nd cannabis last 30days | Self-designed questionnaire | 1-30 days | None | 44 | 519 | 563 |
| Schnaiderman et al. (2021)1 | Has behavioural problems | Questionnaire developed by Orgilés et al. | Yes | No | 53 | 214 | 267 |
| Schnaiderman et al. (2021)2 | Is bored | Questionnaire developed by Orgilés et al. | Yes | No | 205 | 62 | 267 |
| Schnaiderman et al. (2021)3 | Is irritable | Questionnaire developed by Orgilés et al. | Yes | No | 158 | 109 | 267 |
| Schnaiderman et al. (2021)4 | Is easily alarmed | Questionnaire developed by Orgilés et al. | Yes | No | 57 | 210 | 267 |
| Wang, Hao et al. (2021)1 | Communication problems (age 6-11 boys) | Achenbach Child Behaviour Checklist | Positive | Negative | 71 | 1875 | 1946 |
| Wang, Hao et al. (2021)2 | Social withdrawal (age 6-11 boys) | Achenbach Child Behaviour Checklist | Positive | Negative | 49 | 1897 | 1946 |
| Wang, Hao et al. (2021)3 | Aggression (age 6-11 boys) | Achenbach Child Behaviour Checklist | Positive | Negative | 68 | 1878 | 1946 |
| Wang, Hao et al. (2021)4 | Hyperactivities (age 6-11 boys) | Achenbach Child Behaviour Checklist | Positive | Negative | 47 | 1899 | 1946 |
| Wang, Hao et al. (2021)5 | Anti-social (age 6-11 boys) | Achenbach Child Behaviour Checklist | Positive | Negative | 30 | 1916 | 1946 |
| Wang, Hao et al. (2021)6 | Communication problems (age 12-16 boys) | Achenbach Child Behaviour Checklist | Positive | Negative | 34 | 1612 | 1646 |
| Wang, Hao et al. (2021)7 | Immature (age 12-16 boys) | Achenbach Child Behaviour Checklist | Positive | Negative | 51 | 1595 | 1646 |
| Wang, Hao et al. (2021)8 | Anti-social (age 12-16 boys) | Achenbach Child Behaviour Checklist | Positive | Negative | 29 | 1617 | 1646 |
| Wang, Hao et al. (2021)9 | Aggression (age 12-16 boys) | Achenbach Child Behaviour Checklist | Positive | Negative | 53 | 1593 | 1646 |
| Wang, Hao et al. (2021)10 | Hyperactivities (age 12-16 boys) | Achenbach Child Behaviour Checklist | Positive | Negative | 95 | 1551 | 1646 |
| Wang, Hao et al. (2021)11 | Social withdrawal (age 6-11 girls) | Achenbach Child Behaviour Checklist | Positive | Negative | 33 | 1647 | 1680 |
| Wang, Hao et al. (2021)12 | Hyperactivities (age 6-11 girls) | Achenbach Child Behaviour Checklist | Positive | Negative | 64 | 1616 | 1680 |
| Wang, Hao et al. (2021)13 | Aggression (age 6-11 girls) | Achenbach Child Behaviour Checklist | Positive | Negative | 32 | 1648 | 1680 |
| Wang, Hao et al. (2021)14 | Anti-social (age 6-11 girls) | Achenbach Child Behaviour Checklist | Positive | Negative | 29 | 1651 | 1680 |
| Wang, Hao et al. (2021)15 | Immature (age 12-16 girls) | Achenbach Child Behaviour Checklist | Positive | Negative | 43 | 1591 | 1634 |
| Wang, Hao et al. (2021)16 | Anti-social (age 12-16 girls) | Achenbach Child Behaviour Checklist | Positive | Negative | 46 | 1588 | 1634 |
| Wang, Hao et al. (2021)17 | Aggression (age 12-16 girls) | Achenbach Child Behaviour Checklist | Positive | Negative | 45 | 1589 | 1634 |

N=Number of participants

Supplementary Table 9. Downs and Black assessment of included studies.

| **Authors** | **Downs and Black items (Yes=1/N=0)** | | | | | | | |
| --- | --- | --- | --- | --- | --- | --- | --- | --- |
|  | **Clearly stated aim** | **Clearly defined study population** | **Study sample representative of the source population** | **Attempt made to adjust for confounding** | **Attempt made to validate survey response to institutional records where possible** | **Discussion of study limitation** | **Total** | **Quality grading (High=5-6/Moderate=3-4/Low=0-2)** |
| Acosta et al. (2021) | 1 | 1 | 1 | 0 | 1 | 1 | 5 | High |
| Alonso-Martínez et al. (2021) | 1 | 1 | 1 | 0 | 1 | 1 | 5 | High |
| Al-Rahamneh,et al. (2021) | 1 | 1 | 1 | 0 | 1 | 1 | 5 | High |
| Alves et al. (2020) | 1 | 1 | 1 | 0 | 1 | 1 | 5 | High |
| Androutsos, et al. (2021) | 1 | 1 | 1 | 0 | 0 | 1 | 4 | Moderate |
| Awais et al. (2021) | 1 | 1 | 1 | 0 | 1 | 1 | 5 | High |
| Azoulay et al. (2021) | 1 | 1 | 1 | 0 | 0 | 1 | 4 | Moderate |
| Berasategi et al. (2021) | 1 | 1 | 1 | 0 | 1 | 1 | 5 | High |
| Berki and Pikó et al. (2021) | 1 | 1 | 1 | 0 | 1 | 1 | 5 | High |
| Bingham et al. (2021) | 1 | 1 | 1 | 0 | 1 | 1 | 5 | High |
| Breidokiene et al. (2021) | 1 | 1 | 1 | 0 | 1 | 1 | 5 | High |
| Brzęk et al. (2021) | 1 | 1 | 1 | 0 | 0 | 1 | 4 | Moderate |
| Campbell et al. (2021) | 1 | 1 | 1 | 0 | 1 | 1 | 5 | High |
| Chaffee et al. (2021) | 1 | 1 | 1 | 1 | 0 | 1 | 5 | High |
| Chi et al. (2021) | 1 | 1 | 1 | 0 | 1 | 1 | 5 | High |
| Docimo et al. (2021) | 1 | 1 | 1 | 0 | 1 | 1 | 5 | High |
| Dragun et al. (2020) | 1 | 1 | 1 | 0 | 1 | 1 | 5 | High |
| Dubuc et al. (2020) | 1 | 1 | 1 | 0 | 1 | 1 | 5 | High |
| Dunton et al. (2020) | 1 | 1 | 1 | 0 | 1 | 1 | 5 | High |
| Erades et al. (2020) | 1 | 1 | 1 | 0 | 1 | 1 | 5 | High |
| Ezpeleta et al. (2020) | 1 | 1 | 1 | 0 | 1 | 1 | 5 | High |
| Francisco et al. (2020) | 1 | 1 | 1 | 0 | 1 | 1 | 5 | High |
| Ghanamah and Eghbaria-Ghanamah (2021) | 1 | 1 | 1 | 0 | 1 | 1 | 5 | High |
| Ghorbani et al. (2021) | 1 | 1 | 1 | 0 | 1 | 1 | 5 | High |
| Gilbert et al. (2021) | 1 | 1 | 1 | 0 | 1 | 1 | 5 | High |
| Guo et al. (2021) | 1 | 1 | 1 | 0 | 1 | 1 | 5 | High |
| Hyunshik et al. (2021) | 1 | 1 | 1 | 0 | 1 | 1 | 5 | High |
| James et al. (2021) | 1 | 1 | 1 | 0 | 1 | 1 | 5 | High |
| Jester and Kong (2021) | 1 | 1 | 1 | 0 | 1 | 1 | 5 | High |
| Jolliff et al. (2021) | 1 | 1 | 1 | 0 | 0 | 1 | 4 | Moderate |
| Jovanović et al. (2021) | 1 | 1 | 1 | 0 | 0 | 1 | 4 | Moderate |
| Kim S. J. et al. (2021) | 1 | 1 | 1 | 0 | 1 | 1 | 5 | High |
| Kim S. Y. et al. (2021) | 1 | 1 | 1 | 0 | 1 | 1 | 5 | High |
| Lanza et al. (2021) | 1 | 1 | 1 | 0 | 1 | 1 | 5 | High |
| Laurier et al. (2021) | 1 | 1 | 1 | 0 | 0 | 1 | 4 | Moderate |
| Lee et al. (2021) | 1 | 1 | 1 | 0 | 1 | 1 | 5 | High |
| Li et al. (2021) | 1 | 1 | 1 | 0 | 0 | 1 | 4 | Moderate |
| Liu et al. (2021) | 1 | 1 | 1 | 0 | 0 | 1 | 4 | Moderate |
| López-Gil et al. (2021) | 1 | 1 | 1 | 0 | 4 | 1 | 5 | Moderate |
| Lu et al. (2020) | 1 | 1 | 1 | 0 | 1 | 1 | 5 | High |
| MacKenzie et al. (2021) | 1 | 1 | 1 | 0 | 0 | 1 | 4 | Moderate |
| Medrano et al. (2021) | 1 | 1 | 1 | 1 | 1 | 1 | 6 | High |
| Mingazova et al. (2021) | 1 | 1 | 1 | 0 | 0 | 1 | 4 | Moderate |
| Mitra et al. (2020) | 1 | 1 | 1 | 0 | 1 | 1 | 5 | High |
| Mitra et al. (2021) | 1 | 1 | 1 | 0 | 0 | 1 | 4 | Moderate |
| Morgül et al. (2020) | 1 | 1 | 1 | 0 | 1 | 1 | 5 | High |
| Ng et al. (2020) | 1 | 1 | 1 | 1 | 1 | 1 | 6 | High |
| Ng et al. (2021) | 1 | 1 | 1 | 0 | 1 | 1 | 5 | High |
| Pombo et al. (2021) | 1 | 1 | 1 | 0 | 1 | 1 | 5 | High |
| Qin J. et al. (2021) | 1 | 1 | 1 | 0 | 1 | 1 | 5 | High |
| Qin Z. et al. (2021) | 1 | 1 | 1 | 1 | 1 | 1 | 6 | High |
| Reséndiz-Aparicio (2021) | 1 | 1 | 1 | 0 | 0 | 1 | 4 | Moderate |
| Sá et al. (2020) | 1 | 1 | 1 | 0 | 0 | 1 | 4 | Moderate |
| Salzano et al. (2021) | 1 | 1 | 1 | 0 | 0 | 1 | 4 | Moderate |
| Schnaiderman et al. (2021) | 1 | 1 | 1 | 0 | 1 | 1 | 5 | High |
| Siachpazidou et al. (2021) | 1 | 1 | 1 | 0 | 1 | 1 | 5 | High |
| Szwarcwald et al. (2021) | 1 | 1 | 1 | 0 | 1 | 1 | 5 | High |
| Tandon et al. (2021) | 1 | 1 | 1 | 0 | 1 | 1 | 5 | High |
| Tornaghi et al. (2021) | 1 | 1 | 1 | 0 | 1 | 1 | 5 | High |
| Ventura et al. (2021) | 1 | 1 | 1 | 0 | 0 | 1 | 4 | Moderate |
| Vuković et al. (2021) | 1 | 1 | 1 | 0 | 0 | 1 | 4 | Moderate |
| Wang, Hao et al. (2021) | 1 | 1 | 1 | 0 | 0 | 1 | 4 | Moderate |
| Wang, Zheng et al. (2021) | 1 | 1 | 1 | 0 | 0 | 0 | 3 | Moderate |
| Wang, Chen et al. (2021) | 1 | 1 | 1 | 0 | 0 | 1 | 4 | Moderate |
| Zhang et al. (2020) | 1 | 1 | 1 | 0 | 1 | 1 | 5 | High |
| Zhu et al. (2021) | 1 | 1 | 1 | 0 | 0 | 1 | 4 | Moderate |

Supplementary Table 10. GRADE score of all outcomes.

| Outcomes | No. of studies | Certainty assessment | | | | | Certainty |
| --- | --- | --- | --- | --- | --- | --- | --- |
|  |  | Risk of Bias | Inconsistency | Indirectness | Imprecision | Publication bias |  |
| PA (pooled) |  | Moderate | Serious^a^ | Not serious | Not serious | Not serious | ⊕⊕⊕◯  Moderate |
| Sleeping patterns |  |  |  |  |  |  |  |
| Sleep duration (pooled) |  | Moderate | Serious^a^ | Not serious | Not serious | Not serious | ⊕⊕⊕◯  Moderate |
| Sleep quality |  | Moderate | Serious^a^ | Not serious | Not serious | Not serious | ⊕⊕⊕◯  Moderate |
| Psychological problems |  | Moderate | Serious^a^ | Not serious | Not serious | Not serious | ⊕⊕⊕◯  Moderate |
| Behavioural problems |  | Moderate | Serious^a^ | Not serious | Not serious | Not serious | ⊕⊕⊕◯  Moderate |
| Meet recommendation guidelines |  |  |  |  |  |  |  |
| PA |  | Moderate | Serious^a^ | Not serious | Not serious | Not serious | ⊕⊕⊕◯  Moderate |
| Sleep duration |  | Moderate | Serious^a^ | Not serious | Not serious | Not serious | ⊕⊕⊕◯  Moderate |

^a^High inconsistency (*I*^2^>90%)

Supplementary Table 11. Sensitivity analysis of all outcomes.

1. PA (all).

| Authors | Points | Lower limit | Upper limit | P-value |
| --- | --- | --- | --- | --- |
| Acosta et al. (2021) | 0.61 | 0.58 | 0.65 | 0.00 |
| Alonso-Martínez et al. (2021) | 0.61 | 0.57 | 0.64 | 0.00 |
| Al-Rahamneh et al. (2021) | 0.61 | 0.58 | 0.64 | 0.00 |
| Alves et al. (2020) | 0.61 | 0.58 | 0.64 | 0.00 |
| Androustsos et al. (2021) | 0.61 | 0.58 | 0.64 | 0.00 |
| Awais et al. (2021) | 0.62 | 0.58 | 0.65 | 0.00 |
| Azoulay et al. (2021) | 0.62 | 0.59 | 0.65 | 0.00 |
| Berasategi et al. (2021) | 0.62 | 0.59 | 0.65 | 0.00 |
| Berki and Pikó et al. (2021) | 0.61 | 0.57 | 0.64 | 0.00 |
| Bingham et al. (2021) | 0.61 | 0.58 | 0.64 | 0.00 |
| Breidokiene et al. (2021) | 0.61 | 0.58 | 0.64 | 0.00 |
| Brzęk et al. (2021) | 0.61 | 0.58 | 0.64 | 0.00 |
| Campbell et al. (2021) | 0.61 | 0.58 | 0.65 | 0.00 |
| Chaffee et al. (2021) | 0.62 | 0.58 | 0.65 | 0.00 |
| Chi et al. (2021) | 0.62 | 0.58 | 0.65 | 0.00 |
| Docimo et al. (2021) | 0.61 | 0.58 | 0.65 | 0.00 |
| Dragun et al. (2020) | 0.62 | 0.59 | 0.65 | 0.00 |
| Dubuc et al. (2020) | 0.61 | 0.58 | 0.64 | 0.00 |
| Dunton et al. (2020) | 0.61 | 0.58 | 0.65 | 0.00 |
| Erades et al. (2020) | 0.61 | 0.58 | 0.65 | 0.00 |
| Ezpeleta et al. (2020) | 0.62 | 0.58 | 0.65 | 0.00 |
| Francisco et al. (2020)1 | 0.61 | 0.58 | 0.64 | 0.00 |
| Francisco et al. (2020)2 | 0.61 | 0.58 | 0.64 | 0.00 |
| Francisco et al. (2020)3 | 0.61 | 0.58 | 0.64 | 0.00 |
| Ghorbani et al. (2021) | 0.61 | 0.57 | 0.64 | 0.00 |
| Gilbert et al. (2021) | 0.61 | 0.58 | 0.65 | 0.00 |
| Guo et al. (2021) | 0.61 | 0.57 | 0.64 | 0.00 |
| Hyunshik et al. (2021) | 0.62 | 0.59 | 0.65 | 0.00 |
| James et al. (2021) | 0.62 | 0.59 | 0.65 | 0.00 |
| Jester and Kong (2021) | 0.62 | 0.58 | 0.65 | 0.00 |
| Jolliff et al. (2021) | 0.62 | 0.58 | 0.65 | 0.00 |
| Jovanović et al. (2021) | 0.61 | 0.58 | 0.64 | 0.00 |
| Kim S. J. et al. (2021) | 0.61 | 0.58 | 0.64 | 0.00 |
| Kim S. Y. et al. (2021)1 | 0.61 | 0.58 | 0.65 | 0.00 |
| Kim S. Y. et al. (2021)2 | 0.61 | 0.58 | 0.65 | 0.00 |
| Lanza et al. (2021) | 0.62 | 0.59 | 0.65 | 0.00 |
| Laurier et al. (2021) | 0.62 | 0.58 | 0.65 | 0.00 |
| Lee et al. (2021)1 | 0.61 | 0.58 | 0.64 | 0.00 |
| Lee et al. (2021)2 | 0.62 | 0.58 | 0.65 | 0.00 |
| Li et al. (2021)1 | 0.61 | 0.57 | 0.64 | 0.00 |
| Li et al. (2021)2 | 0.62 | 0.58 | 0.65 | 0.00 |
| Liu et al. (2021) | 0.61 | 0.58 | 0.65 | 0.00 |
| Lu et al. (2020) | 0.61 | 0.58 | 0.65 | 0.00 |
| Medrano et al. (2021) | 0.61 | 0.58 | 0.65 | 0.00 |
| Mingazova et al. (2021) | 0.61 | 0.58 | 0.64 | 0.00 |
| Mitra et al. (2020)1 | 0.61 | 0.58 | 0.65 | 0.00 |
| Mitra et al. (2020)2 | 0.62 | 0.58 | 0.65 | 0.00 |
| Mitra et al. (2021) | 0.61 | 0.58 | 0.65 | 0.00 |
| Morgül et al. (2020) | 0.62 | 0.58 | 0.65 | 0.00 |
| Ng et al. (2020) | 0.62 | 0.58 | 0.65 | 0.00 |
| Ng et al. (2021)1 | 0.61 | 0.58 | 0.64 | 0.00 |
| Ng et al. (2021)2 | 0.61 | 0.58 | 0.64 | 0.00 |
| Ng et al. (2021)3 | 0.61 | 0.58 | 0.65 | 0.00 |
| Ng et al. (2021)4 | 0.61 | 0.58 | 0.65 | 0.00 |
| Ng et al. (2021)5 | 0.61 | 0.58 | 0.64 | 0.00 |
| Ng et al. (2021)6 | 0.61 | 0.58 | 0.65 | 0.00 |
| Pombo et al. (2021) | 0.61 | 0.58 | 0.64 | 0.00 |
| Qin J. et al. (2021) | 0.61 | 0.58 | 0.64 | 0.00 |
| Qin Z. et al. (2021) | 0.61 | 0.58 | 0.65 | 0.00 |
| Sá et al. (2020) | 0.61 | 0.58 | 0.64 | 0.00 |
| Salzano et al. (2021) | 0.61 | 0.58 | 0.64 | 0.00 |
| Siachpazidou et al. (2021) | 0.62 | 0.58 | 0.65 | 0.00 |
| Szwarcwald et al. (2021) | 0.61 | 0.58 | 0.65 | 0.00 |
| Tandon et al. (2021) | 0.61 | 0.58 | 0.64 | 0.00 |
| Tornaghi et al. (2021) | 0.62 | 0.59 | 0.65 | 0.00 |
| Ventura et al. (2021) | 0.61 | 0.57 | 0.64 | 0.00 |
| Vuković et al. (2021) | 0.62 | 0.58 | 0.65 | 0.00 |
| Wang, Hao et al. (2021) | 0.61 | 0.58 | 0.65 | 0.00 |
| Wang, Zheng et al. (2021) | 0.61 | 0.58 | 0.65 | 0.00 |
| Wang, Chen et al. (2021) | 0.62 | 0.58 | 0.65 | 0.00 |
| Zhang et al. (2020) | 0.61 | 0.58 | 0.65 | 0.00 |
| Zhu et al. (2021) | 0.62 | 0.59 | 0.65 | 0.00 |
| Overall | 0.61 | 0.58 | 0.65 | 0.00 |

1. PA (meeting recommendation guidelines).

| Authors | Points | Lower limit | Upper limit | P-value |
| --- | --- | --- | --- | --- |
| Alonso-Martínez et al. (2021) | 0.40 | 0.38 | 0.42 | 0.000 |
| Al-Rahamneh et al. (2021) | 0.40 | 0.39 | 0.42 | 0.000 |
| Berki and Pikó et al. (2021) | 0.40 | 0.38 | 0.42 | 0.000 |
| Bingham et al. (2021) | 0.40 | 0.39 | 0.42 | 0.000 |
| Breidokiene et al. (2021) | 0.40 | 0.39 | 0.42 | 0.000 |
| Brzęk et al. (2021) | 0.40 | 0.38 | 0.42 | 0.000 |
| Dubuc et al. (2020) | 0.40 | 0.38 | 0.42 | 0.000 |
| Francisco et al. (2020)1 | 0.40 | 0.38 | 0.42 | 0.000 |
| Francisco et al. (2020)2 | 0.40 | 0.38 | 0.42 | 0.000 |
| Francisco et al. (2020)3 | 0.40 | 0.38 | 0.42 | 0.000 |
| Guo et al. (2021) | 0.40 | 0.38 | 0.42 | 0.000 |
| Hyunshik et al. (2021) | 0.41 | 0.40 | 0.43 | 0.000 |
| James et al. (2021) | 0.42 | 0.40 | 0.43 | 0.000 |
| Jester and Kong (2021) | 0.41 | 0.39 | 0.42 | 0.000 |
| Medrano et al. (2021) | 0.41 | 0.39 | 0.42 | 0.000 |
| Morgül et al. (2020) | 0.41 | 0.39 | 0.43 | 0.000 |
| Ng et al. (2021)1 | 0.40 | 0.38 | 0.42 | 0.000 |
| Ng et al. (2021)2 | 0.40 | 0.38 | 0.42 | 0.000 |
| Ng et al. (2021)3 | 0.41 | 0.39 | 0.42 | 0.000 |
| Ng et al. (2021)4 | 0.41 | 0.39 | 0.43 | 0.000 |
| Ng et al. (2021)5 | 0.40 | 0.38 | 0.42 | 0.000 |
| Ng et al. (2021)6 | 0.41 | 0.39 | 0.42 | 0.000 |
| Qin J. et al. (2021) | 0.40 | 0.39 | 0.42 | 0.000 |
| Qin Z. et al. (2021) | 0.40 | 0.38 | 0.43 | 0.000 |
| Tandon et al. (2021) | 0.40 | 0.38 | 0.42 | 0.000 |
| Overall | 0.40 | 0.39 | 0.42 | 0.000 |

1. Sleep duration (all).

| Authors | Points | Lower limit | Upper limit | P-value |
| --- | --- | --- | --- | --- |
| Alonso-Martínez et al. (2021) | 0.35 | 0.27 | 0.45 | 0.004 |
| Al-Rahamneh et al. (2021) | 0.37 | 0.28 | 0.47 | 0.013 |
| Androustsos et al. (2021) | 0.38 | 0.29 | 0.48 | 0.021 |
| Bingham et al. (2021) | 0.38 | 0.28 | 0.48 | 0.016 |
| Brzęk et al. (2021) | 0.38 | 0.29 | 0.48 | 0.018 |
| Docimo et al. (2021) | 0.37 | 0.28 | 0.47 | 0.009 |
| Dubuc et al. (2020) | 0.36 | 0.27 | 0.46 | 0.006 |
| Francisco et al. (2020)1 | 0.39 | 0.30 | 0.49 | 0.028 |
| Francisco et al. (2020)2 | 0.39 | 0.30 | 0.49 | 0.028 |
| Francisco et al. (2020)3 | 0.38 | 0.29 | 0.48 | 0.020 |
| Guo et al. (2021)1 | 0.37 | 0.28 | 0.48 | 0.024 |
| Guo et al. (2021)2 | 0.38 | 0.29 | 0.48 | 0.014 |
| Hyunshik et al. (2021) | 0.38 | 0.29 | 0.48 | 0.019 |
| James et al. (2021) | 0.36 | 0.27 | 0.45 | 0.004 |
| Jovanović et al. (2021) | 0.37 | 0.28 | 0.47 | 0.011 |
| Kim S. Y. et al. (2021) | 0.36 | 0.30 | 0.43 | 0.000 |
| López-Gil et al. (2021) | 0.38 | 0.29 | 0.48 | 0.023 |
| Medrano et al. (2021)1 | 0.36 | 0.27 | 0.45 | 0.005 |
| Medrano et al. (2021)2 | 0.35 | 0.27 | 0.45 | 0.004 |
| Mitra et al. (2020)1 | 0.38 | 0.29 | 0.48 | 0.023 |
| Mitra et al. (2020)2 | 0.37 | 0.28 | 0.47 | 0.014 |
| Mitra et al. (2021)1 | 0.38 | 0.29 | 0.48 | 0.019 |
| Mitra et al. (2021)2 | 0.37 | 0.28 | 0.47 | 0.010 |
| Ng et al. (2021)1 | 0.38 | 0.28 | 0.48 | 0.016 |
| Ng et al. (2021)2 | 0.38 | 0.28 | 0.48 | 0.017 |
| Ng et al. (2021)3 | 0.38 | 0.28 | 0.48 | 0.016 |
| Ng et al. (2021)4 | 0.38 | 0.28 | 0.48 | 0.017 |
| Ng et al. (2021)5 | 0.38 | 0.29 | 0.48 | 0.019 |
| Ng et al. (2021)6 | 0.38 | 0.28 | 0.48 | 0.017 |
| Ng et al. (2021)7 | 0.38 | 0.28 | 0.48 | 0.016 |
| Ng et al. (2021)8 | 0.37 | 0.28 | 0.47 | 0.013 |
| Pombo et al. (2021)1 | 0.38 | 0.29 | 0.48 | 0.024 |
| Pombo et al. (2021)2 | 0.37 | 0.28 | 0.47 | 0.014 |
| Sá et al. (2020)1 | 0.38 | 0.29 | 0.48 | 0.020 |
| Sá et al. (2020)2 | 0.37 | 0.28 | 0.47 | 0.013 |
| Schnaiderman et al. (2021) | 0.38 | 0.29 | 0.48 | 0.022 |
| Ventura et al. (2021) | 0.38 | 0.29 | 0.48 | 0.019 |
| Overall | 0.37 | 0.28 | 0.47 | 0.013 |

1. Sleep duration (meeting recommendation guidelines).

| Authors | Points | Lower limit | Upper limit | P-value |
| --- | --- | --- | --- | --- |
| Alonso-Martínez et al. (2021) | 0.39 | 0.39 | 0.38 | 0.000 |
| Al-Rahamneh et al. (2021) | 0.38 | 0.39 | 0.38 | 0.000 |
| Bingham et al. (2021) | 0.39 | 0.39 | 0.38 | 0.000 |
| Brzęk et al. (2021) | 0.39 | 0.40 | 0.39 | 0.000 |
| Docimo et al. (2021) | 0.38 | 0.39 | 0.38 | 0.000 |
| Dubuc et al. (2020) | 0.35 | 0.36 | 0.35 | 0.000 |
| Guo et al. (2021)1 | 0.39 | 0.39 | 0.38 | 0.000 |
| Hyunshik et al. (2021) | 0.39 | 0.39 | 0.38 | 0.000 |
| James et al. (2021) | 0.38 | 0.38 | 0.37 | 0.000 |
| López-Gil et al. (2021) | 0.39 | 0.40 | 0.38 | 0.000 |
| Medrano et al. (2021)1 | 0.39 | 0.39 | 0.38 | 0.000 |
| Medrano et al. (2021)2 | 0.39 | 0.39 | 0.38 | 0.000 |
| Ng et al. (2021)1 | 0.39 | 0.40 | 0.38 | 0.000 |
| Ng et al. (2021)2 | 0.39 | 0.40 | 0.39 | 0.000 |
| Ng et al. (2021)3 | 0.39 | 0.40 | 0.38 | 0.000 |
| Ng et al. (2021)4 | 0.39 | 0.40 | 0.39 | 0.000 |
| Ng et al. (2021)5 | 0.40 | 0.40 | 0.39 | 0.000 |
| Ng et al. (2021)6 | 0.39 | 0.40 | 0.39 | 0.000 |
| Ng et al. (2021)7 | 0.39 | 0.39 | 0.38 | 0.000 |
| Ng et al. (2021)8 | 0.39 | 0.39 | 0.38 | 0.000 |
| Overall | 0.39 | 0.39 | 0.38 | 0.000 |

1. Sleep quality

| Authors | Points | Lower limit | Upper limit | P-value |
| --- | --- | --- | --- | --- |
| Chi et al. (2021) | 0.34 | 0.27 | 0.42 | 0.000 |
| Dragun et al. (2020) | 0.33 | 0.26 | 0.41 | 0.000 |
| Dubuc et al. (2020) | 0.34 | 0.27 | 0.42 | 0.000 |
| Ezpeleta et al. (2020) | 0.33 | 0.26 | 0.40 | 0.000 |
| Francisco et al. (2020)1 | 0.36 | 0.29 | 0.44 | 0.001 |
| Francisco et al. (2020)2 | 0.36 | 0.28 | 0.43 | 0.000 |
| Francisco et al. (2020)3 | 0.36 | 0.29 | 0.44 | 0.001 |
| Ghanamah and Eghbaria-Ghanamah (2021) | 0.34 | 0.27 | 0.42 | 0.000 |
| Gilbert et al. (2021) | 0.36 | 0.29 | 0.44 | 0.000 |
| López-Gil et al. (2021) | 0.34 | 0.27 | 0.42 | 0.000 |
| MacKenzie et al. (2021) | 0.34 | 0.27 | 0.42 | 0.000 |
| Mitra et al. (2020) | 0.36 | 0.29 | 0.44 | 0.000 |
| Mitra et al. (2021) | 0.36 | 0.29 | 0.43 | 0.000 |
| Reséndiz-Aparicio (2021)1 | 0.35 | 0.28 | 0.43 | 0.000 |
| Reséndiz-Aparicio (2021)2 | 0.34 | 0.27 | 0.41 | 0.000 |
| Salzano et al. (2021) | 0.33 | 0.26 | 0.40 | 0.000 |
| Schnaiderman et al. (2021)1 | 0.36 | 0.28 | 0.43 | 0.000 |
| Schnaiderman et al. (2021)2 | 0.36 | 0.28 | 0.43 | 0.000 |
| Schnaiderman et al. (2021)3 | 0.35 | 0.28 | 0.43 | 0.000 |
| Siachpazidou et al. (2021) | 0.34 | 0.27 | 0.41 | 0.000 |
| Szwarcwald et al. (2021) | 0.35 | 0.27 | 0.43 | 0.001 |
| Ventura et al. (2021)1 | 0.33 | 0.27 | 0.39 | 0.000 |
| Ventura et al. (2021)2 | 0.36 | 0.29 | 0.43 | 0.000 |
| Wang, Zheng et al. (2021) | 0.35 | 0.27 | 0.43 | 0.001 |
| Wang, Chen et al. (2021) | 0.35 | 0.27 | 0.43 | 0.001 |
| Overall | 0.35 | 0.28 | 0.42 | 0.000 |

1. Psychological problems

| Authors | Points | Lower limit | Upper limit | P-value |
| --- | --- | --- | --- | --- |
| Acosta et al. (2021)1 | 0.37 | 0.33 | 0.41 | 0.000 |
| Acosta et al. (2021)2 | 0.37 | 0.33 | 0.41 | 0.000 |
| Acosta et al. (2021)3 | 0.37 | 0.33 | 0.41 | 0.000 |
| Al-Rahamneh et al. (2021)1 | 0.37 | 0.33 | 0.41 | 0.000 |
| Al-Rahamneh et al. (2021)2 | 0.37 | 0.33 | 0.41 | 0.000 |
| Al-Rahamneh et al. (2021)3 | 0.37 | 0.33 | 0.41 | 0.000 |
| Al-Rahamneh et al. (2021)4 | 0.37 | 0.33 | 0.41 | 0.000 |
| Al-Rahamneh et al. (2021)5 | 0.37 | 0.33 | 0.41 | 0.000 |
| Al-Rahamneh et al. (2021)6 | 0.37 | 0.33 | 0.41 | 0.000 |
| Awais et al. (2021) | 0.37 | 0.33 | 0.41 | 0.000 |
| Berasategi et al. (2021)1 | 0.37 | 0.33 | 0.41 | 0.000 |
| Berasategi et al. (2021)2 | 0.37 | 0.33 | 0.41 | 0.000 |
| Berasategi et al. (2021)3 | 0.36 | 0.33 | 0.41 | 0.000 |
| Berki and Pikó et al. (2021) | 0.37 | 0.33 | 0.41 | 0.000 |
| Breidokiene et al. (2021) | 0.37 | 0.33 | 0.41 | 0.000 |
| Chaffee et al. (2021) | 0.37 | 0.33 | 0.41 | 0.000 |
| Chi et al. (2021)1 | 0.37 | 0.33 | 0.41 | 0.000 |
| Chi et al. (2021)2 | 0.37 | 0.33 | 0.41 | 0.000 |
| Dragun et al. (2020) | 0.38 | 0.34 | 0.42 | 0.000 |
| Erades et al. (2020) | 0.37 | 0.33 | 0.41 | 0.000 |
| Ezpeleta et al. (2020) | 0.37 | 0.34 | 0.42 | 0.000 |
| Francisco et al. (2020)1 | 0.37 | 0.33 | 0.41 | 0.000 |
| Francisco et al. (2020)2 | 0.37 | 0.33 | 0.41 | 0.000 |
| Francisco et al. (2020)3 | 0.37 | 0.33 | 0.41 | 0.000 |
| Francisco et al. (2020)4 | 0.37 | 0.33 | 0.42 | 0.000 |
| Francisco et al. (2020)5 | 0.37 | 0.33 | 0.41 | 0.000 |
| Francisco et al. (2020)6 | 0.37 | 0.33 | 0.41 | 0.000 |
| Francisco et al. (2020)7 | 0.37 | 0.33 | 0.41 | 0.000 |
| Francisco et al. (2020)8 | 0.37 | 0.33 | 0.41 | 0.000 |
| Francisco et al. (2020)9 | 0.37 | 0.33 | 0.41 | 0.000 |
| Ghanamah and Eghbaria-Ghanamah (2021)1 | 0.37 | 0.33 | 0.41 | 0.000 |
| Ghanamah and Eghbaria-Ghanamah (2021)2 | 0.37 | 0.33 | 0.41 | 0.000 |
| Ghorbani et al. (2021)1 | 0.37 | 0.33 | 0.41 | 0.000 |
| Ghorbani et al. (2021)2 | 0.37 | 0.33 | 0.41 | 0.000 |
| Ghorbani et al. (2021)3 | 0.37 | 0.33 | 0.41 | 0.000 |
| Gilbert et al. (2021)1 | 0.37 | 0.34 | 0.42 | 0.000 |
| Gilbert et al. (2021)2 | 0.37 | 0.33 | 0.41 | 0.000 |
| Gilbert et al. (2021)3 | 0.37 | 0.34 | 0.42 | 0.000 |
| Gilbert et al. (2021)4 | 0.38 | 0.34 | 0.42 | 0.000 |
| Gilbert et al. (2021)5 | 0.37 | 0.33 | 0.41 | 0.000 |
| Gilbert et al. (2021)6 | 0.37 | 0.33 | 0.41 | 0.000 |
| James et al. (2021) | 0.38 | 0.34 | 0.42 | 0.000 |
| Lee et al. (2021) | 0.37 | 0.33 | 0.41 | 0.000 |
| Li et al. (2021) | 0.37 | 0.33 | 0.41 | 0.000 |
| Mitra et al. (2021)1 | 0.38 | 0.34 | 0.42 | 0.000 |
| Mitra et al. (2021)2 | 0.37 | 0.34 | 0.42 | 0.000 |
| Mitra et al. (2021)3 | 0.38 | 0.34 | 0.42 | 0.000 |
| Mitra et al. (2021)4 | 0.37 | 0.33 | 0.41 | 0.000 |
| Morgül et al. (2020)1 | 0.37 | 0.33 | 0.41 | 0.000 |
| Morgül et al. (2020)2 | 0.37 | 0.33 | 0.41 | 0.000 |
| Morgül et al. (2020)3 | 0.37 | 0.33 | 0.41 | 0.000 |
| Morgül et al. (2020)4 | 0.37 | 0.33 | 0.41 | 0.000 |
| Morgül et al. (2020)5 | 0.37 | 0.33 | 0.41 | 0.000 |
| Morgül et al. (2020)6 | 0.37 | 0.33 | 0.41 | 0.000 |
| Morgül et al. (2020)7 | 0.37 | 0.33 | 0.41 | 0.000 |
| Ng et al. (2021) | 0.38 | 0.34 | 0.42 | 0.000 |
| Qin J. et al. (2021) | 0.37 | 0.33 | 0.41 | 0.000 |
| Qin Z. et al. (2021)2 | 0.38 | 0.33 | 0.42 | 0.000 |
| Qin Z. et al. (2021)3 | 0.38 | 0.33 | 0.42 | 0.000 |
| Qin Z. et al. (2021)4 | 0.38 | 0.33 | 0.43 | 0.000 |
| Qin Z. et al. (2021)5 | 0.38 | 0.33 | 0.42 | 0.000 |
| Salzano et al. (2021) | 0.37 | 0.33 | 0.41 | 0.000 |
| Schnaiderman et al. (2021)1 | 0.37 | 0.33 | 0.41 | 0.000 |
| Schnaiderman et al. (2021)2 | 0.37 | 0.33 | 0.41 | 0.000 |
| Schnaiderman et al. (2021)3 | 0.37 | 0.33 | 0.41 | 0.000 |
| Schnaiderman et al. (2021)4 | 0.37 | 0.33 | 0.41 | 0.000 |
| Schnaiderman et al. (2021)5 | 0.37 | 0.33 | 0.41 | 0.000 |
| Schnaiderman et al. (2021)6 | 0.37 | 0.33 | 0.41 | 0.000 |
| Schnaiderman et al. (2021)7 | 0.37 | 0.33 | 0.41 | 0.000 |
| Schnaiderman et al. (2021)8 | 0.37 | 0.33 | 0.41 | 0.000 |
| Siachpazidou et al. (2021)1 | 0.37 | 0.33 | 0.41 | 0.000 |
| Siachpazidou et al. (2021)2 | 0.37 | 0.33 | 0.41 | 0.000 |
| Siachpazidou et al. (2021)3 | 0.37 | 0.33 | 0.41 | 0.000 |
| Siachpazidou et al. (2021)4 | 0.36 | 0.33 | 0.40 | 0.000 |
| Siachpazidou et al. (2021)5 | 0.36 | 0.33 | 0.40 | 0.000 |
| Szwarcwald et al. (2021) | 0.37 | 0.33 | 0.40 | 0.000 |
| Vuković et al. (2021)1 | 0.37 | 0.33 | 0.41 | 0.000 |
| Vuković et al. (2021)2 | 0.37 | 0.33 | 0.41 | 0.000 |
| Vuković et al. (2021)3 | 0.37 | 0.33 | 0.41 | 0.000 |
| Vuković et al. (2021)4 | 0.37 | 0.33 | 0.41 | 0.000 |
| Wang, Hao et al. (2021)1 | 0.38 | 0.34 | 0.42 | 0.000 |
| Wang, Hao et al. (2021)2 | 0.38 | 0.34 | 0.42 | 0.000 |
| Wang, Hao et al. (2021)3 | 0.38 | 0.34 | 0.42 | 0.000 |
| Wang, Hao et al. (2021)4 | 0.38 | 0.34 | 0.42 | 0.000 |
| Wang, Hao et al. (2021)5 | 0.38 | 0.34 | 0.42 | 0.000 |
| Wang, Hao et al. (2021)6 | 0.38 | 0.34 | 0.42 | 0.000 |
| Wang, Hao et al. (2021)7 | 0.38 | 0.34 | 0.42 | 0.000 |
| Overall | 0.37 | 0.33 | 0.41 | 0.000 |

1. Behavioural problems

| Authors | Points | Lower limit | Upper limit | P-value |
| --- | --- | --- | --- | --- |
| Al-Rahamneh et al. (2021)1 | 0.18 | 0.13 | 0.24 | 0.00 |
| Al-Rahamneh et al. (2021)2 | 0.18 | 0.13 | 0.24 | 0.00 |
| Al-Rahamneh et al. (2021)3 | 0.18 | 0.13 | 0.23 | 0.00 |
| Al-Rahamneh et al. (2021)4 | 0.18 | 0.13 | 0.24 | 0.00 |
| Al-Rahamneh et al. (2021)5 | 0.18 | 0.13 | 0.24 | 0.00 |
| Al-Rahamneh et al. (2021)6 | 0.18 | 0.13 | 0.24 | 0.00 |
| Berasategi et al. (2021)1 | 0.17 | 0.13 | 0.23 | 0.00 |
| Berasategi et al. (2021)2 | 0.18 | 0.14 | 0.24 | 0.00 |
| Berasategi et al. (2021)3 | 0.18 | 0.14 | 0.24 | 0.00 |
| Berasategi et al. (2021)4 | 0.18 | 0.13 | 0.24 | 0.00 |
| Berasategi et al. (2021)5 | 0.18 | 0.13 | 0.24 | 0.00 |
| Berki and Pikó et al. (2021)1 | 0.18 | 0.13 | 0.24 | 0.00 |
| Berki and Pikó et al. (2021)2 | 0.19 | 0.14 | 0.25 | 0.00 |
| Berki and Pikó et al. (2021)3 | 0.18 | 0.13 | 0.24 | 0.00 |
| Chaffee et al. (2021)1 | 0.18 | 0.13 | 0.24 | 0.00 |
| Chaffee et al. (2021)2 | 0.18 | 0.14 | 0.24 | 0.00 |
| Chaffee et al. (2021)3 | 0.18 | 0.13 | 0.24 | 0.00 |
| Chaffee et al. (2021)4 | 0.18 | 0.13 | 0.23 | 0.00 |
| Erades et al. (2020)1 | 0.18 | 0.13 | 0.24 | 0.00 |
| Erades et al. (2020)2 | 0.18 | 0.13 | 0.24 | 0.00 |
| Ezpeleta et al. (2020) | 0.18 | 0.13 | 0.24 | 0.00 |
| Francisco et al. (2020)1 | 0.18 | 0.13 | 0.24 | 0.00 |
| Francisco et al. (2020)2 | 0.18 | 0.13 | 0.24 | 0.00 |
| Francisco et al. (2020)3 | 0.18 | 0.13 | 0.24 | 0.00 |
| Francisco et al. (2020)4 | 0.18 | 0.13 | 0.24 | 0.00 |
| Francisco et al. (2020)5 | 0.18 | 0.13 | 0.24 | 0.00 |
| Francisco et al. (2020)6 | 0.18 | 0.13 | 0.24 | 0.00 |
| Francisco et al. (2020)7 | 0.18 | 0.14 | 0.24 | 0.00 |
| Francisco et al. (2020)8 | 0.18 | 0.13 | 0.24 | 0.00 |
| Francisco et al. (2020)9 | 0.18 | 0.13 | 0.24 | 0.00 |
| Francisco et al. (2020)10 | 0.18 | 0.13 | 0.24 | 0.00 |
| Francisco et al. (2020)11 | 0.18 | 0.13 | 0.24 | 0.00 |
| Francisco et al. (2020)12 | 0.18 | 0.13 | 0.24 | 0.00 |
| Ghanamah et al. (2021)1 | 0.18 | 0.13 | 0.24 | 0.00 |
| Ghanamah et al. (2021)2 | 0.18 | 0.13 | 0.24 | 0.00 |
| Gilbert et al. (2021) | 0.18 | 0.13 | 0.24 | 0.00 |
| James et al. (2021) | 0.18 | 0.14 | 0.24 | 0.00 |
| Kim S. J. et al. (2021)1 | 0.18 | 0.13 | 0.24 | 0.00 |
| Kim S. J. et al. (2021)2 | 0.18 | 0.14 | 0.24 | 0.00 |
| Kim S. Y. et al. (2021)1 | 0.18 | 0.13 | 0.24 | 0.00 |
| Kim S. Y. et al. (2021)2 | 0.19 | 0.14 | 0.25 | 0.00 |
| Morgül et al. (2020)1 | 0.18 | 0.13 | 0.24 | 0.00 |
| Morgül et al. (2020)2 | 0.18 | 0.13 | 0.24 | 0.00 |
| Morgül et al. (2020)3 | 0.18 | 0.13 | 0.24 | 0.00 |
| Morgül et al. (2020)4 | 0.18 | 0.13 | 0.24 | 0.00 |
| Morgül et al. (2020)5 | 0.18 | 0.13 | 0.24 | 0.00 |
| Reséndiz-Aparicio (2021)1 | 0.18 | 0.13 | 0.23 | 0.00 |
| Reséndiz-Aparicio (2021)2 | 0.18 | 0.13 | 0.24 | 0.00 |
| Reséndiz-Aparicio (2021)3 | 0.18 | 0.13 | 0.23 | 0.00 |
| Reséndiz-Aparicio (2021)4 | 0.18 | 0.13 | 0.24 | 0.00 |
| Reséndiz-Aparicio (2021)5 | 0.18 | 0.14 | 0.24 | 0.00 |
| Schnaiderman et al. (2021)1 | 0.18 | 0.13 | 0.24 | 0.00 |
| Schnaiderman et al. (2021)2 | 0.18 | 0.13 | 0.23 | 0.00 |
| Schnaiderman et al. (2021)3 | 0.18 | 0.13 | 0.24 | 0.00 |
| Schnaiderman et al. (2021)4 | 0.18 | 0.13 | 0.24 | 0.00 |
| Wang, Hao et al. (2021)1 | 0.19 | 0.14 | 0.25 | 0.00 |
| Wang, Hao et al. (2021)2 | 0.19 | 0.14 | 0.25 | 0.00 |
| Wang, Hao et al. (2021)3 | 0.19 | 0.14 | 0.25 | 0.00 |
| Wang, Hao et al. (2021)4 | 0.19 | 0.14 | 0.25 | 0.00 |
| Wang, Hao et al. (2021)5 | 0.19 | 0.14 | 0.25 | 0.00 |
| Wang, Hao et al. (2021)6 | 0.19 | 0.14 | 0.25 | 0.00 |
| Wang, Hao et al. (2021)7 | 0.19 | 0.14 | 0.25 | 0.00 |
| Wang, Hao et al. (2021)8 | 0.19 | 0.14 | 0.25 | 0.00 |
| Wang, Hao et al. (2021)9 | 0.19 | 0.14 | 0.25 | 0.00 |
| Wang, Hao et al. (2021)10 | 0.18 | 0.14 | 0.24 | 0.00 |
| Wang, Hao et al. (2021)11 | 0.19 | 0.14 | 0.25 | 0.00 |
| Wang, Hao et al. (2021)12 | 0.19 | 0.14 | 0.25 | 0.00 |
| Wang, Hao et al. (2021)13 | 0.19 | 0.14 | 0.25 | 0.00 |
| Wang, Hao et al. (2021)14 | 0.19 | 0.14 | 0.25 | 0.00 |
| Wang, Hao et al. (2021)15 | 0.19 | 0.14 | 0.25 | 0.00 |
| Wang, Hao et al. (2021)16 | 0.19 | 0.14 | 0.25 | 0.00 |
| Wang, Hao et al. (2021)17 | 0.19 | 0.14 | 0.25 | 0.00 |
| Overall | 0.18 | 0.13 | 0.24 | 0.00 |

# Supplementary Figures

Supplementary Figure 1. Event rate of participants with different PA performance.

Supplementary Figure 2. Event rate of participants with changes in sleep duration.

Supplementary Figure 3. Event rate of participants with decreased sleep quality.

Supplementary Figure 4. Event rate of participants with behavioural problems.

Supplementary Figure 5. Event rate of participants with psychological problems.


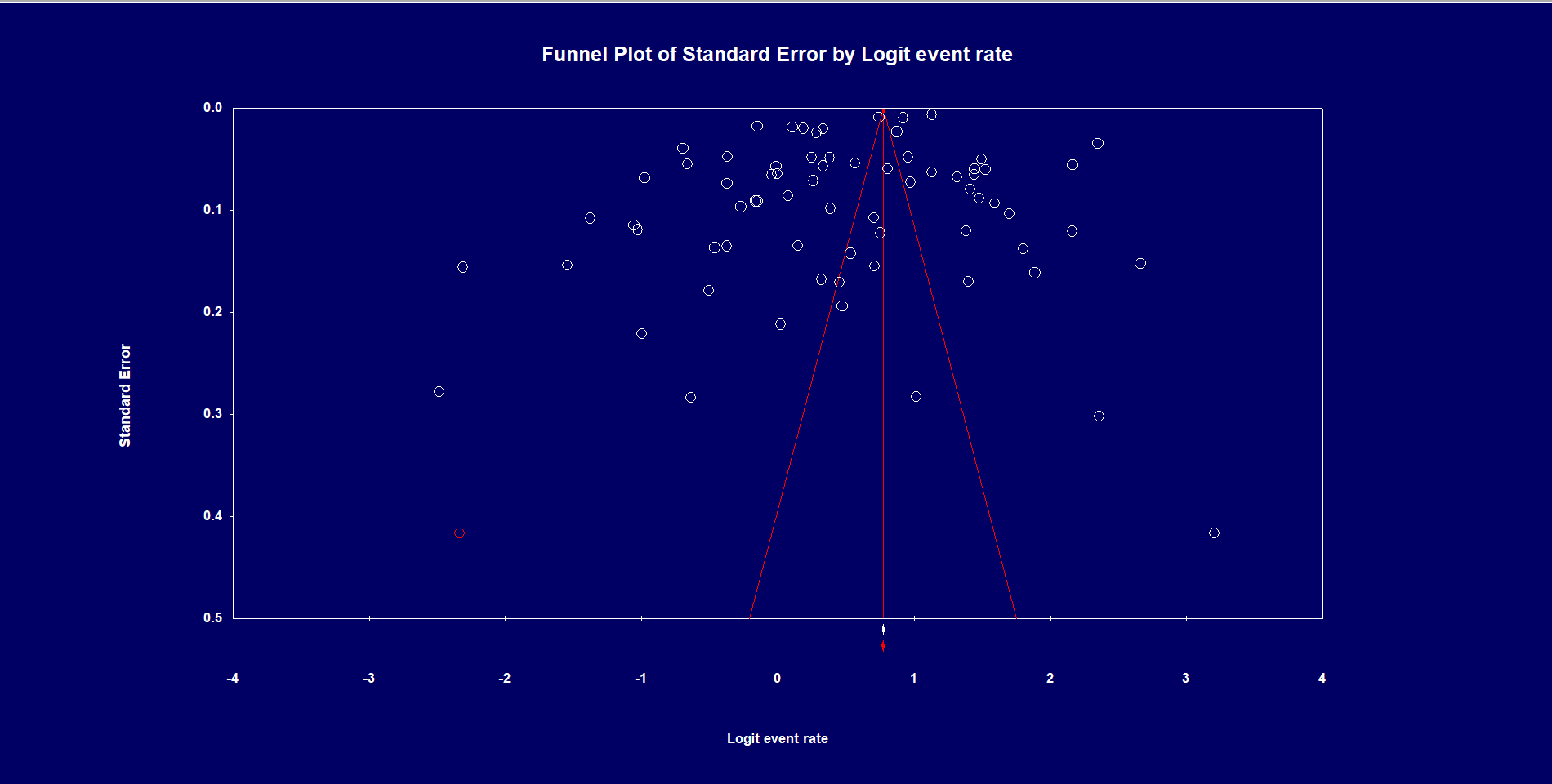


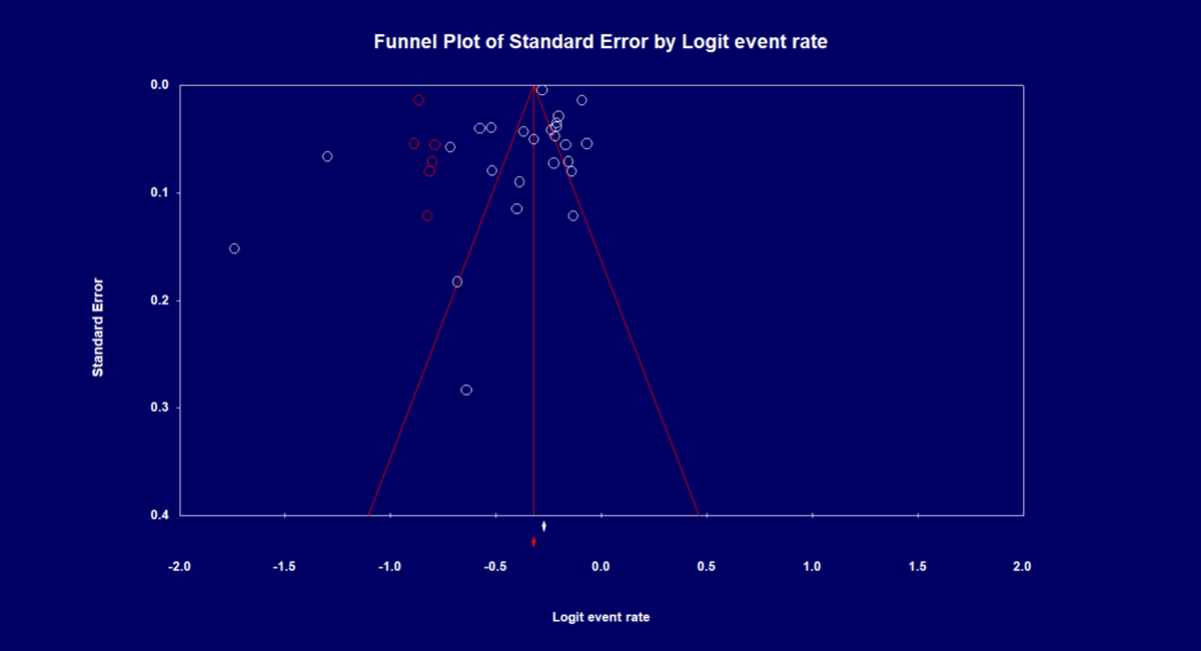


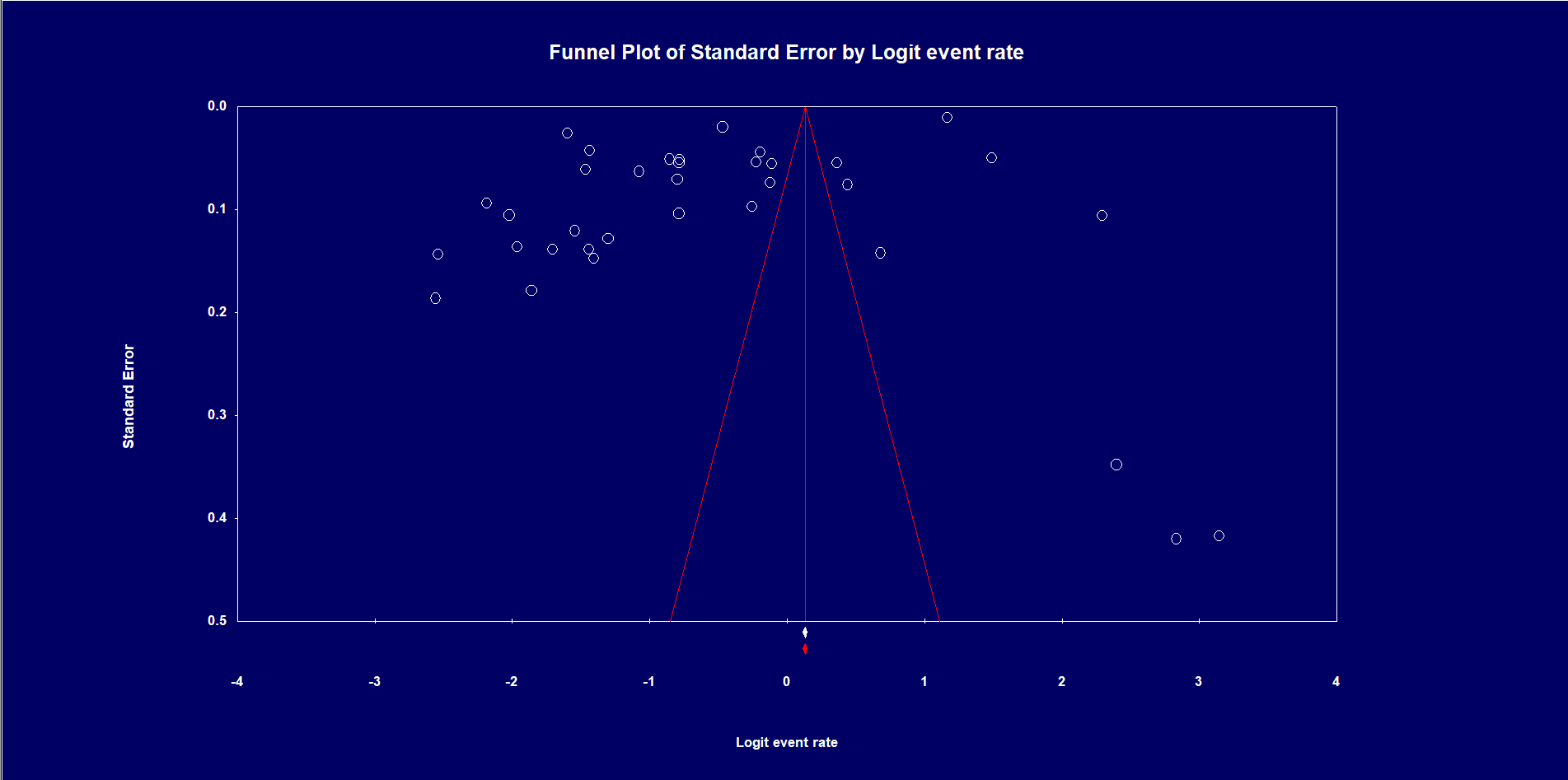


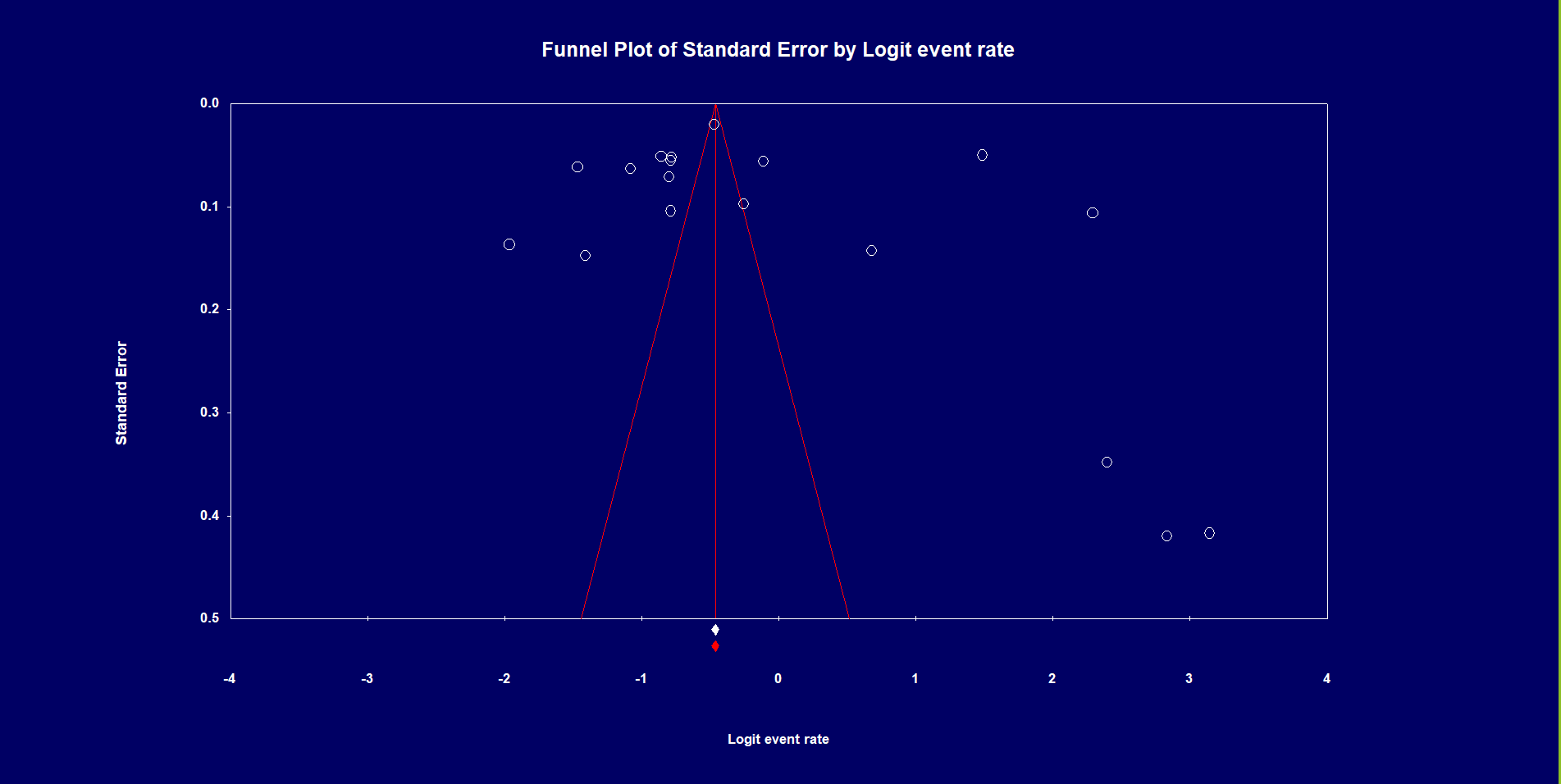


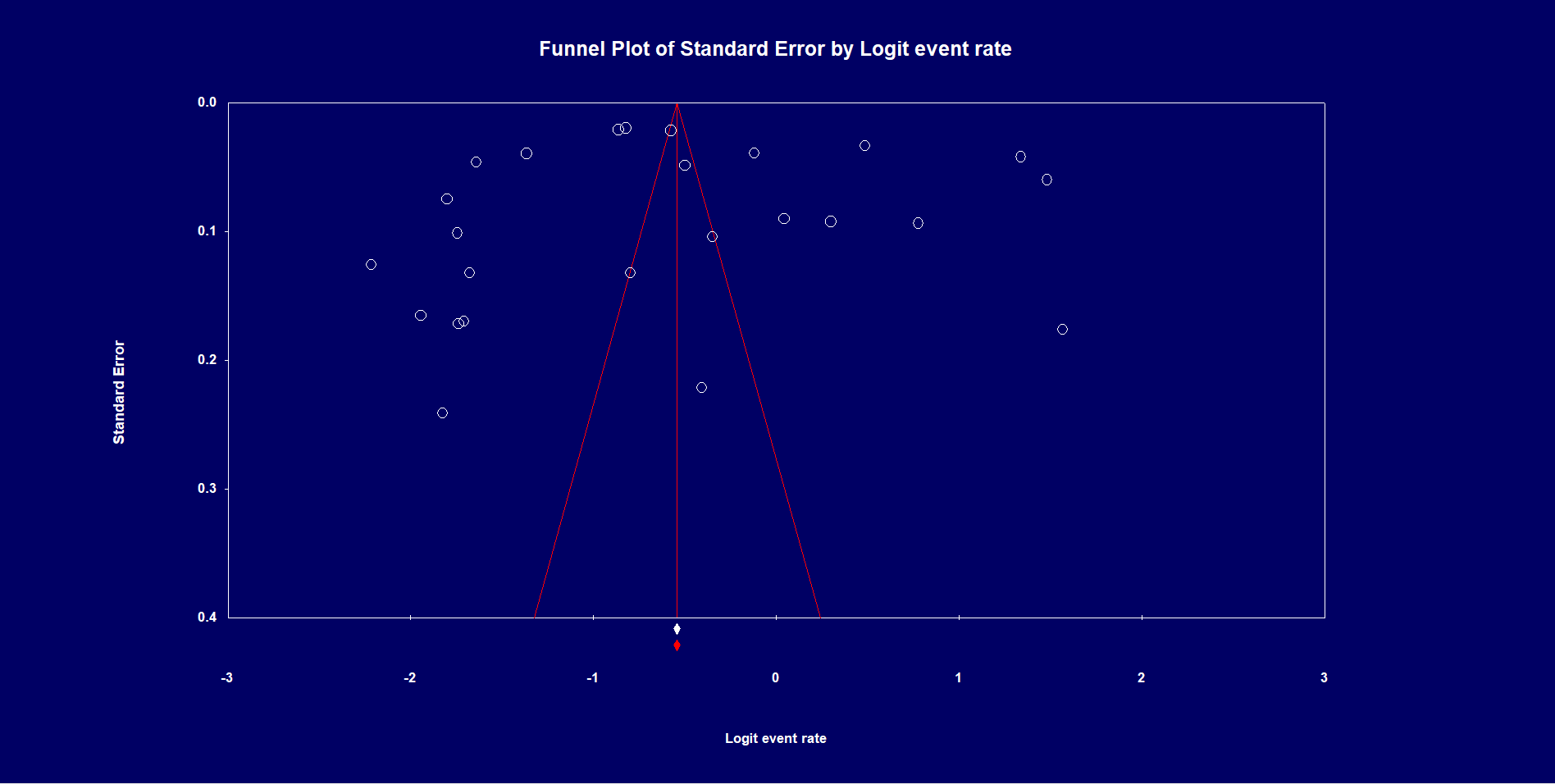


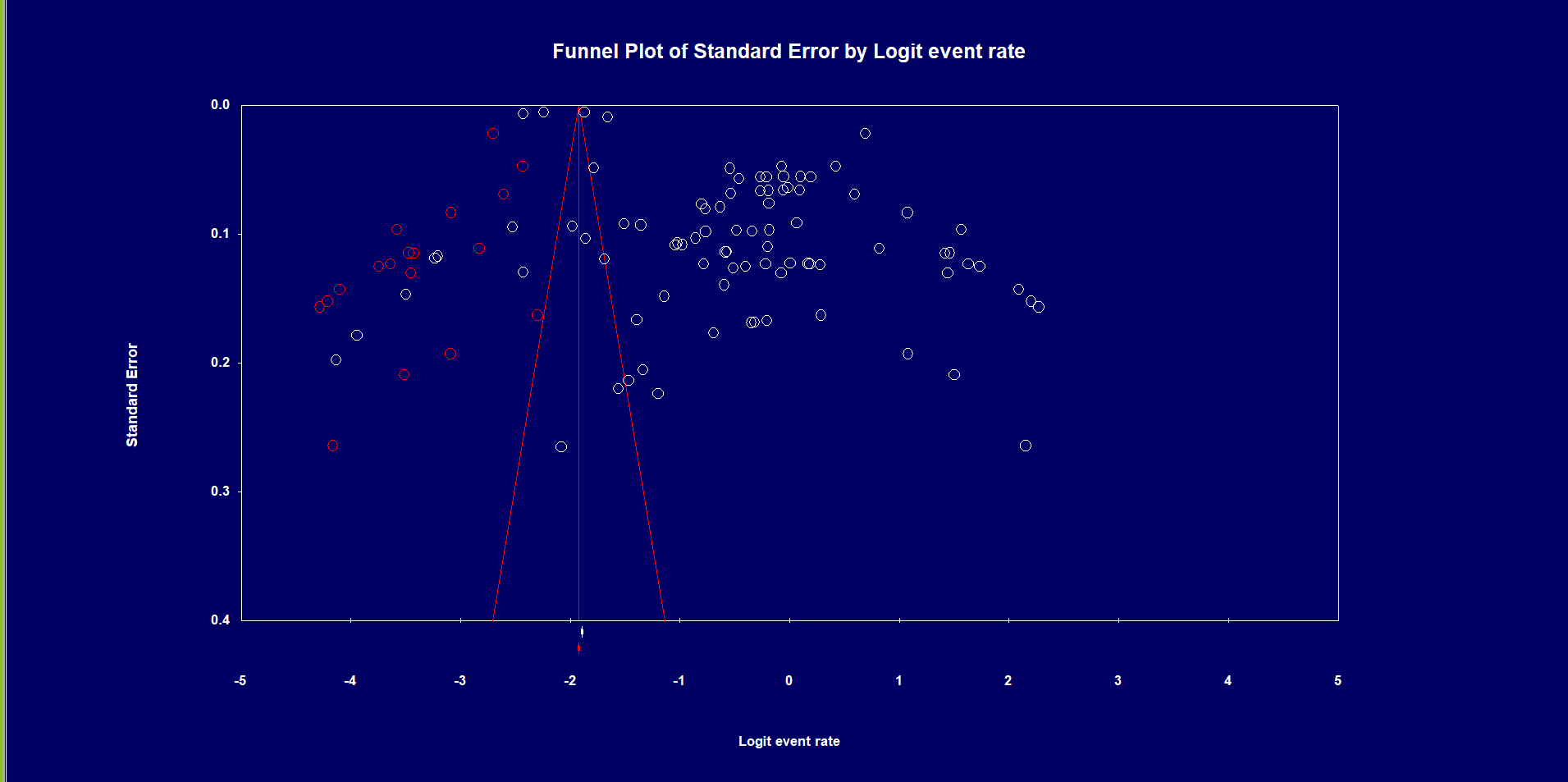


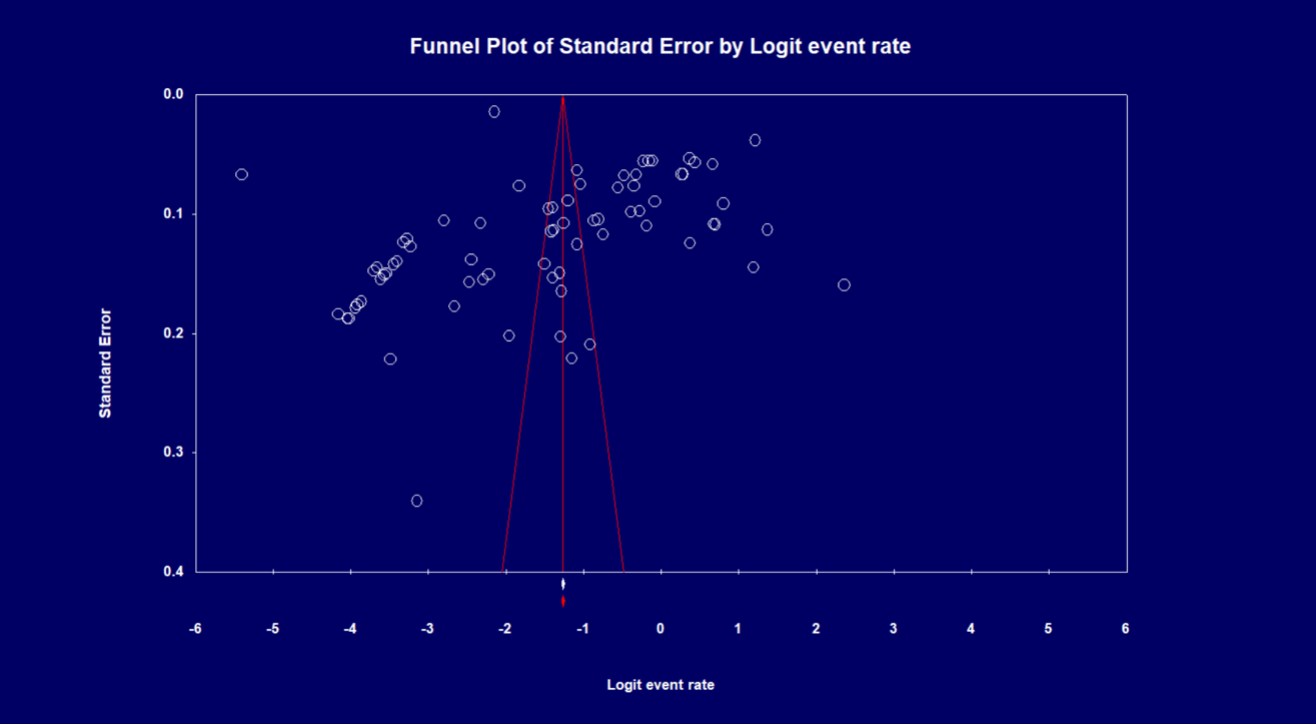


Supplementary Figure 6. Funnel plots of all outcomes. a) PA (all). b) PA (meeting recommendation guidelines). c) Sleep duration (all). d) Sleep duration (meeting recommendation guidelines). e) Sleep quality. f) Psychological problems. g) Behavioural problems.
